# Substrate specificity of plastid phosphate transporters in a non-photosynthetic diatom and its implication in evolution of red alga-derived complex plastids

**DOI:** 10.1038/s41598-020-58082-8

**Published:** 2020-01-24

**Authors:** Daniel Moog, Akira Nozawa, Yuzuru Tozawa, Ryoma Kamikawa

**Affiliations:** 1 Laboratory for Cell Biology, Philipps University Marburg, Karl-von-Frisch-Str. 8, 35032 Marburg, Germany; 2 SYNMIKRO Research Center, Hans-Meerwein-Str. 6, 35032 Marburg, Germany; 3 Proteo-Science Center, Ehime University, Bunkyo-cho, Matsuyama, Ehime 790-8577 Japan; 4 Graduate School of Science and Engineering, Saitama University, Saitama, 338-8570 Japan; 5 Graduate School of Human and Environmental Studies, Kyoto University Kyoto, 606-8501 Japan

**Keywords:** Molecular evolution, Microbiology, Chloroplasts

## Abstract

The triose phosphate transporter (TPT) is one of the prerequisites to exchange metabolites between the cytosol and plastids. In this study, we demonstrated that the four plastid TPT homologues in the non-photosynthetic diatom *Nitzschia* sp. NIES-3581 were highly likely integrated into plastid envelope membranes similar to counterparts in the model photosynthetic diatom *Phaeodactylum tricornutum*, in terms of target membranes and C-terminal orientations. Three of the four *Nitzschia* TPT homologues are capable of transporting various metabolites into proteo-liposomes including triose phosphates (TPs) and phosphoenolpyruvate (PEP), the transport substrates sufficient to support the metabolic pathways retained in the non-photosynthetic diatom plastid. Phylogenetic analysis of TPTs and closely related transporter proteins indicated that diatoms and other algae with red alga-derived complex plastids possess only TPT homologues but lack homologues of the glucose 6-phosphate transporter (GPT), xylulose 5-phosphate transporter (XPT), and phosphoenolpyruvate transporter (PPT). Comparative sequence analysis suggests that many TPT homologues of red alga-derived complex plastids potentially have the ability to transport mainly TPs and PEP. TPTs transporting both TPs and PEP highly likely mediate a metabolic crosstalk between a red alga-derived complex plastid and the cytosol in photosynthetic and non-photosynthetic species, which explains the lack of PPTs in all the lineages with red alga-derived complex plastids. The PEP-transporting TPTs might have emerged in an early phase of endosymbiosis between a red alga and a eukaryote host, given the broad distribution of that type of transporters in all branches of red alga-derived complex plastid-bearing lineages, and have probably played a key role in the establishment and retention of a controllable, intracellular metabolic connection in those organisms.

## Introduction

The first photosynthetic plastids emerged through endosymbiosis and organellar establishment of an ancestral cyanobacterium in a heterotrophic eukaryote, an evolutionary event which is called primary endosymbiosis^1–3^. Organisms that evolved from the first photosynthetic eukaryote are highly likely to be red algae, glaucophytes, and Chloroplastida, the latter group is comprised of the green algae and land plants currently^4,5^. The plastids of these organisms are called primary plastids^4,5^. The primary plastids of red and green algae have successively been transferred into other phagotrophic eukaryotes through secondary or higher order endosymbioses, resulting in “complex plastids” in various branches of the eukaryotic tree of life^4–8^. Establishment of a controllable metabolic connection between a host and an endosymbiont is one of the essential evolutionary steps in the course of an endosymbiont becoming an organelle^9^.

Photosynthetic plastids have a number of roles for cell viability of plants and algae such as conversion of solar power to biochemical energy, e.g., ATP and NADPH, biosynthesis of sugar phosphates by fixing carbon dioxide, and the supply of various key metabolites^10^. Especially, sugar phosphates synthesized through the Calvin Benson cycle contribute to cytosolic or mitochondrial metabolic pathways and energy storage^10–12^. Therefore, the sugar phosphates have to be transported across the plastid membranes through a specific transporter family called plastid phosphate transporters (pPT), which function as antiport systems using inorganic phosphate and phosphorylated sugar compounds as counter-substrates^9,13^.

Functions of pPTs in double membrane-bound, primary plastids have been well studied. In the vascular plant *Arabidopsis thaliana*, the inner membrane of its primary plastid possesses various pPTs such as a glucose 6-phosphate transporter (GPT), a xylulose phosphate transporter (XPT), a phosphoenolpyruvate transporter (PPT), and a triose phosphate transporter (TPT)^9^. GPT has a broad substrate specificity accepting phosphorylated C3 and C6 compounds^14^. PPT is capable of transporting PEP and 2-phosphoglycerate (2-PGA), but not triose phosphates (TPs) nor 3-phosphoglycerate (3-PGA)^15^. TPs, such as dihydroxyacetone phosphate (DHAP), and 3-PGA are transported through the TPT^9^. The vascular plant-specific plastid transporter XPT is capable of transporting xylulose 5-phosphate (X5-P) and only a small amount of erythrose 4-phosphate (E4-P)^16^. Similar to plants, the GPT, PPT, and TPT homologues were identified in the red alga *Galdieria sulphuraria*^13,17^, although the substrate ranges of these pPTs are more restricted when compared to those of *A. thaliana*. The GPT homologue of *G. sulphuraria* is capable of transporting a wide range of sugar phosphates except for phosphorylated glucose and phosphorylated ring sugar moieties^13,17^, while the TPT and the PPT of this red alga are capable of transporting specifically DHAP and PEP, respectively, but not 3-PGA^17^.

Red algal TPT homologues are also encoded in genomes of algae with red alga-derived complex plastids, such as Cryptophyta, Haptophyta, Ochrophyta, Dinoflagellata, Colpodellida, and Apicomplexa, although other pPTs such as GPT and PPT homologues have not been identified^18^. It has been proposed that the TPT homologues in red alga-derived complex plastids originated via endosymbiotic gene transfer (EGT) of a TPT gene provided by the endosymbiotic red alga^18,19^. So far, substrate-specificity of TPT homologues in those complex plastids was investigated with transporters of non-photosynthetic plastid-derived organelles called apicoplasts in apicomplexan parasites^20^ and with photosynthetic plastid membranes (and proteo-liposomes) of a cryptophyte^21^. One or two apicoplast membrane-localized TPT homologues are encoded in the genome of *Plasmodium falciparum* and *Toxoplasma gondii* and importantly, they are capable of transporting PEP as well as DHAP and to a lesser extent 3-PGA. Thus, the apicoplast TPT homologues are distinguishable from TPTs in primary plastids with respect to the specificity for PEP^20,22,23^. Similarly, substrate transport through proteo-liposome membranes harboring membrane proteins of the cryptophyte *Guillardia theta* was investigated and indicated TP- and PEP-transport activity^9,21^.

Bacillariophyceae (or diatoms; Ochrophyta) are a eukaryotic algal group whose members possess red alga-derived complex plastids bound by four membranes. Previous studies identified at least four plastid-localized TPT homologues, called TPT1, TPT2, TPT4a, and TPT4b, in this group, present in the outermost, the second outermost, and the innermost plastid envelope membrane, respectively^18^. The self-assembling GFP system can be used as a tool to study membrane protein topology^24^. The C-termini of TPT1, TPT4a, and TPT4b were found to be localized in the cytosol and the stroma (4a and 4b), respectively, while localization of the C-terminus of TPT2 (second outermost membrane) could not be determined by this method^18^. Although a model invokes that photosynthetic diatom plastids export photosynthesis-derived TPs into the cytosol [e.g.^25,26^], it remains unclear what sugar phosphates are exported from and imported into diatom plastids in detail as studies dealing with the substrate specificity of the TPTs located in the plastid membranes are lacking so far.

*Nitzschia* sp. NIES-3581 is a heterotrophic diatom that has evolved from a photosynthetic ancestor by losing photosynthesis^27^. The plastids of *Nitzschia* sp. NIES-3581 were found to be incapable of carbon fixation via the Calvin Benson cycle but still retain various biochemical functions such as ATP metabolism and biosynthesis of heme, fatty acids, amino acids, and Fe-S cluster^28–30^. To fuel those metabolic pathways, we have hypothesized earlier that carbon and energy sources should be imported into the plastid from the cytosol^29^. TPT homologues were localized in the plastid membranes and thought to contribute to sugar phosphate import into the non-photosynthetic plastids^29^. However, it remains unclear in which plastid membrane they are exactly localized, how the TPTs are integrated into their target membranes (orientation), and which substrates are transported by the putative transporters.

In this study, we determined the localization and orientation of the TPT homologues of *Nitzschia* sp. NIES-3581 via the self-assembling GFP system and examined the substrate specificity of the TPT homologues. We discuss the roles of the TPTs in the non-photosynthetic diatom plastid as well as the first, red alga-derived complex plastid in terms of functions, phylogenetic distribution, and evolution of TPT homologues.

## Results

### Localization of C-termini of plastid TPTs in a non-photosynthetic diatom

Four TPT homologues, described as NspTPT1, NspTPT2, NspTPT4a, and NspTPT4b, were localized in the complex plastid of the non-photosynthetic diatom *Nitzschia* sp. NIES-3581 and predicted as integral membrane proteins^29^. In order to further gain insight into the exact localization and topological conformation of the TPTs, we investigated the localization of their C-termini with the self-assembling GFP system as performed previously with the model diatom *P. tricornutum*^18^. In brief, a protein of interest is expressed as a recombinant protein C-terminally fused with a partial, non-fluorescent GFP fragment (GFP (S11)) in *P. tricornutum* simultaneously with another recombinant protein or targeting sequence that is a marker for a particular cellular compartment C-terminally fused with the other GFP fragment (GFP (S1–10)). In case the two GFP fragments are co-localized in the same subcellular compartment, green fluorescence will emerge, which can be detected via fluorescence microscopy. In other words, if green fluorescence is observed, the C-terminus of a membrane protein of interest is localized in the compartment in which the marker protein localizes.

**Figure 1:**
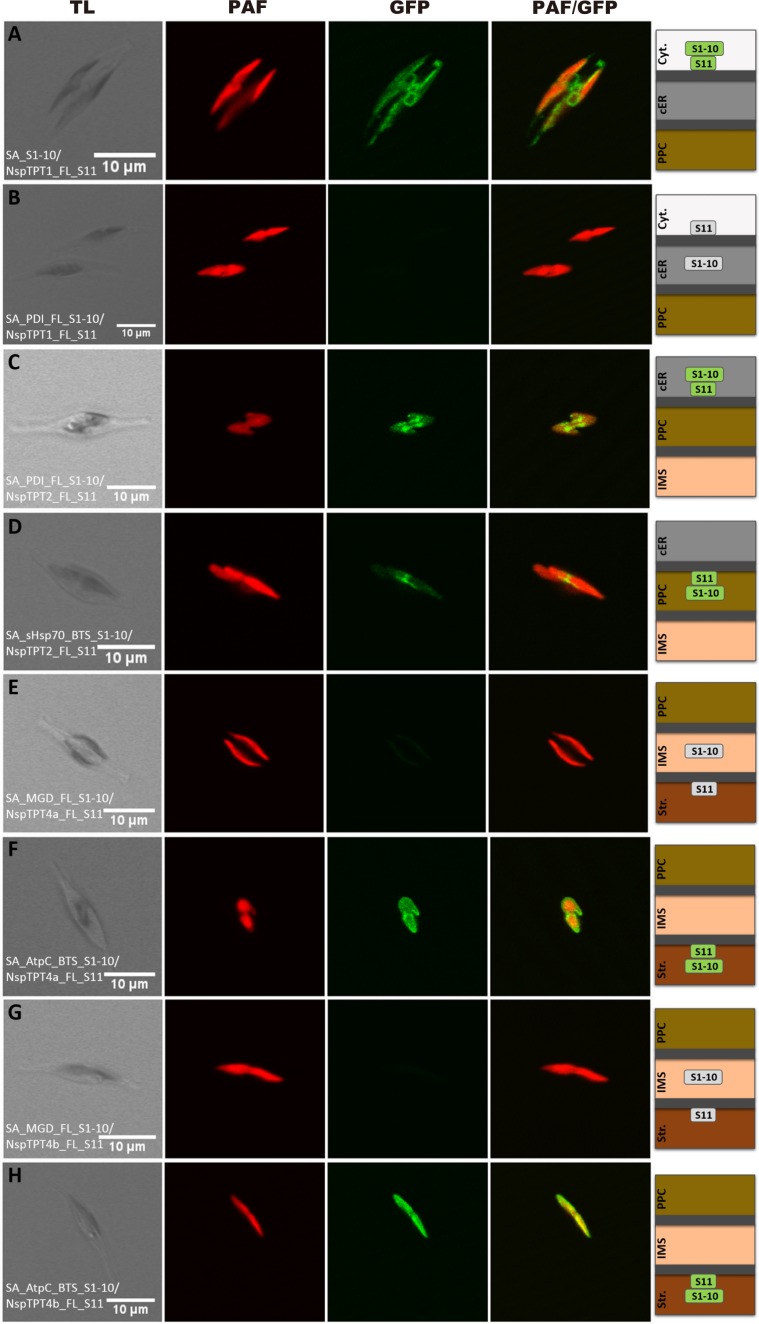
Self-assembling GFP analysis of non-photosynthetic *Nitzschia* sp. plastid TPT homologues in *Phaeodactylum tricornutum*. Simultaneous expression of NspTPT1 fused to GFP(S11) with a cytosolic GFP(S1–10) led to a cER-characteristic fluorescence pattern (**A**). No fluorescence could be observed after expression of NspTPT1:GFP(S11) with the ER/cER marker (PDI) fused to GFP(S1–10) (**B**). Fluorescence signals were obtained upon expression of the NspTPT2:GFP(S11) fusion proteins with both the ER/cER- (**C**) and the PPC-marker fused to GFP(S1–10) (**D**). Both NspTPT4a:GFP(S11) (**E**) and NspTPT4b:GFP(S11) (**G**) did not show a fluorescence signal when expressed with the IMS marker MGD1 fused to GFP(S1–10), whereas a clear GFP signal circling the plastid autofluorescence could be observed when NspTPT4a:GFP(S11) (**F**) and NspTPT4b:GFP(S11) (**H**) were expressed with the stromal-targeted AtpC:GFP(S1–10). TL, transmitted light; PAF, plastid autofluorescence; GFP, enhanced green fluorescent protein; PAF/GFP, overlay of plastid and GFP fluorescence; Cyt., cytosol; cER, chloroplast endoplasmic reticulum; PPC, periplastidal compartment; IMS, intermembrane space; Str., plastid stroma; scale bar represents 10 µm.

To investigate localization of the C-terminal region of NspTPT1, which was predicted to be localized in the outermost membrane of the complex plastid, we expressed NspTPT1:GFP (S11) in *P. tricornutum* cells also expressing cytosolic GFP (S1–10). We observed green fluorescence that partly co-localized with chlorophyll autofluorescence but showed a ring-like structure in direct proximity to the plastid as observed in our previous study (Fig. 1A; see also^29^). This indicates cytosolic localization of the C-terminus of NspTPT1 inserted in the outermost membrane of the complex plastid – the chloroplast ER (cER) membrane. To confirm this, we also performed the same experiments in the *P. tricornutum* cells expressing protein disulfide isomerase (PDI):GFP (S1–10), which localizes in the ER-/cER-lumen – the intermembrane space of the outermost and the second outermost plastid membrane. We did not observe any green fluorescence in this case (Fig. 1B), supporting cytosolic localization of the C-terminal region of NspTPT1.

While NspTPT1 localizes in the outermost membrane of the plastid, NspTPT2, NspTPT4a, and NspTPT4b have been predicted to localize in the second outermost (TPT2) and the innermost membrane (TPT4a and 4b), respectively^29^. Those three TPTs were examined in a similar manner as NspTPT1. In addition to PDI:GFP(S1–10), the plastid-targeting sequence of the PPC-localized heat shock protein 70 (PPC-Hsp70):GFP (S1–10), plastid monogalactosyldiacylglycerol synthase (MGD1):GFP (S1–10), and the plastid-targeting sequence of AtpC:GFP (S1–10) were used as maker proteins for localization in the periplastidal compartment (PPC) between the second outermost and the second innermost membranes (PPC-Hsp70), the intermembrane space between the second innermost and innermost membranes (MGD1), and the stroma (AtpC), respectively^18^. In the experiments for NspTPT4a and NspTPT4b, green fluorescence was exclusively observed in the *P. tricornutum* cells expressing either of the two TPTs (4a or 4b) fused to GFP(S11) together with AtpC:GFP(S1–10), in the fluorescence microscopic observation (Fig. 1F,H). In contrast, we observed no green fluorescence when NspTPT4a:GFP(S11) (Fig. 1E) or NspTPT4b:GFP(S11) (Fig. 1G) were co-expressed with MGD1:GFP(S1–10), respectively, indicating that C-termini of NspTPT4a and NspTPT4b localize in the stroma. However, we obtained inconclusive results in experiments with NspTPT2, which was predicted to localize in the periplastidal membrane (PPM; the second outermost membrane); GFP fluorescence was detected in both cases when NspTPT2:GFP(S11) was co-expressed in *P. tricornutum* cells expressing either PDI:GFP(S1–10) or PPC-Hsp70:GFP(S1–10)(Fig. 1C,D). Therefore, localization of the C-terminus of NspTPT2 could not be determined. Interestingly, similar observations have also been reported in previous studies in which PPM-localized TPT2 and Derlin proteins of *P. tricornutum* were investigated in the same manner^18,31^ and indicate the limitation of the self-assembling GFP system for topology determination of PPM proteins.

The localization experiments of the four TPT C-terminal regions support our previous prediction that NspTPT1 localizes to the outermost, NspTPT2 to the second outermost, and NspTPT4a and NspTPT4b to the innermost membrane of the complex plastid of *Nitzschia* sp. NIES-3581. It is worth noting that the localization and C-terminal orientation of NspTPT1, NspTPT4a, and NspTPT4b are highly likely comparable to those of TPT1, TPT4a, and TPT4b of the model photosynthetic diatom *Phaeodactylum tricornutum*^18^. However, the C-terminal orientation of TPT2 integrated into the second outermost membrane of diatom complex plastids remains to be elucidated in both *P. tricornutum* and *Nitzschia* sp. NIES-3581.

### Construction of TPT-proteo-liposomes and analysis of substrate specificity

**Figure 2:**
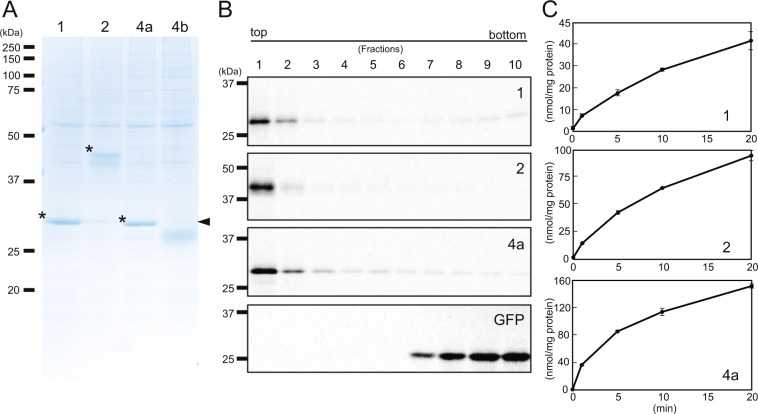
Synthesis of TPT-proteo-liposomes. (**A**) Synthesis of TPT homologues of *Nitzschia* sp. NIES-3581. NspTPT homologues were synthesized by a wheat germ cell-free system in the presence of liposomes. After the synthesis reaction, the mixtures were centrifuged. The pellet fractions of the reaction mixtures were subjected to SDS-PAGE and stained with Coomassie Brilliant Blue. *TPT proteins synthesized with the correct molecular masses. Arrowhead: expected band size, indicating that the synthesized TPT4b is truncated. 1: TPT1, 2: TPT2, 4a: TPT4a, 4b: TPT4b. (**B**) Incorporation of synthesized TPT homologues into liposomes. NspTPT homologues and GFP synthesized by a wheat germ cell-free system in the presence of liposomes and [^14^C] Leu were subjected to Accudenz density gradient ultracentrifugation and fractions were collected from the top of the tube. Each of the fractions (1–10) was subjected to SDS-PAGE and autoradiography. (**C**) Time course of the incorporation of [^32^P] phosphate into the reconstituted proteo-liposomes preloaded with 30 mM of phosphate. The uptake of [^32^P] phosphate was measured as described in the Materials and Methods section. Bars: standard deviation in triplicate experiments.

To investigate the substrate specificity of the plastid TPTs of *Nitzschia* sp. NIES-3581 (NspTPTs), we first constructed artificial proteo-liposomes in which NspTPTs were integrated. We synthesized NspTPT1, NspTPT2, NspTPT4a, and NspTPT4b proteins through a cell-free translation system and had them integrated into the artificial liposomes, as performed previously^32^. We subjected the isolated proteo-liposomes to SDS-PAGE. In the SDS-PAGE run, the synthesized NspTPT1, NspTPT2, and NspTPT4a appeared as bands with expected molecular masses (Fig. 2A). In contrast, the synthesized NspTPT4b was found to be explicitly smaller than the expected molecular mass (Fig. 2A). For the truncated NspTPT4b no transport activity for phosphate and other compounds was detected (Supplementary fig. S1 and see below for details of the experiment), indicating that the synthesized incomplete protein was non-functional. Based on the non-expected mass shift for TPT4b observed on the SDS gel, we assumed that the second or the third in-frame methionine codon might serve as the translation start or that post-translational proteolysis might occur. As a consequence, we synthesized NspTPT4b after the second and the third in-frame methionine codons had been replaced by GTG (valine) codons (Supplementary fig. S1), or synthesized it at a lower temperature, i.e., 14 °C, to restrict post-translational proteolysis. Nevertheless, NspTPT4b still appeared as truncated (Supplementary fig. S1), suggesting a post-translational cleavage due to heterologous expression of the diatom sequence in the wheat system or due to other unknown reasons. Therefore, we decided to use only NspTPT1, NspTPT2, and NspTPT4a for the following experiments.

To test if the three TPTs in the proteo-liposome membranes were functional, we first verified that the synthesized TPTs are associated with the supplemented liposomes. Three TPTs were synthesized in a wheat cell-free system in the presence of liposomes and [^14^C]-Leu (Fig. 2B), and the translation mixtures were fractionated by Accudenz density gradient ultracentrifugation^32^. Each synthesized TPT was detected in the upper fraction, which contains liposomes, indicating each synthesized TPT was actually incorporated into liposomes (Fig. 2B). In contrast, synthesized GFP, a soluble protein used for a negative control experiment, did not associate with liposomes as it was found in the lower fractions (Fig. 2B). We evaluated the activity of the phosphate-phosphate counter exchange as TPTs are phosphate antiporters^33^, using [^32^P] phosphate. Each of the three TPTs was synthesized by liposome-supplemented cell-free synthesis and reconstituted into liposomes containing 30 mM unlabeled phosphate. As shown in Fig. 2C, the uptake of [^32^P] phosphate into proteo-liposomes containing NspTPT1, NspTPT2, and NspTPT4a increased for at least 20 min (Fig. 2C). These results indicated that all of the three TPTs were reconstituted in proteo-liposomes as functional membrane proteins possessing transport activity for inorganic phosphate.

To determine the substrate specificities of NspTPT1, NspTPT2, and NspTPT4a, we measured the uptake of [^32^P] phosphate into proteo-liposomes preloaded with different counter-substrates (phosphate, DHAP, 3-PGA, PEP, and G6-P), according to^32^. As shown in Table 1, transport activity of G6-P was only 1.5–9.1% of the phosphate-phosphate counter exchange activity for all the three TPTs investigated. When considering the 2.9–10.4% activity even in the negative control, the TPTs highly likely have no specificity for G6-P. In contrast, transport activity of the other tested substrates was observed for all of the three TPTs. In liposomes containing NspTPT1, the substrates DHAP, 3-PGA, and PEP were transported in almost comparable quantities (37.7–49.4%). Similar trends of substrate transport were observed for proteo-liposomes with NspTPT2 (20.7–27.9%). However, transport activities of DHAP, 3-PGA, and PEP were determined to be different for NspTPT4a. The highest activity was observed for PEP transport (61.5%), and transport activity of DHAP was 26.4%, which is approximately two to three times lower than that of PEP. Transport of 3-PGA was also observed to be only 7.7%, a much lesser extent than for PEP and DHAP.

**Table 1:** TPT transport activity for [^32^P] phosphate with different counter-substrates.

	NspTPT1		NspTPT2		NspTPT4a	
	nmol/mg protein/min	%^a^	nmol/mg protein/min	%^a^	nmol/mg protein/min	%^a^
—	0.2 (0.0)	10.4	0.2 (0.1)	6.9	0.4 (0.1)	2.9
Pi	2.3 (0.8)	100	2.9 (0.5)	100	15.0 (0.9)	100
PEP	1.1 (0.3)	49.4	0.7 (0.6)	23.1	9.2 (1.0)	61.5
DHAP	0.9 (0.2)	37.7	0.6 (0.0)	20.7	4.0 (0.3)	26.4
3-PGA	1.1 (0.3)	47.2	0.8 (0.1)	27.9	1.2 (0.2)	7.7
G6-P	0.2 (0.2)	9.1	0.1 (0.0)	2.1	0.2 (0.1)	1.5

—: Negative control in which proteo-liposomes include no internal substrate.

Parentheses: standard deviations. ^a^Ratio of transport activity for each substrate to that for Pi.

### Phylogenetic relationships among TPT homologues of eukaryotes bearing red alga-derived complex plastids

In order to gain deeper insight into the evolution of pPTs, we performed phylogenetic analyses of pPT homologues retrieved from all the lineages with red alga-derived complex plastids, i.e., Ochrophyta, Haptophyta, Cryptophyta, Apicomplaxa, Colpodellida, and Dinoflagellata. Plastid PPT, GPT, XPT, and TPT homologues of land plants, green algae, and red algae were also included in the analyses. Our analyses revealed that, as indicated in the previous studies [e.g.^18,29^], with the exception of a subset of dinoflagellates, all the lineages with the red alga-derived complex plastids possessed red algal TPT homologues that were monophyletic with TPT homologues of red algae (Supplementary Fig. S2). The dinoflagellates seem to possess TPT homologues phylogenetically more closely related to counterparts of Chloroplastida and red algae (dinoflagellate clade I and II; Supplementary Fig. S2). However, the branch lengths of these dinoflagellate homologues in relation are longer than others, indicating that the Chloroplastida-dinoflagellates and/or red algae-dinoflagellates relationships might be an artifact and that the results have to be interpreted with caution. Again, we could not detect any pPT homologues closely related to GPT, XPT, nor PPT homologues in the lineages with red alga-derived complex plastids.

**Figure 3:**
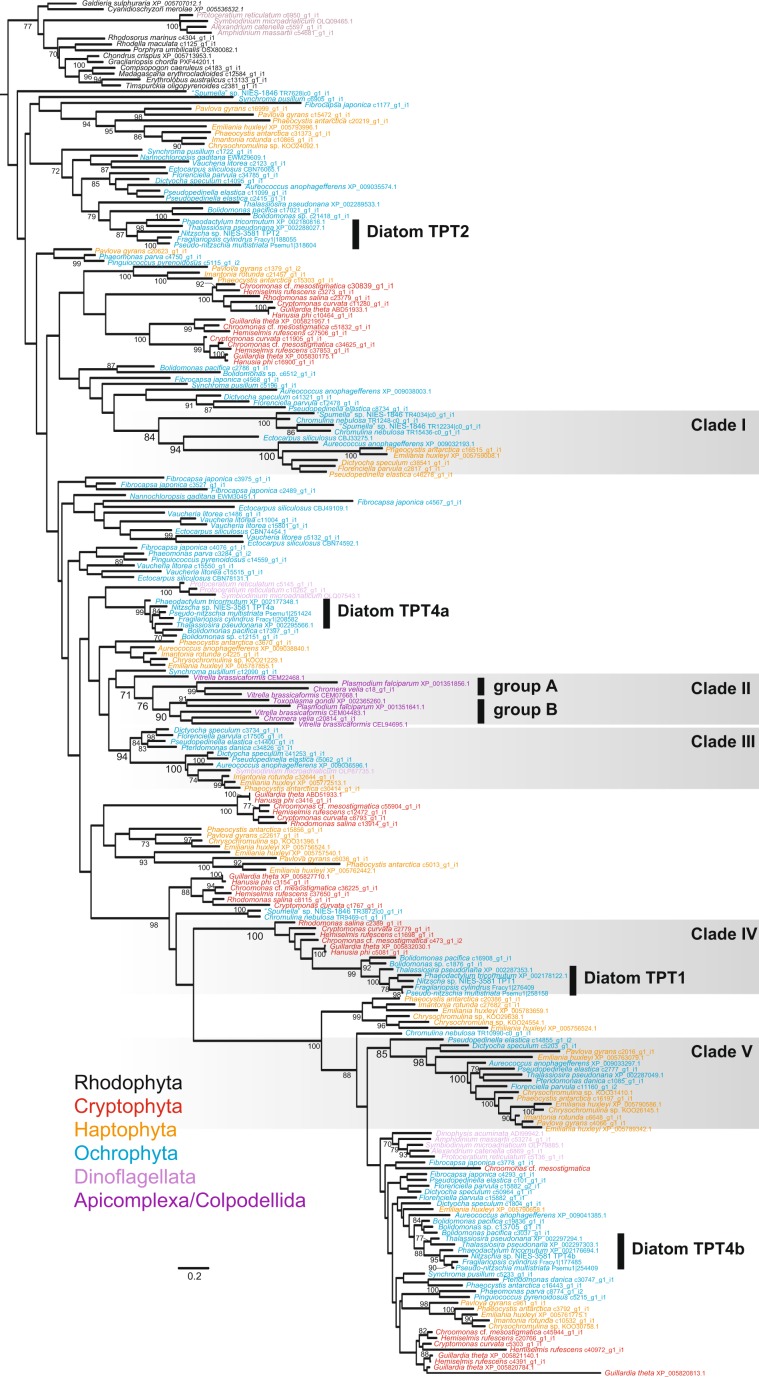
Phylogeny of TPT homologues in red algae and lineages with red alga-derived complex plastids. The maximum likelihood tree was inferred with IQtree under the LG + R8 + F model. Bootstrap values ≥ 70% are shown on branches. The remaining part of the tree including GPT, PPT, and XPT is shown in Supplementary Figure S2.

In the monophyletic clade of TPT homologues from algae with a red alga-derived complex plastid, we focus on five clades highlighted in Fig. 3. In clades I, III, and V, haptophyte sequences are closely related to homologues of Ochrophyta or a dinoflagellate, supported with moderate to highest bootstrap values (Fig. 3). Some TPT sequences of particular Ochrophyta lineages, Bacillariophyceae/Bolidophyceae, seem to be closely related to homologues of Cryptophyta, supported by 100% bootstrap value (clade IV in Fig. 3).

Another interesting monophyly can be observed in clade II, in which apicoplast TPT homologues are monophyletic with counterparts of photosynthetic species in Colpodellida, *Vitrella brassicaformis* and *Chromera velia*, closely related to Apicomplexa (Fig. 3). Clade II includes two groups, group A and group B, each supported by high bootstrap values (≥90%). In group A, the apicoplast TPT sequence of the apicomplexan *T. gondii* is grouped with one of two apicoplast TPT sequences of the apicomplexan *P. falciparum*. The apicomplexan group is then united with TPT homologues of the colpodellids. In group B, the other TPT homologue of *P. falciparum* is grouped with other copies of colpodellids.

### Amino acid residues responsible for TPT substrate specificity

Structural analyses of the pPT homologues unveiled a basis of substrate specificity^13^. The TPT of *A. thaliana* incapable of transporting PEP has His184, Lys203, Phe262, Tyr338, Lys359, and Arg360 at the six positions directly responsible for forming the substrate binding pocket, out of which Phe262 with a bulky, benzyl group sidechain is thought to be crucial to inhibit PEP access to the pocket^13^. In contrast, the apicoplast TPT homologue of the apicomplexan parasite *Toxoplasma gondii* is capable of transporting PEP in addition to triose phosphate^20^. The dual specificity for triose phosphate and PEP in the apicoplast TPT homologue was predicted to be caused by the replacement of Phe262 by Ser with a simpler, benzyl group-lacking sidechain^20^. Similar to the apicoplast TPT homologue, we found that diatom homologues of TPT1, TPT4a, and TPT4b had Ala, Ser, and Ser, respectively, all of which possess simple, benzyl group-lacking sidechains, at the homologous position of Phe262 of the *Arabidopsis* TPT (Supplementary Fig. S3). The other five amino acid residues, such as His184, Lys203, Tyr338, Lys359, and Arg360 of the *Arabidopsis* TPT, are highly conserved in all of the mentioned diatom TPTs (Supplementary Fig. S3). The diatom homologues of TPT2 commonly have the aromatic amino acid Phe at the corresponding position of Phe262 of the *Arabidopsis* TPT (Supplementary Fig. S3). Interestingly, two of the other amino acid residues responsible for forming substrate binding pocket, His184 and Tyr338, are replaced by Gln and Asn, respectively, in TPT2 (Supplementary Fig. S3). It is worth noting that His and Tyr have bulky, benzyl group sidechains while Gln and Asn have simpler side chains without the benzyl group.

It might give us further insight into the evolution and function of TPT homologues of red alga-derived complex plastids to more broadly compare the six amino acid residues involved in forming the substrate binding pocket. The amino acid residues characteristic for apicoplast TPTs which are able to transport PEP are also conserved in the TPT homologues of the colpodellids *V. brassicaformis* and *C. velia*, the photosynthetic species closely related to apicomplexans (Supplementary Fig. S3). Similarly, various TPT homologues of Ochrophyta, Haptophyta, Dinoflagellata, and Cryptophyta, including non-photosynthetic species such as *Dinophysis* (Dinoflagellata) and “*Spumella*” sp. (Crysophyceae, Ochrophyta^34^), possess amino acid residues, involved in forming the substrate binding pocket, identical with those in TPTs of diatom plastids and apicoplasts (Supplementary Fig. S3).

## Discussion

### Plastid-cytosol crosstalk with various sugar phosphates in diatoms

In this study, we demonstrate that NspTPT1, NspTPT2, and NspTPT4a integrated in the artificial liposomes are capable of transporting triose phosphates (TP), phosphoenolpyruvate (PEP), and 3-phosphoglycerate (3-PGA), but seem to be incapable of transporting glucose-6P (G6-P; Table 1). Similar substrate transport capabilities have also been reported for the TPT homologues of the malaria parasite *P. falciparum* and the cryptophyte *G. theta*^20,21^. As the three examined diatom TPTs localize in the outermost, the second outermost, and the innermost plastid membranes, respectively (Fig. 1), the equivalent substrate preferences of those transporters suggest that various sugar phosphates, except for G6-P, can be exchanged between the cytosol and the stroma of the non-photosynthetic plastid via active transport across membranes.

Import of TPs into the non-photosynthetic diatom plastid generates ATP and NADPH by conversion to glycerate 1,3-bisphosphate and 3-PGA catalyzed by glyceraldehyde-3P dehydrogenase and glycerate kinase^29^, as demonstrated in the red alga *G. sulphuraria* under heterotrophic growth conditions^17^. The expected energy flow in the diatom and the red alga is different to non-photosynthetic plastids of land plants which lack TPT but import G6-P and PEP by GPT and PPT, respectively^17,33^. Import of PEP and 3-PGA into the diatom plastid would contribute to ATP generation catalyzed by glycolytic enzymes including pyruvate kinase that converts PEP, ADP, and pyrophosphate to pyruvate and ATP^29^. Sugar phosphates are also prerequisite metabolites for biosynthesis of fatty acids, branched chain amino acids, and aromatic amino acids in plastids. Pyruvate, which is synthesized from PEP, is the primary substrate for biosynthesis of fatty acids and branched chain amino acids. Biosynthesis of aromatic amino acids requires supply with PEP and E4-P, the former which could be imported by the TPTs (Table 1) and the latter which could be synthesized from triose phosphates by the plastid non-oxidative pentose phosphate pathway in *Nitzschia* sp. NIES-3581^29^. Thus, the NspTPTs capable of transporting TPs, 3-PGA, and PEP might be able to fuel various metabolic pathways present in the non-photosynthetic diatom plastids.

G6-P in land plants serves as a substrate for starch synthesis, the oxidative pentose phosphate pathway, and fatty acid synthesis^35–37^. Inability of G6-P transport into diatom plastids is consistent with the absence of an oxidative phase of the pentose phosphate pathway and a lacking starch synthesis in the plastids of diatoms^25,38^. Instead, the fatty acid synthesis in diatom plastids can be fueled by PEP import facilitated by the TPTs (see above).

Although the diatom *P. tricornutum* contains a photosynthetic plastid whereas the plastid of *Nitzschia* sp. NIES-3581 is non-photosynthetic, our results show that the localization and the C-terminal orientation of TPT homologues are conserved in the photosynthetic diatom *P. tricornutum* and the non-photosynthetic *Nitzschia* sp. NIES-3581 (Fig. 1 ^18^). In addition, on the basis of comparison with the *Arabidopsis* TPT, which is incapable of transporting PEP, we found conserved residues responsible for forming the substrate binding pocket between homologues of the model photosynthetic diatom *P. tricornutum* and the non-photosynthetic *Nitzschia* sp. (Supplementary Fig. S3). As in *P. tricornutum*, homologues of TPT1, TPT2, TPT4a, and TPT4b are present in all of the other diatoms investigated in this study with the conserved amino acid residues forming the substrate binding pocket (Fig. 3 and Supplementary Fig. S3), suggesting that specificity for multiple substrates is also conserved in plastid TPT homologues of photosynthetic diatoms. These findings suggest a crosstalk mediated by TP, PEP, and 3-PGA between the plastid and the cytosol of various photosynthetic diatoms as in the non-photosynthetic diatom. It is highly likely that a certain amount of phosphorylated sugar compounds generated by the Calvin Benson cycle is exported into the cytosol in photosynthetic diatoms for fueling cytosolic and mitochondrial metabolisms as well as carbon storage^10,25,39^. Thus, the transport direction of phosphorylated substrates is likely opposite from that in the non-photosynthetic diatom. Because of i) the conserved localization of TPTs integrated in the diatom plastid membranes and ii) the putative opposite direction of sugar phosphate transport in photosynthetic and non-photosynthetic diatom plastids, we hypothesize that diatom plastid membranes are capable of transporting phosphate in counter exchange of multiple phosphorylated substrates bi-directionally. Such bidirectional transport of substrates across membranes in single TPT homologues was also predicted in the plastid of the mixotrophic red alga *G. sulphuraria*^17^.

### Possible complex evolution of TPTs in the red alga-derived complex plastids

The origin and evolution of red alga-derived complex plastids are still controversial. So far, the “chromalveolate” hypothesis has been the main hypothesis, that is, all the lineages with the red alga-derived complex plastids are monophyletic and their last common ancestor has engulfed and enslaved a red algal endosymbiont^40,41^. In this hypothesis, non-photosynthetic species closely related to a red alga-derived complex plastid-bearing lineage are regarded as the organisms that have lost such a plastid secondarily. However, given the phylogenetic position of Cryptophyta more closely related to Archaeplastida, which possesses a primary plastid, in recent phylogenomics [e.g.^42–44^], the “chromalveolate” hypothesis is currently strongly challenged. Recently, the current distribution of red alga-derived complex plastids was proposed to have been shaped by serial endosymbioses following the first secondary endosymbiosis between an ancestral red alga and a eukaryote  host^3,42^, although the exact evolutionary route of transfer of the red alga-derived complex plastid remains to be elucidated. Regardless of which hypothesis is true, genes for plastid TPT homologues in the first eukaryote bearing a red alga-derived complex plastid were most likely of endosymbiotic origin and have undergone EGT and duplication [e.g.^18^]. However, it is unclear how genes for plastid TPT homologues were acquired in the current lineages with a red alga-derived complex plastid, i.e., Ochrophyta, Haptophyta, Cryptophyta, Apicomplaxa, Colpodellida, and Dinoflagellata. Although most deeper branches are not well resolved in the tree (Fig. 3), we find close relationships between haptophytes and Ochrophyta organisms (clades I and V in Fig. 3). These organisms are not sisters to each other, on the basis of phylogenomics [e.g.^42,45^]), and thus several explanations could be invoked: analytical artifacts, lateral gene transfer (LGT), and/or EGT. If these phylogenetic relationships of TPT homologues are no artifact but derived from EGTs associated with the “serial endosymbioses” hypothesis, they might offer some clues for the evolutionary route of plastid transfers among lineages with red alga-derived complex plastids. Especially, the Haptophyta-Ochrophyta affinity of nuclear-encoded plastid protein sequences was discussed before^6,46^. However, plastid phylogenomics reconstruct Haptophyta and Cryptophyta as monophyletic excluding Ochrophyta [e.g.,^47^] and the Haptophyta-Cryptophyta monophyly is supported by an exclusively shared, non-cyanobacterial Rpl36 gene in the plastid genomes^48^, inconsistent with the above scenario^6,46^. Therefore, we cannot conclude whether the observed groupings are explained by artifacts, EGTs, or LGTs.

The other case of an interesting clade within the phylogeny of TPTs in organisms with complex red algal-derived plastids is that comprised of cryptophytes, diatoms and Bolidophyceae (clade IV in Fig. 3). Cryptophyta possess a remnant of the red alga-derived nucleus in the complex plastid (called nucleomorph), representing vivid evidence that the cryptophyte plastid stems from a red algal endosymbiont, and not from a diatom (as the phylogeny in Fig. 3 might suggest). The plastids of diatoms/bolidophytes in turn are of vertical inheritance from the last common ancestor of Ochrophyta [e.g.^49^]), which, according to^6^, could have gained the complex plastid from a cryptophyte endosymbiont. An explanation for the arrangement of the clade would be that only diatoms kept the cryptophyte-derived TPT gene(s) while other ochrophytes have lost it/them. Additionally, the cryptophyte-diatom monophyly observed in clade IV could also be explained by LGT between cryptophytes and diatoms.

It would be worth noting that dinoflagellates possess TPT homologues phylogenetically related to those of Chloroplastida (dinoflagellate clade I; Fig. S2). Another dinoflagellate clade is nested in the red algal TPT homologues (dinoflagellate clade II; Fig. S2). If these observed clade arrangements are not a result of LBA, but reflect the real phylogenetic situation of the factors, the origins of the respective dinoflagellate TPTs cannot be explained by EGT – neither in the light of the “serial endosymbioses” hypothesis nor the “Chromalveolata” hypothesis.

In addition to the above, recent gene duplication followed by relocation of a gene product into a different membrane would have also contributed to the evolution of TPT homologues in red alga-derived complex plastids (Fig. 3). It is known that the two apicoplast TPT homologues of *Plasmodium falciparum* localize in different apicoplast membranes, one (XP_001351856.1) in the innermost membrane and the other (XP_001351641.1) in the outermost membrane^22^. Each of these two homologues is grouped with TPT homologues of close photosynthetic relatives in clade II (Fig. 3), strongly suggesting that they are derived from a gene duplication followed by re-location of their products to different membranes, evolutionary events which likely have happened in the common ancestor of Apicomplexa and Colpodellida. The apicomplexan *T. gondii* has an outermost membrane-localized TPT homologue (XP_002365260.1) phylogenetically closely related to the outermost membrane-localized *P. falciparum* homologue, but lacks a counterpart of the *P. falciparum* innermost membrane-localized TPT homologue^23^, suggesting secondary loss of one TPT gene in *T. gondii*.

In summary our phylogenetic analysis gives no conclusive clue to understand the exact routes of evolutionary plastid transfers that have shaped the current distribution of red alga-derived complex plastids. Nevertheless, the TPT evolution in eukaryotes with a red alga-derived complex plastid appeared to be highly likely comprised of not only a simple EGT but also mixtures of other evolutionary events including putative LGTs, gene duplication, relocation of gene products to different membranes, and secondary loss.

### Contribution of TPT homologues to establishment of a controllable metabolic connection

As shown elsewhere, apicoplast TPTs were capable of transporting PEP in addition to triose phosphate and 3-PGA^20^. However, the origin of such substrate specificity remained unclear, given that plant and red algal TPT homologues both are incapable of transporting PEP^20^. Importantly, the apicoplast TPT homologues are, as discussed above, most likely derived from vertical inheritance from a photosynthetic ancestor. TPT homologues of *V. brassicaformis* and *C. velia*, close photosynthetic relatives of apicomplexans, have common amino acid residues at the positions responsible for forming the substrate binding pocket involved in substrate specificity (Supplementary Fig. S3). Therefore, the TPT homologues of *V. brassicaformis* and *C. velia* have the potential to form a substrate binding pocket in which PEP would fit and might be capable of transporting PEP in addition to TP and 3-PGA. If that conjecture is true, an ancestral TPT homologue of the common ancestor of Apicomplexa and Colpodellida could have possessed the PEP-transporting ability. Similarly, not all but many of the TPT homologues detected from ochrophytes, dinoflagellates, cryptophytes, and haptophytes share the same amino acid residues at the homologous positions responsible for substrate specificity with the apicoplast and diatom TPT homologs (Supplementary Fig. S3), suggesting that they might have the potential to transport PEP in addition to triose phosphates as well. This possibility can explain why we have not detected an apparent PPT homologue in any eukaryotes possessing red alga-derived complex plastids (Supplementary Fig. S2; see also^18,29^), in contrast to land plants and red algae^9^. Lack of PPT in those eukaryotes might be compensated by the TPT with extended substrate specificity in red alga-derived complex plastids. Possibly, birth of a TPT homologue capable of PEP transport could have played a role as the selective pressure to lose PPT homologues in the evolution of red alga-derived complex plastids. Given the absence of PPT homologues in all the red alga-derived complex plastid-bearing algae, the birth of a PEP-transportable TPT homologue followed by loss of PPT homologues might have happened early during the establishment of the red-algal endosymbiont as a photosynthetic organelle before the spread of red alga-derived complex plastids into various branches of the eukaryotic tree of life. If so, the TPT homologues might have played a key role in the establishment and retention of a controllable connection of metabolites between the host and the red algal endosymbiont during an early phase of the initial secondary endosymbiosis presumably without the involvement of other pPT homologues.

## Methods

### Localization of the C-terminal orientation of TPTs

For the self-assembling GFP assay, TPT-encoding sequences of *Nitzschia* sp. NIES-3581^29^ were cloned together with the small gfp fragment (GFP11) into the pPha_DUAL_2xNR (NCBI accession number: JN180664) vector, carrying a gene sequence encoding a marker protein of a complex plastid subcompartment fused with the large gfp fragment, or the gene encoding the GFP(S1–10) only, as described in^18^. After culturing positive clones in liquid f/2 medium containing 0.89 mM nitrate (NO_3_^−^) as sole nitrogen source for 6 h^31^, cells were screened for green fluorescence using a Leica TCS SP2 confocal laser scanning microscope with an HCX PL APO 40 × /1.25–0.75 Oil CS objective. Excitation of eGFP and chlorophyll fluorescence occurred at 488 nm with a 65 mW Argon laser, whereas fluorescence emission was detected at a bandwidth of 500–520 nm for eGFP and 625–720 nm for chlorophyll (plastid autofluorescence), respectively.

### Cell-free protein synthesis

The NspTPT1, NspTPT2, NspTPT4a, and NspTPT4b nucleotide sequences (see Supplementary Data) for cell-free translation were synthesized at Eurofins Genomics. The synthesized NspTPT2, NspTPT4a, and NspTPT4b nucleotide sequences were different from genuine ones with respect to that 1) codon usage was optimized for the cell free translation system that uses the extract of *Triticum aestivum* (wheat), 2) predicted signal peptide and transit peptide-like coding sequences were removed to avoid artificial conformational changes of the proteins due to presence of signal sequences not required for protein function, but 3) only the synthesized NspTPT1 retained the entire sequence containing a signal peptide region. Signal peptide prediction was performed by SignalP3.0^50^. After removing the predicted signal peptide region, the transit peptide-like sequence was predicted by ChloroP1.1^51^. We confirmed that the predicted cleavage site is upstream from a conserved mature protein region using BLASTp (https://blast.ncbi.nlm.nih.gov/Blast.cgi?PAGE=Proteins). The NspTPT1 has been synthesized as the entire sequence because the predicted signal peptide cleavage site is included in the conserved, possible mature protein region, a prediction which might be unreliable. The synthesized sequences were cloned into the pEU3b vector (Cellfree Sciences). The transcription of messenger RNA and wheat (*T. aestivum*) germ cell-free translation in the bilayer mode were accomplished as previously described^32,52,53^. Briefly, mRNAs transcribed from the pEU3b plastids carrying TPT genes were added to a 25-µL translation layer overlaid with a 125-µL substrate layer. To the translation layer, liposomes prepared from acetone-washed asolectin (Fluka, Buchs, Switzerland) were added at a final concentration of 10 mg/ml. Accudenz density gradient ultracentrifugation was done as described previously^32^.

### Reconstitution of the synthesized proteins into liposomes and measurement of transport activity

The complex of each synthesized TPT and liposome was washed twice in 10 mM Tricine-KOH (pH 7.6) by centrifugation at 20,000 *g* for 20 min, followed by resuspension of the pellet in 10 mM Tricine-KOH (pH 7.6). Substrate-preloaded liposomes (final concentration 30 mg/mL) were prepared from acetone-washed asolectin by ultrasonication for 5 min on ice in a solution containing 200 mM Tricine-KOH (pH 7.6), 40 mM potassium gluconate, and 60 mM of the appropriate counter-substrate (phosphate, DHAP, 3-PGA, PEP, or G6-P). The buffer-exchanged translation mixture was mixed with an equal volume of substrate-preloaded liposomes, frozen in liquid nitrogen, thawed at room temperature, and subjected to ultrasonication for 18 s (50% duty cycle). The substrate that remained outside of the resulting proteo-liposomes was removed with a Dowex AG-1 × 8 column (Bio-Rad, Tokyo) pre-equilibrated with 10 mM Tricine-KOH (pH 7.6), 50 mM potassium gluconate, and 100 mM sodium gluconate. The proteo-liposomes were applied to the column and eluted with the equilibration solution. Transport reactions were initiated when 10 µL of [^32^P] phosphate (PerkinElmer) was added to 200 µL of proteo-liposomes (final concentrations, 0.5 mM of [^32^P] phosphate outside and 30 mM of counter-substrate inside). The assay was performed at 25 °C, and the reaction was terminated when 15 µL of stop solution (360 mM pyridoxal 5´-phosphate and 64 mM mersalyl acid) was added to the reaction solution. The proteo-liposomes were passed through a Dowex AG-1 × 8 column pre-equilibrated with 150 mM sodium acetate to remove non-translocated radioactive [^32^P] phosphate, and the resulting proteo-liposomes were counted with a liquid scintillation spectrometer to evaluate the transport activity. The transport activity for the proteo-liposomes was determined by comparison to control liposomes prepared in parallel from translation reactions not supplied with mRNA. Those procedures were performed according to^32^.

### Phylogenetic analysis

We retrieved the protein sequences of pPT homologues from GenBank and the MMETSP data^54,55^. The protein sequences were aligned by MAFFT^56^ and ambiguously aligned sites were removed manually by BioEdit^57^. The resultant dataset comprised of 297 taxa and 274 sites (Supplementary Data) was subjected to IQtree v1.6.10^58^ for the maximum likelihood tree inference. The best-fitting available model based on the Bayesian Information Criterion was the LG + F + R8 model, which was used for estimation of the maximum likelihood tree and for a bootstrap analysis with the 100 pseudoreplicates.

The alignment used for the phylogenetic analysis was then used for identification of amino acid residues at the positions for formation of the substrate binding pocket, with the *Arabidopsis* TPT as a reference^13^.

## Supplementary information

Supplementary information.
