# Supplementary material for: Substrate specificity of plastid phosphate transporters in a non-photosynthetic diatom and its implication in evolution of red alga-derived complex plastids
Source: Sci Rep. 2020 Jan 24;10:1167. doi: 10.1038/s41598-020-58082-8 (PMC6981301; doi:10.1038/s41598-020-58082-8)
Supplement: Supplementary file 1 — Supplementary information. [file 41598_2020_58082_MOESM1_ESM.pdf]

Substrate specificity of plastid phosphate transporters in a non-photosynthetic diatom and its implication in evolution of red alga-derived complex plastids

Daniel Moog[1,2], Akira Nozawa[3], Yuzuru Tozawa[4], Ryoma Kamikawa[5,*]

[1]Laboratory for Cell Biology, Philipps University Marburg, Karl-von-Frisch-Str. 8, 35032 Marburg, Germany, [2]SYNMIKRO Research Center, Hans-Meerwein-Str. 6, 35032 Marburg, Germany, [3]Proteo-Science Center, Ehime University, 3 Bunkyo-cho, Matsuyama, Ehime, 790-8577, Japan, [4]Graduate School of Science and Engineering, Saitama University, Saitama 338-8570, Japan, [5]Graduate School of Human and Environmental Studies, Kyoto University, Kyoto 606-8501, Japan

*corresponding author: Ryoma Kamikawa (kamikawa.ryoma.7v@kyoto-u.ac.jp), Graduate School of Human and Environmental Studies, Kyoto University, Yoshida nihonmatsu cho, Sakyo ku, Kyoto, Kyoto 606-8501, Japan

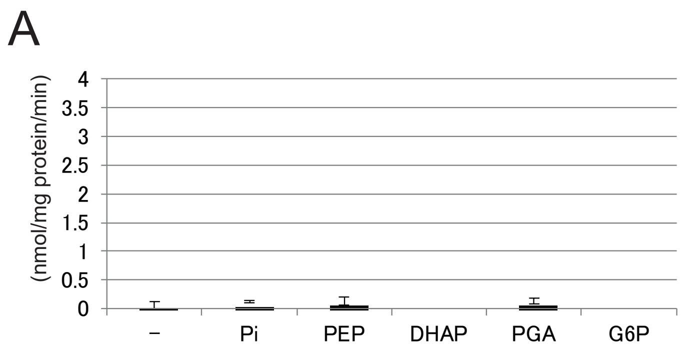

B

TPT4b
M S V V N V V R G G S T A S A P P A V A K P A A G G L D V G L **M** V Y F F L
ATGTCGGTAGTTAACGTCGTGAGAGGAGGCAGCACAGCAAGTGCCCCGCCTGCCGTCGCTAAGCCTGCTGCAGGCGGCCTGGATGTTGGTCTG**ATG**GTCTACTTCTTCCTG

M16
M S V V N V V R G G S T A S A P P A V A K P A A G G L D V G L **V** V Y F F L
ATGTCGGTAGTTAACGTCGTGAGAGGAGGCAGCACAGCAAGTGCCCCGCCTGCCGTCGCTAAGCCTGCTGCAGGCGGCCTGGATGTTGGTCTG**GTG**GTCTACTTCTTCCTG

M27
M S V V N V V R G G S T A S A P P A V A K P A A G G L D V G L **V** V Y F F L
ATGTCGGTAGTTAACGTCGTGAGAGGAGGCAGCACAGCAAGTGCCCCGCCTGCCGTCGCTAAGCCTGCTGCAGGCGGCCTGGATGTTGGTCTG**GTG**GTCTACTTCTTCCTG

TPT4b
W Y L G N Y Y Y N I T N K L A L N A S G G K T G F P **M** A V S G L Q L G V G
TGGTATCTCGGCAACTACTACTACAACATCACCAACAAGCTCGCGCTCAATGCCTCCGGCGGAAAGACCGGGTTTCCA**ATG**GCCGTGTCTGGGCTTCAACTCGGTGTTGGC

M16
W Y L G N Y Y Y N I T N K L A L N A S G G K T G F P **M** A V S G L Q L G V G
TGGTATCTCGGCAACTACTACTACAACATCACCAACAAGCTCGCGCTCAATGCCTCCGGCGGAAAGACCGGGTTTCCA**ATG**GCCGTGTCTGGGCTTCAACTCGGTGTTGGC

M27
W Y L G N Y Y Y N I T N K L A L N A S G G K T G F P **V** A V S G L Q L G V G
TGGTATCTCGGCAACTACTACTACAACATCACCAACAAGCTCGCGCTCAATGCCTCCGGCGGAAAGACCGGGTTTCCA**GTG**GCCGTGTCTGGGCTTCAACTCGGTGTTGGC

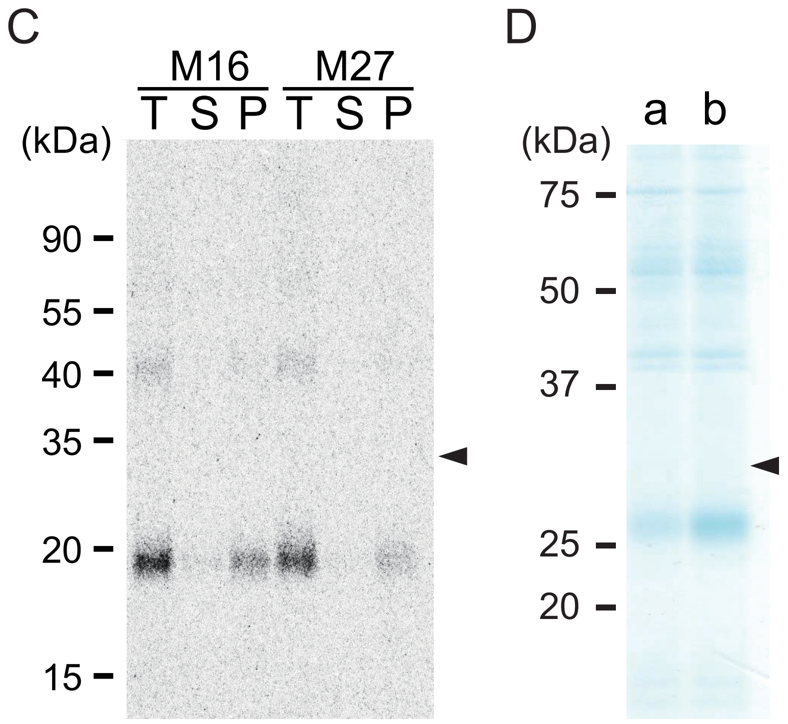

Supplementary Fig. S1. Analyses of truncated, synthesized TPT4b.

A. Transport activity of [32P] phosphate with different substrates. Error bars: standard deviation. B. Replaced methionine codons by valine codons. The replaced codons are highlighted in red. The second methionine codon was replaced by a valine codon in clone M16 while both second and third methionine codons were replaced by valine codons in clone M27. C. Synthesis of the TPT4b homologue of Nitzschia sp. NIES-3581 with valine codons replacing the second and third methionine codons, by a wheat cell-free system in the presence of [14C] Leu. T: Total fraction of the reaction mixtures, S: supernatant, and P: pellet. Each fraction was subjected to SDS–PAGE and autoradiography. D. Synthesis of the TPT4b homologue of Nitzschia sp. NIES-3581 at a low temperature. NspTPT4b was synthesized at 14°C by a wheat cell-free system. Total fractions of the reaction mixtures were subjected to SDS–PAGE. a: 50 μL applied, b: 100 μL applied. Arrowheads indicate expected molecular weights of NspTPT4b.

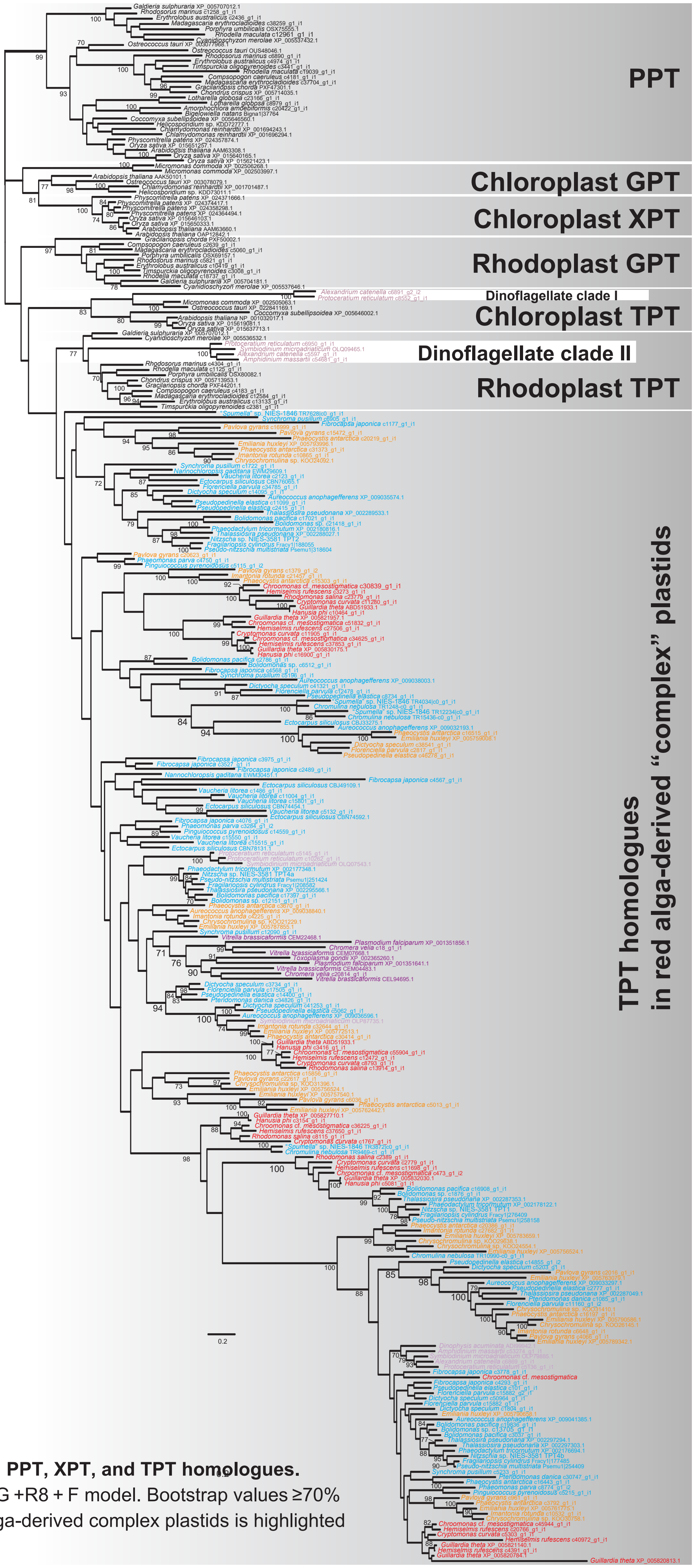

Supplementary Fig. S2. Phylogeny of GPT, PPT, XPT, and TPT homologues. The tree was inferred with IQtree under the LG +R8 + F model. Bootstrap values ≥70% are shown on branches. Each taxon of red alga-derived complex plastids is highlighted in colors as in Fig. 3.

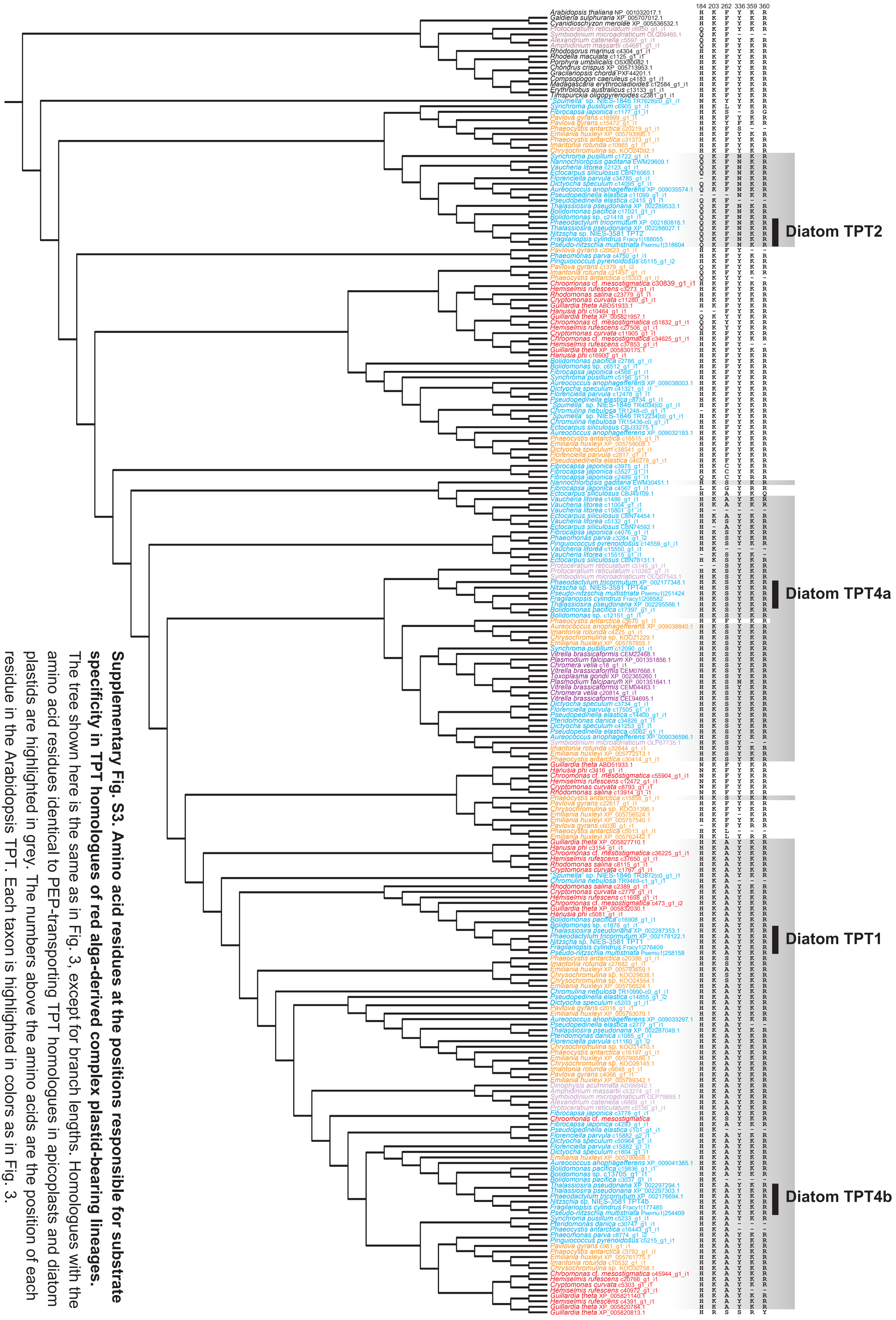

Supplementary Fig. S3. Amino acid residues at the positions responsible for substrate specificity in TPT homologues of red alga-derived complex plastid-bearing lineages. The tree shown here is the same as in Fig. 3, except for branch lengths. Homologues with the amino acid residues identical to PEP-transporting TPT homologues in apicoplasts and diatom plastids are highlighted in grey. The numbers above the amino acids are the position of each residue in the Arabidopsis TPT. Each taxon is highlighted in colors as in Fig. 3.

-: missing amino acid residues mainly due to truncated sequences.

Supplementary data. Nucleotide sequences used for synthesis of TPT proteins

>NspTPT1 of which codon usage is optimized to that of *Triticum aestivum*
ATGTCAGCCACCAAGGGCAAGGAGTCTGCCCCAGCCGGTGCCTCGCCAGCGAAG
CTTGCGCTACTGGTCGTGTGCTGGTATGCCGGGAATACCGCCTACAATATCTAC
AACAAGAAGGCTACCAAGATGATTCACGCGCATTGGTTCGTCGCAACTGCGCAA
CTCGTGGTTGGTATCGTTTGGAGCATGATAATGTGGGCCACAGGACTCCGCAA
GACACCCAACCTCACTGCGAAAGATATTGCCGACTGCATTCCGATCGGCCTGTT
TGCGTGCTTGGCCCATTGTGGCAGCGTGCTAGCTTCGGCAGTTGGAGCTGTTT
CCTTCGCACAGATCGTGAAGGCATGTGAGCCCGTGTTCGCAGCCGCGGTCGGA
TTGCTCATTCCTCCCATGGACGTGAAGCCGATACTCGCGTATGCGATGCTCGTC
CCGATTGTGGGCGGTGTTGGGATTGCTTGCGTCAAAGAGGGCAAAGGGGTTGA
CATCAATGTCGAGGCATTCTTGTTTGCGAGTATGGCCAATGCGGCCGCTGCTCT
GAAGGGGAAGCTTGGCTCATCTGTGACGAAAGCGCTCAAATCCGATCCAGCGAA
GAATATGGATGCCGCCAACGTGTACGCCGTCATGAACATCATCAGCTTTCTCTG
CTCCGTTCCGTTCGTGGTGGTGCAAGAGCTTCCAACGCTTAGCGGCGAGTGGA
ACAAGGCAGTCGAGGAACACGGTCTGCAAGACCTCATGTACAACATCATCGTAA
GCGGGTTCATGTTCTACATCTACAACGAGTTCGCCTTTGCGTTCACATCCCACG
TAGGCGCAGTGACCTCCTCAGTTCTGAACACGGCTAAGCGGGTCATAATCATCG
TAGCCGCCTCTGTCGTGTTCCAGGAAGCCATGGAAAGGAACACCGTCATAGGCA
GTGCTATCGCAATCACTGGAACCTTTGCCTATTCGCTGGCTTCCAAGAAGCCAG
CTCCTAAGGCCAAGACCGCATGA

>plastid-targeting sequence-lacking NspTPT2 of which codon usage is optimized to that of *Triticum aestivum*
ATGACCTCATCTACAGCCTTGCGTGGAGGCGATGACTCTTCCGAGGACTCGAGC
AATTCCCTCCGCGCCAGACTCGTTGTGGGGTTCTACTTCTTTGCATGGTATGCC
CTGAATGTGGTGTACAATATCGTGAACAAGAAGGTCCTTAACGTCCTTCCGGCA
CCACTTACCGTCGCTACTATCCAACTGGGCGTTGGCGCGGTCTACTCAGCCCTC
GTGTGGATGCTGCGGCTCAGGGCTTACCCCAAGCTCACTAGCTCAGGTGCCAAA
GCCGTCGGCTTCGTGGGACTGTACCACTGTCTAGGCCAACTCGCGACTGTTATG
TCGTTGGGCGCGGACCAGTCAGCTTTACCCACATAGTGAAGGCCCTCGAGCCG
TTCTTCTCTGCCGTGCTATCAGGGCTGTATTTCCAGAAGTGGATGAGGGGAGG
TGTTTACGCCACCTTGATCCCTGTCGTAGGAGGTGTGTCCTATGCGTGCCTTAA
GGAGCTGAACTTCAGCTGGGTGGCACTGTCCGCGGCAATGGCCTCGAACATCG
CATTCGCCTTGAGGGCCATTATGTCGAAGTTGGCCATGCAGTCTGGTGAGCAAG

CTGGGACCAATCTCACACCTCCGAACATGTTTGGCGTCGTCACGTGGGCAGCCT
TCTTCATTTCGCTGCCTCTGTGCGTATTCGGCGAACCACAATTTCTCACCCTTC
TCGGTGATGCGATGGATTCCGTCCCAGACAAAGGGCAGTTTATCCAGAGTCTCC
TTCTGAGCGGGCTCTTTCACTACCTCAACAACGAAGTGATGTACTTGGCTCTCG
GGAAGGTTCATCCAGTGACGCTCGCGGTCGGCAATACGATGAAGCGGGTTTTCA
TCCTCGTAGCGTCCGTTATGGTGTTCCAGAACCCAATCAGCCAGCAAGCCGCGA
TAGGCTCTACAGTGGGCATTGCCGGGGTCCTCCTATATAGCCTGACCAAGCAAT
ACTACGAGGCGCTTGACGCCAAAGAAGCGGCTGCGGCGGCTGCAACGGCAGCC
GCTAAGGCTGCCGAAGCAATCACATCCGTTCCCAAGATGATGTCCCTTCCCCAA
CTGAACCTCCCAGAGGAGCTAAAGAGCCTCCCCGAGAAATTGAACGTGGCTATC
CCTCATGACTGGTCAGAGCTGCAGGAGAAGCTAAACATCAGTATTCCGGAGAAG
ATAACCTGGACTTTGCCCGAAAACTGGAAGATGCCGGAGAAAATCACGTGGACT
CTGCCGCAGAATTGGAAACTCCCAGAGAAGATTACCTGGAGTCTCCCAGAAAAG
CTGCCCTTTCGCCGCAAGAAGCAAGAGTGA

> plastid-targeting sequence-lacking NspTPT4a of which codon usage is optimized to that of *Triticum aestivum*

ATGTCGGCGTCTACCAGGGTTGCACTATCGACCACAGGCGGAGAACAGCCACCA
GCTGCTGCGAAACCGAACCTCATGGCAACGTTGGAGGTAGGTTCGTATTTTGGC
CTCTGGTACCTCTTCAACATCGGCTACAACATCTACAACAAGCAAGCGCTCAATC
TCCTCGGCTATCCATGGACTGTTGCCACCCTCCAAATGGCTACTGGCATCGCCT
ACTTCGTCCCGCTATGGCTCCTTGGCTTGCGGAAAGCCCCTAAGCTCTCAGCCG
CAGACATAAAGACGCTCATACCAATCGCCCTGTGTCACACTGGTGTGCATGCAG
GAGCGGTAGTTGCCCTAGGTGCTGGAGCGGTGTCCTTTGCCCACATCGTGAAG
GCTAGCGAGCCGGTTGTTACGTGCGCCTTGAACTTCCTGTTGTTGGGCCAAGT
CCTCCCCATGCCAGTTTACCTGACACTGCTCCCGATTATCGGAGGGGTCGCGAT
TGCCTCGATGAAGGAGCTGTCCTTCACTTTTCTGGCCCTTGCGTCTGCTATGCT
CTCCAATGTGTCCTCAGCTGCGAGAGGAGTGCTGTCCAAGAAAACCATGAGTGG
GAAGAAGATTGGGGAGAACCTCGATGCGCAGAACCTTTATGCCGTGCTCACGGC
TATGTCTACCATCATGCTGATACCCATGACCTACGCCATTGAGGGGACTGGCAT
GTTCAAGGTGATCGGGGAACTGATCGCCAGCGGCCAACAGACCCAGAAGAGCAT
CACAACCTTGCTTGCACTTGGCGGTGCAAGCTACTACGCTTACAACGAGGTGGC
ATTCCTAGCACTTGGCAAGGTGAATCCCGTCACACATGCCGTCGGTAATACCAT
CAAACGCGTCGTCATCATTGTGGCCTCAGTGATCGCCTTCAAGACGCCTATGAG
CACAGGGTCAATTGTCGGGTCCAGTGTCGCGATAGCGGGCACCCTCCTGTATAG
CCTGGCGATGAATGCCTCTAAGAAGAAGAACTGA

> plastid-targeting sequence-lacking NspTPT4b of which codon usage is optimized to that of *Triticum aestivum*

ATGTCGGTAGTTAACGTCGTGAGAGGAGGCAGCACAGCAAGTGCCCCGCCTGC
CGTCGCTAAGCCTGCTGCAGGCGGCCTGGATGTTGGTCTGATGGTCTACTTCT
TCCTGTGGTATCTCGGCAACTACTACTACAACATCACCAACAAGCTCGCGCTCAA
TGCCTCCGGCGGAAAGACCGGGTTTCCAATGGCCGTGTCTGGGCTTCAACTCG
GTGTTGGCGCCCTCTATGGACTGTTCCTCTGGTTCGCTCCAGATGCACGCGAGA
AACCCAAGGTGACAATGCAGGACATCATCAAGATGATACCCGTTGCGTTCTGCT
TTATGGGAGCTCACTGTGCCTCCGTGTTCGCAATGGGGATGGGTGCTGTCAGC
TTTGCCCAAATCGTGAAAGCGGCAGAACCGGCGTTTGCGGCTGTGCTAGCGCAA
TTCGTGTACAACAAGCCAGTCTCAAGGGCTAAGTGGATGTGCCTACCGATTGTC
ATCGGAGGGGTGGTACTTGCGTCCGTGAAGGAGCTTGACTTCGCATGGTCGGC
CCTGATAAGCGCCTGCATTGCCAACACTTTTGCGGCCGTTAAGGGGAATGAGAA
CAAGAAGCTCATGGAAACTGAGGGACTAAAGGACCGTATGGGCTCTGTGGGGAA
CCAGTTCGCGCTCACGACCATCATGGCCTTCCTCTTTAGCCTGCCAGTGATCGC
CATGAAGGAGGGTTCGCGGATTGGCGAGTTCATGGAGTTGGTTAAGACCTCAC
CAGCAGTATGGCAGAATCTGGTCGCCTCAGGCCTTTGGTTCTACGGCTATAACG
AGGTTGCGACAATGACGCTGAAGAAAACTGGCGCCGTCACGCAAAGTGTCGCGA
ATACCGCTAAAAGGGTCATCGTCATAGTGGGTGTTGCCCTCGTGTTGGGCGAAT
CTCTTAGCCCCATCAAGTTGATTGGGTGCTCCATCGGTATTGGCGGGGTGCTCT
TGTACTCCGTCTAA

Supplementary data. The amino acid sequence dataset (297 taxa and 274 sites) used for phylogenetic analysis. Ambiguously aligned sites have been already removed. Each taxa name is comprised of the organismal name, strain number (if present), and either of the GenBank number, the JGI number, or the contig number.

```
>Spumella_sp._NIES-1846_TR7628
VWLRAGLYLVLWYGFTVAYNLSNKHLLTIFYPYTVAWLQLLIGWLIA---WPLIL-SAPN
FNHGAIALCHGLGNLASVMSFFLGSVSFAHVVKAAEPVIAAILSAVILRQRFPVIVYASL
LPIVLGVGLASATEFSFTWMGFWSAMASNVCYQARIVFAKREMLSAAQLFRVITVLSAIQ
LAPIALVMEGLRTVLWPATIRRHLLISGLTYYLYNEVAFWLLDLIHPMTHAVANTVKRVV
IILAAIVFLDSPVTVLGLVGSFVAVAGTLLYSSV
>Spumella_sp._NIES-1846_TR3872
ENTKLLVYLAVWYLGNVYYNIYNKKACIALLHWALSAAQLLVGALFVIPAWLTGLRKAPS
LELSPVGLWASLSHAFSVLALGAGAVSFGQIVKSAEPVFAAVTNAAILKDIDHPMVYAAL
IPIIGGVGLASLRELSFSWTALIAASLANQAAAFKNVVSKGVMLGPQNTYAVVNILALLF
TLPFVIAFDLFASVYEQVDVLKYSAYSGLAYYLYNEASFLALEKLSPVTHSVANTLKRVV
IIIASCIVFNTPMDTIGGIGSAIAVLGTLLYSLA
>Spumella_sp._NIES-1846_TR4034
ETLKMVGLFTLWYSFNSAYNVYNAYVKKDFFPWTTSAIQLGVGLLYAIPLWALGLRKRPN
LKILPISMLNAGGHACAVIAMGG---SFTHVIKASEPVVSVILGFLINGVVPKPLTALSL
LPITYGVAYASTLGKEFTTNAAKMAMGSNVAFALRSIFRKNL-LDPANDHAVATLLSFLL
TIPCALFFEDIKNTLLNIMFVFNAMVCGLSYYMYNELQNKVLGSLGAVPTAVGNTLKRVF
IFVALYFTSGETFPPAKVLGSAIAVLGCLAFAIF
>Spumella_sp._NIES-1846_TR12234
SAFYLFALFIVWYAFNAAYNVYNAYVKHDLLPVTVSTAQLAIGLLYAIPLWVLGIRKIPN
LTLLPIVVMNAAGHMAAVYAMGGGS--FTHVIKASEPVVSVILGVFLLGAIPKPLTALSL
LPITYGVAYASTLGNELTTNAAIFAMVSNVSFSLRSILRKNL-LNPANEHAITTFFSVLV
SLPLVYYLEGLMAGYEAITFALNLFLCGMAYYLYNEMQNIVLGSLGPVPTAVGNTLKRVV
IFVALYFTSGETFPMPKVVGCAIAIVGCFAFAIF
>Alexandrium_catenella_OF101_c6869_g1_i1
IDIVLLVYFALWYLGNYYYNITNKLALKASFPMTIATLQLGVGCLWALLLWGPDCRNLPK
IKTLLVGFTSAGAHAASVYALSAGAVSFGQIVKAAEPAFAALVGTLFYSTKVSKAKWLCL
IPVIGGVVLASLGEMNFAWAALFTAALANVFAAFKANENKKLMGNVGNQFALTMINSFLF
CLVLMLITEGFGQFLDLALCWHNLLYSGIWFYAYNELATLTIKRTNAVTQSVANTAKRVI
VIVGVAMVMGESLAPTKLFGCFIGIGGVFLYSVI
>Symbiodinium_microadriaticum_OLP79885.1
```

IDWVLLAYFALWYLGNYYYNITNKLALKAAFPMTIATLQLGVGCLWALLLWGPDTRKLPE
IKTLLVGLTSAGAHAASVFALSAGAVSFGQIVKAAEPAFAALIGTMFYSSKVSLGKWLCL
IPVIGGVVLASLAELDFAWAALITAAMANVFAAFKGNENKKLMGGVGNQFAITMINSFMF
CVVIMAITEGFGAFLKLAVVLLNLIFSGLWFYAYNELATFTIKKTNAVTSSVANTAKRVI
VIVGVALVMHESLSPLKLIGCSIGIGGVFLYSVI
>Protoceratium_reticulatum_CCCM-535---CCMP-1889_c5136_g1_i1
IDLVLLAYFALWYLGNYYYNITNKMALKAAFPMTIAAAQLFVGCIWGVFLWGPDCRSLPK
IKTLLVGFTSAGAHAASVFALSAGAVSFGQIVKAAEPAFAALVGTLFYSSKVSKAKWLCL
IPVIGGVIFASLGELNFAWSCLITASIANIFAAFKGNENKKLMGSVGNQFALTMLNSTIF
CGILAVFMEGFGEFVKLVVVSLNIIFSGLWFYVYNELATMTIKKTNAVTQSVANTAKRVI
VIVGVAIILGESLDPLKLFGCSIGIGGVFLYSII
>Amphidinium_massartii_CS-259_c53274_g1_i1
LDIPLILYFFFWYLGNYYYNITNKLALKAAFPMTIATLQLGVGCIYALFLWLPDARKTPS
IKTLPVGFTSAGAHAASVFALSAGAVSFGQIVKAAEPAFAALVGTAFYGATVSKAKWLCL
IPVIGGVVLASLAELDFAWAALITAGIANVFAAVKANENKKLMGSVGNQFAITTINSFLF
CSIAMFLTEGLGAFWKLIVVLMNMVYSGLWFYFYNELATLTIKKTGAVTQSVANTAKRAV
VILGVAVVLGESISTLKLVGSLVCIGGVFLYSVI
>Chroomonas_cf._mesostigmatica_CCMP1168_c45944_g1_i1
FDLQLIIYFGLWYLGNYYYNITNKMALKAAFPLTIATLQLGVGCIYALFLWAPDARKFPK
VAMIPVAFCAAAAHSFSVFALSAGAVSFGQIVKAAEPAFAAVLGVSLYGKKISKGKWLCL
VPVIGGVILASVKELDFAWSALFTASLANLFAAFKGNENAKLMENVGNQFAFTTILAFLL
SVPVMIIKEGWGQFCTLAIVMYNMIASGLWFYGYNELATMTIKKTSAVTQSVANTAKRVI
VIVGVAIVLGESLDPIKLLGCAIGIGGVFLYSII
>Guillardia_theta_CCMP2712_XP_005821140.1
IDFQLIAYFALWYLGNYYYNITNKLALKAAFPLTIATLQLGVGCLYALFLWAPDARKLPS
TKIVPVAFCSAAAHSFSVFALSAGAVSFGQIVKAAEPAFAALLGVTLYQKKVSKGKWLCL
IPVIGGVVLASVKELDFAWSALITACLANLFAAFKGQENQKLMGNVGNQFAITMILSFLI
SLPVMILKEGWGEFCTIWVVSFNLIASGLWFYGYNELATMTIKKTNAVTQSVANTAKRVI
VIIGVAIVLQESLDPIKLLGCAIGIGGVFLYSVI
>Hanusia_phi_CCMP325_c4391_g1_i1
IDFQLIAYFALWYLGNYYYNITNKLALKAAFPLTIATLQLGVGCLYALFLWAPDARKLPS
TKIVPVAFCSAAAHSFSVFALSAGAVSFGQIVKAAEPAFAALLGVTLYQKKVSKGKWLCL
IPVIGGVVLASVKELDFAWSALITACLANLFAAFKGQENQKLMGNVGNQFAITMILSFLL
SLPVMIVKEGWGEFCSIWIVSFNLIASGLWFYGYNELATMTIKKTNAVTQSVANTAKRVI
VIIGVAIVLKESLDPIKLLGCAIGIGGVFLYSVI
>Guillardia_theta_CCMP2712_XP_005820784.1

IDLQLIAYFALWYLGNYYYNITNKLALKAAFPLTIATLQLGVGCLYALFLWAPDARKLPK
IKMIPVAFCAAAAHSFSVFALSAGAVSFGQIVKAAEPAFAALLGVTLYQKKLSLGKWLCL
IPVIGGVVLASVKELDFAWSALITACIANLFAAFKGQENQKLMGNVGNQFAITMILSFLL
SVPVMIAKEGWGQFCSLWAVTYNLIASGLWFYGYNELATMTIKKTNAVTQSVANTAKRVI
VIIGVAIVLRESLDPIKLLGCAIGIGGVFLYSII
>Cryptomonas_curvata_CCAP979-52_c5303_g1_i1
VDFQLLIFFALWYLGNYYYNITNKLALNAAFPLTIATLQLGVGCIYALFLWAPDARRFPK
VKMIPVAFCSAAAHSFSVFALSAGAVSFGQIVKSAEPAFAALLGVTLYNKKVSLGKWLCL
IPVIGGVILASVKELDFAWSALITACLANLFAAFKGNENAKLMENVGNQFAFTTILSFLI
SVPVMLLKEGWPEFVALWAVSFNLLASGLWFYGYNELATMTIKKTSAVTQSVANTAKRVI
VIVGVAIVLKESLDPIKLLGCAIGIGGVFLYSVI
>Hemiselmis_rufescens_PCC563_c20766_g1_i1
IDFVLIIYFALWYLGNYYYNITNKLALKAAFPLTIATLQLGVGCIYALFLWAPDARSFPK
VAMVPVAFCAAAAHSFSVFALSAGAVSFGQIVKAAEPAFAAVLGVSLYNKKVSKGKWLCL
VPVIGGVILASVKELDFAWSALFTAALANLFAAFKANENAKLMENVGNQFAFTTILAFLI
SVPVMIAKEGWGSFCTLAIVMWNMIASGIWFYLYNELATMTIKKTSAVTQSVANTAKRVI
VIVGVAIVLGESLAPLKLIGCAIGIGGVFLYSVI
>Florenciella_parvula_CCMP2471_c15882_g1_i1
IDTGLAVYFFFWYLGNYYYNIVNKLALKAAFPMTISTLQLGVGCIYAIFMWLPDARSTPT
VKMIPVGFCAAGAHSSSVFALSAGAVSFGQIVKAAEPAFAALIGLVVYGKQISKAKWLCL
IPVIGGVCLASVKELDFAWSALIAAGIANLFAAFKGNENKKLMGSVGNQFALTTLISFIV
SLPFMYFKEGWGEFVTLFIVRNNLLLSGLYFYGYNELATMTIKKTSAVTQSVANTAKRVI
VIVGVAIVLGESLDPMKLLGCSIGIGGVFLYSVI
>Bolidomonas_pacifica_CCMP-1866_c19836_g1_i1
IDLPLMAYFFFWYVGNYYYNITNKLALKAAYPMTISSLQLGVGCLYALFMWVPDARSAPK
IKMLPVAFCSMGAHSASVFALSAGAVSFGQIVKSAEPAFAAVLSQFVYGKKISKAKWLCL
PIVIGGVILASVKELDFAWSALISACIANLFAAFKGNENKKLMGSVGNQFAVTSILSFLM
SVPILILKEGWGGFVELWIVSFNLIASGLYFYGYNELATMTIKKTSAVTASVANTAKRVI
VIVGVAIVLGESLDPIKLLGCSIGIGGVLLYSVI
>Thalassiosira_pseudonana_CCMP1335_XP_002297294.1
-----MLYFLFWYVGNYYYNITNKLALKAAFPMLISSLQLAVGSIYGIFLWLPDARDRPH
VKMLPVAFCFMGAHSASVFALSAGAVSFGQIVKAAEPAFAAVLSQFVYNKPVSSAKWACL
PIIIGGVILASVKELDFAWSALISACIANLFAAFKGNENKKLMGSVGNQFALTTILGFLM
SIPLVLLREGLGQFADLWILKTNLIASGLWFYGYNELATMTLKKTGAVTQSVANTAKRVI
VIVGVAIVLGESLDPIKLLGCGIGIGGVFLYSII
>Thalassiosira_pseudonana_CCMP1335_XP_002297303.1

IDVPLLTYFALWYLGNYYYNISNKLALKAAFPMTISALQLGIGSLYGIFLWLPDARARPH
VKMLPVAFCYAGAHSASVFSFASGSVSFGQIVKAAEPAFAAVLSQFVYNKPVSKAKWLCL
PIIIGGVILASANELDFAWSALISACIANLFAAVKGNENKKLMGSVGNQFCITSILGFLL
SIPFVLWKEGLGQFVDIWALRSNMIASALWFYGYNEVSTMTLKKTNAVTQSVANTAKRVI
VIVGVAIVLGESLDPLKLIGCGIGIGGVFLYSII
>Bolidomonas_sp._RCC2347_c13705_g1_i1
LDIPLLAYFFFWYVGNYYYNITNKLALKAAFPMTISSLQMGVGCLYALFMWVPDGRSKPS
VRMLPVAFCAMGAHCASVFALSAGAVSFGQIVKSAEPAFAAVLSQFVYGKKISKAKWLCL
PVVIGGVILASVKELDFAWSALISACIANLFAAFKGNENKKLMGTVGNQFAITSILAFLM
SVPLFLIKEGWGSFVEMYIVSFNLIASGLYFYGYNELATMTIKKTSAVTQSVANTAKRVI
VIVGVALVLGESLDPIKLLGCGIGIGGVFLYSII
>Aureococcus_anophagefferens_XP_009041385.1
LDFALLGYFFFWYLGNYYYNITNKLALKGSFPMTIASLQLGVGVVYALFAWVPDMRSIPA
LAMLPVAFCSMMAHCASVFALSAGAVSFGQIVKAAEPAFAAVLSQFVYGKPISQAKWLCL
IPVIGGVIIASVKELDFAVSALVAACSANLFAAFKGNENKKLMGSVGNQFAITSLLAFLM
SLPLMFATEGFGEFMEVLAVKSNFLLSGVYFYGYNELATMTIKKTNAITQSVANTAKRVI
IIIGVALVLGEDLPFVKLLGSAICIGGVFLYSVI
>Fragilariopsis_cylindrus_CCMP1102_OEU19149.1
LDLGLIAYFALWYLGNYYYNITNKLALNAAFPMTLSSLQLGVGAIYGIFLWLPDARTKPT
IKMIPVAFCFMGAHSASVFAMGMGAVSFAQIVKASEPAFSAVLSQFVYGKPISKAKWLCL
PIVIGGVILASVKELDFAWSALIAACIANCFAAVKGNENKKLMGSVGNQFAITTVLAFLL
SIPVVAIREGFGEFCEMAAIWKNLVASGLWFYGYNECATLTLKKTGAVTQSVANTAKRVI
VIVGVALVLGESLDPIKMLGCSIGIGGVLLYSVI
>Pseudo-nitzschia_multiseries_Psemu1|254409
LDLGLIAYFALWYLGNYYYNITNKLALNAAFPMTLSSLQLGVGAIYGLFLWLPDARTKPT
VKMLPVSFCFMGAHSASVFAMGMGAVSFAQIVKASEPAFSAVLSQFVYGKPISKAKWLCL
PIVIGGVILASVKELDFAWSALISACIANMFAAVKGNENKKLMGSVGNQFALTTILGFLL
SIPVVAIREGFGEFCEAAAIWKNLVASGLWFYGYNECATLTLKKTGAVTQSVANTAKRVI
VIVGVALVLGESLDPIKLLGCSIGIGGVLLYSII
>Emiliania_huxleyi_CCMP1516_XP_005790658.1
---------------------------------MTIGVLQLGVGVLYALYMWAPDSRSVPS
VKMLPVGFCAMGAHCFSVFALSAGAVSFGQIVKAAEPAFAAVVSTAVYGKSISTAKWLAL
IPVIGGVVLASVKELDFAWSALIAACTANLFAAFKANENKKLMGSVGNQFALTTILSFLF
GLPLLLAREGWSEFVSLFVISTNLFTSGLYFYLYNELATLTIKKTNAVTQSVANTAKRVI
VIVGVALVLGESLAPIKLLGCAIGIGGVLLYSLI
>Phaeodactylum_tricornutum_CCAP_1055/1_XP_002176694.1

VDIGLLLYFGLWYLGNYYYNITNKLALNAAFPMTISSLQLGVGSIYALFLWLPDARSRPK
IKMLPVALCFMGAHSASVFAMGMGAVSFAQIVKASEPAFAAVLSQFVYGKKVSTAKWLCL
PIVIGGVILASVKELDFAWSALIAACIANMFAAVRGNENKKLMGTVGNQFAITTVLGFIL
SLPVLFLREGFGEFVQLAAIWMNLVASGLWFYGYNECATMTLKKTGAVTQSVANTAKRVI
VIVGVALVLGESLSPIKLIGCSIGIGGVFLYSII
>Nitzschia_sp._NIES-3581_TPT4b
LDVGLMVYFFLWYLGNYYYNITNKLALNASFPMAVSGLQLGVGALYGLFLWFPDAREKPK
VKMIPVAFCFMGAHCASVFAMGMGAVSFAQIVKAAEPAFAAVLAQFVYNKPVSRAKWMCL
PIVIGGVVLASVKELDFAWSALISACIANTFAAVKGNENKKLMGSVGNQFALTTIMAFLF
SLPVIAMKEGIGEFMELVAVWQNLVASGLWFYGYNEVATMTLKKTGAVTQSVANTAKRVI
VIVGVALVLGESLSPIKLIGCSIGIGGVLLYSVI
>Dictyocha_speculum_CCMP1381_c1804_g1_i1
IDVPLILCFFFWYLGNYYYNITNKLALNAAYPMALSSLQLGVGVIYALFMWVPDARATPT
TAMLPVGFCAMGAHCASVFAMSAGAVSFGQIVKAAEPVFAAVLGTTVYGKQISKAKWGAL
IPVVGGVCLASVKELDFAWAALISACIANMFAAFKGNENKKLMGTVGNQFALTTLLSFAF
SIPLVLVREGLGELMEILAVWVNLLYSGLYFYGYNELATMTIKKTNAVTQSVANTAKRVI
VIVGVAIVMGESLDPMKLIGCGIGIGGVFLYSII
>Synchroma_pusillum_CCMP3072_c5233_g1_i1
TDWALLTYFALWYLGNYYYNITNKRCLNGAFALTISALQLGVGVIYALFLWAPDARKMPT
VRMLPVALCAAGAHSASVFALSAGAVSFGQIVKAAEPAFAAVLATTLYGKKVSKAKWLCL
IPVIGGVILASVKELDFAWAALISACIANLFAAFKGNENKKLMGSVGNQFALTTILAFII
SMPLAMLREGFGEFMTLFVVFNNLMLSGLFFYGYNELATMTIKKTGAVTQSVANTAKRVI
VIVGSAIVLGENLNGMKLLGCTIGIGGVFLYSII
>Fibrocapsa_japonica_CCMP1661_c4293_g1_i1
IDIPLTLYFAFWYLGNYYYNISNKLALNAAFPMTISCLQLLVGTVYAMFLWVPDARARPK
IKMLPVGVCAMGAHCGSVFAMGAGAVSFAQIVKAAEPAFAAVVGTLLYSSTISRAKWLSL
IPVIGGVTLASVKELDFAWSALFAAATANLFAAFKGQENKKLMGTVGNQFALTTIIAFLA
SVPLAFFREGWGQFVELFALKTNLIASGLWFYGYNELATMTLKKTNAVTQSVANTAKRVI
VIVGVALVMGESLDPLKMLGCSIGIGGVFLYSII
>Florenciella_parvula_CCMP2471_c15882_g2_i1
PDFALLTYFALWYLGNYYYNITNKLALKAAFPMIISTLQLGVGVIYALFLWAPDARNTPK
IKFLPVGLCAAGAHAGSVFALGAGAVSFAQIVKSAEPVFAAVIGTLVYKNSISLAKWLCL
IPVIGGVTLASVKELDFAWSALIAAAIANCFAAFKANENKKLMGTVGNQFALTTIFAFLF
SVPLAVWREGWAEFCTAWAIKLNMIMSGLWFYGYNELATMTLKKTGPVTQSVANTAKRVI
VIVGVAIVMGESLDPLKLLGCSIGIGGVFLYSII
>Dinophysis_acuminata_ADI99942.1

VDFGLFVVLALWYLGNYYYNITNKLALNAAFPMTIATLQFGVGALYAIFLWLPDARETPK
IKMGPVSIANTGAHAASVFALSAGSVSFAQIVKAAEPAFAAVIGTTVYKTKVSKAKWLAL
IPVIGGVCLASLGELNFAWAALITAGIANIFAAIKGNENKKLMGTVGNQFALTTITSFLF
ALPLMLIMEGLGEFFTLAAVLNNLVLSGLWFYSYNELATIVAKKTNAVTQSVANTAKRVI
VIVVVALVMGEGLSPLKLAGSTIGIAGVFLYSII
>Fibrocapsa_japonica_CCMP1661_c3778_g1_i1
VDFVLLGYFFFWYLGNYYYNITNKRALNAAFPISIAVLQLAVGAIYATFLWIPDARSKPA
INMLPVSFCAGGAHVFSVIALSAGAVSFGQIVKAAEPAFAALLGTLFYAKSISKAKWLSL
IPVIGGVVLASVKELDFAWAALFSALIANVFAAVKANENAKLMGTVGNQFALTTIIGFLM
CLPVMLLKEGMSEFIDLLAVWGNLLASGLWFYGYNELATMTIKKTSAVTQSVANTAKRVI
VIIGVAIVMRESLNPMKMLGCAIGIGGVFLYSII
>Dictyocha_speculum_CCMP1381_c50964_g1_i1
IDIALLAYFFFWYLGNYYYNITNKLALKAAFPMTISTLQLGVGVLYASFLWLPDARNRPA
IKFLPVGFCAAGAHAGSVFALGAGAVSFAQIVKAAEPVFAAVIGTLVYKSTYSMAKWLCL
VPVIGGVTLASVKELDFAWSALFSAATANVFAAFKGNENKKLMGSIGNQFALTTIFAFLF
SIPLMIWREGWGEFKQILIVKFNLIASGLWFYGYNELATMTLKKTSAVTQSVANTAKRVI
VIVGVAIVMGESLDPM------------------
>Hemiselmis_rufescens_PCC563_c40972_g1_i1
LDVQLIVYFFLWYAGNYYHNITGKIAFNMSFPVTIATMQMGIGCLYALFMWGPDARKFPN
ITMLPVAFCNAGVHGFSSFATSAGAISFGQMIKASEPAFAAVIGTTVYGKGVSTARWMCL
IPIIGGVSLASVTELNFAWLAFWAAGIANIFAAFKGQENAKLMETVGNQFALTTLASFLF
MLPVMVIKEGWPQFYSMLVWATNFVLSGLLLYGYNECATITIKKTSAITQSVANT-----

>Pavlova_gyrans_CCMP608_c961_g1_i1
IDVALMLYFFFWYLLNYYYTLNNKKALNAAFPMTVAFLQLIIGSLYGMFLWAPDARTFPK
ISMIPIAICFAGAHAASVFSMGAGAVSFTQIVKAAEPAFAAILGVTLYGKSVSKAKWLTL
IPVIGGVVLASVKELDFAWSALITAMTANLFAAFRSNENKKLMGTVGNQFALTMILSALV
LVPVFLATEAWGAFVETFALQVNLVTSGLWFYIYNELSTLTIKKTSATTQSVANTAKRVI
VIVGCALVLGESLDPIKMLGCAIGIGGVFLYSMV
>Imantonia_rotunda_RCC918_c10532_g1_i1
IDVQLLMYFAGWYLGNYYYTLNNKYALNAAFPVTIGFLQMLIGSVYALFLWSPDARPLPA
VKIVPVAACAAGAHISSIFSMNLGAVSFSQIVKASEPAFAALLGVTMYGKTISKAKWLCL
IPVIGGVCLASVKELDFSVAALVAACVANLFAAFRSNENKKLMGSTGNQFAISTIIGTAC
LGVFWLFTESFSTFMDMFELRNNLITSGLYFYLYNELSTLTIKKTSATTQSVANTAKRVI
VIVGVAIALGESLEFMKLLGCSIGIGGVLLYSLV
>Phaeocystis_antarctica_CCMP1374_c3792_g1_i1

IDFGLLAAFAGWYVGNYYYTLNNKLALKAAFPVTIGFMQLLIGSVYALFLWLPDCRPLPQ
VKVLPVAGCAAGAHLASIFSMNLGAVSFAQIVKASEPAFAAVVGTTLYGKSISQAKWLCL
IPVIGGVCLASIKELDFSVWALLAACIANVFAAFRSNENKKLMGSVGNQFALTTLLGALC
VLPVFLLTEGLGEFMELFILKSNLLTSGLYFYLYNELSTLTIKKTSATTQSVANTAKRVI
VIVGVAIALGESLEPVKMLGCGIGIAGVLAYSLV
>Emiliania_huxleyi_CCMP1516_XP_005761775.1
IDWTLMLYFAGWYVGNYYYTLNNKLALKASFPVTIGFLQMVIGSLYALFAWVPDMRPQPN
VQILPVAACAAGAHLSSIFSMNLGAVSFAQIVKAAEPAFAAFLGVTLYGKSISKAKWLCL
IPVIGGVCLASLKELDFSVWALLAACVANLFAAFRSNENKKLMGSTGNQFALSTILGTLS
ILPFWLLLEAFGTFVELFALSSNLVTSGLYFYLYNELSTLTIKKTSATTQSVANTAKRVI
VIVGVALALGESLEPIKMLGCGIGIGGVLLYSLV
>Chrysochromulina_sp._KOO30758.1
IDVGLLLCFAGWYLGNYYYTLNNKYALNAAFPITIGFLQMCIGSLYALFLWLPDARPLPT
VKIVPVATCSAGAHLSSIISMNLGAVSFSQIVKAAEPAFAALLGVTMYGKSISKAKWLCL
IPVIGGVCLASIKELDFSVVALIAACVANVFAAFRANENKKVMGSTGNQFAISTLLGALS
LLPVWLMTEAFGKFLEIFVLRNNLITSGLYFYLYNELSTITIKKTSATTQSVANTAKRVI
VIVGVALALGESLEPIKLLGCSIGIGGVLLYSLV
>Pteridomonas_danica_c30747_g1_i1
-----------W----------NKLCLNAAFPMTISTMQLGVGFLYAS-LWWLKLRSFPQ
IKMLPVSFAIMGTHSASVFAFSLGAVSFAQIVKAAEPVFAAFISIIIHGNKISFSKSIML
LPIIGGVILASCKELDFSWNALISASIANIFAAIKGNENKKLMNGISNQFTISTLIAFLL
SLPIMIYKEG--

>Chromulina_nebulosa_UTEXLB2642_TR10990-c0_g1_i1
VDFALLTYFAFWYLGNYYYNIQNKNAAIAAYAMTLATAQLGVGVIYALFLWVPDARKPPS
IKMIPAGFCSAGAHAASVFSLAAGGVAFGQIVKASEPAFAAVIGTFVYKKKISIYKWLCL
IPIIGGVILAALKELDFTIGGLVGALVANAFAAFKGNESKKLVGGVGNQFAIMTIISFLV
SVPLLFFKEGFPEFIELFELYTNVIYSGLTFYAYNELATKTLTKISAVTNSVANTAKRVV
VIVGSAIVFKESISGLKAIGCAICIGGVFLDSVI
>Phaeomonas_parva_CCMP2877_c8774_g1_i2
ADFLLLSYFAFWYLGNYYYNLTNKIALKAAFPALISTAQLGVGVLYSAFLWLPDARKVPR
LSMLPVGFCMAFAHGASVLALSAGTVSFGQIVKACEPAFAAVIGVSLYSKTVSTARWVAL
IPVILGVVIASVSELSFSWVALISACTANTFAAFKANENKRLMGSVGNQFAVTTLLSFLI
SVPFALAKEGIGEFIELAVIMNNMLSSGLLFYLYNEMATLTIKKTGAVTASVANTAKRVI
VIIGVAIALNEPLSTPKLVGSAICIAGVLLYSNA
>Pinguiococcus_pyrenoidosus_CCMP2078_c5215_g1_i1

VDYVLLLYISFWYLGNYFYNITNKMALKAAFPALIATAQLGVGAIYSLYLWAPDARAAPT
VKMIPVAFCMAAAHGASVFSLGAGSVSFAQIVKSCEPAFAAVIGALLYNKAVSKAKWLSL
IPVIGGVAIASATELNFSLAALFGACIANCFAAFKGNENKKLMGTVGNQFAVTNLLAFLI
STPIALAKEGFPEFLELCVVWNNMLASGILFYLYNELATMTIKKTSAVTQSVANTAKRAI
VILGVAIALGESLSPTKIVGSVICILGVLLYSNA
>Pseudopedinella_elastica_c14855_g1_i2
IDWGLLSLFAFWFLGNYYYNIWNKQCLKASFPLTISVMQLGVGVVYACLCWCPFIRKLPT
TKMFPVAFTNLGAHSASVFAMGAGAVSFAQIVKASEPAFAAVLSVLFYGKKVSLGKWLML
PIIIGGVILASVKELDFAVSALVAACTANAFAAVKGNENKKLMGGVSNQFALTTTISFLM
GLPLMFAQEGFGDFVELVDVRNNLVLSGLSFYGYNELATMTIKSTGAVTASVANTAKRVI
VMVYMAAVTGKALTEEQKIGAGVAICGVLLYSLI
>Chroomonas_cf._mesostigmatica_CCMP1168_c4479_g1_i1
LDIEMVLLFALWYVGNYYHNVSNKLALMAVFPITIATLQLAIGSAYAVFMWIPDGRPRPT
TSLMPLSFFAACAHVFSVFALSAGALSFGQIVKAAEPAFAAVLGTLFYRKRVSLGRWLCL
VPVIGGAVIASVAEPSFGWACLAAASTANVFSSLRANENERVMGSVGNQFAFTTVFSFLF
LLPLAVFAEGWGMFMEMCGVVVNLLASGLWFYAYNEAATSTIKKTNAITQSVANTAKRVI
LIVLAAAVLGESLHPVKMLGCSIGIAGVFAYSVI
>Imantonia_rotunda_RCC918_c27682_g1_i1
VDVGLIVYLTAWYAGNYYYNIYNKLAAKAAYAMTLATIQLIVGSLYALFLWVPEARKKPT
IQLAPLGFATAAAHAGAVFAMSAGAVSFGQIVKAAEPVFAAGIGYVFYNSTQSKAKLMCL
VPIVGGIWIASVEELDFTIAALLAASTANVASAFRGGENKKAQGGSGNMYALSTLWATIM
LVPLVFLSEYFGDFKKLWTLRWNTLLSGLTFYGYNEVSTLALQSISGVTHSVANTAKRAI
IIVGCAIAFGESMAPTKMAGCSIAICGTFLYAII
>Phaeocystis_antarctica_CCMP1374_c20386_g1_i1
MDIGLIVYLALWYLGNYYYNIYNKLSAKAAYAFTLAWLQLVVGSVYAIFLWAPEARAKPN
CKLAPLGFWAATAHAGAVFAMSAGAVSFGQIVKAAEPAFAVVVGYFVYSKVESKAKVAML
IPIIGGIMIASAQELDFTVAALLAGCTANVASAFRGQENKRVLGSAANVYAITTLWATVF
LFPVIFVSEYMDKFRAMWDLTFNTLMAGLTFYLYNEVSTLALKKISGVTHSVANTAKRAI
IIVGCAIAFGESMAPLKMIGCSVAIGGTFFYAII
>Chrysochromulina_sp._KOO29638.1
MDIALLVYLALWYLGNYYYNIYNKTAAMASFAFTNAFVQLVVGSIYAIFLWAPDARTPPK
LAVAPLGLWAAMAHAGAVYAMTAGAVSFGQIVKAGEPVFAVVVGFVFYSQKVSLAKILCL
IPVIGGIAIASAKELDFTMMSLLAASTANVASAFRGSENKKVMGSVGNAYALTTIWATVL
LFPVIFISEFFDQWLAIWNFRYNIIMSGLTFYLYNEVSTMALKSLSGVSHSVANTAKRAV
VIVGSAIAFGEDMGFAKSLGCSIAIGGTFLYAVA
>Chrysochromulina_sp._KOO24554.1

PSASLITYLTMWYVGNYWYNIYNKNAGKLSFAFTNATMQLVIGCAYAIFLWLPDARKQPT
LSLAPLGFFAAVAHGGAVYAMSAGAVSFGQIVKAGEPVFAAAVGYFVYKKSESVPKLICL
LPVIGGIAIASMQELDFTMASLIAASTANVASAFRGSENKRVMGSGGNSYALTTIWATIL
LTPTVFISEFIDEYLALLTFRYNLLMSGVTFYLYNEVSTKCLDGLSGVSHSVANTAKRAV
VIVGSALAFGEDMGFLKSVGCTIAIAGTFLYAIA
>Emiliania_huxleyi_CCMP1516_XP_005783659.1
--------------------------------MIMAWIQMAVGAVYALALWIPEARKAPA
IKLAPVGFFTAAAHAGAVFSLSAGAVSFAQVIKAAEPAFAAAIGYAVYGSSVSRAKLLML
VPVIGGICIASASELDFTWACLAAAGGANVAAAFRGQENKKAMGGGANAYAIGTLWSTLL
LIPTVFITEWMDAFLKVWGFRFNLIMSGLTFYLYNEVSTLALGKISGVTHSVANTAKRAI
IIVGCAIAFGESMSPSKMAGCSIAIGGTFLYAVA
>Emiliania_huxleyi_CCMP1516_XP_005756524.1
MEAAVVVYLLLWYVGNYYFNIFNKAAGIASFAFTLASLQLVIGSGWVVSLWVLGLRQRPA
LRLLPLGLVTAVAHGSAIYANLAGSLSFSQIVKAGEPAFAAAVGYGVYRNGVSWRKLLCL
VPVIGGIAIASATELDYTLASFAAVSLANLAAAFRGCENKRVMGGEGNAYGVTTLWAALL
LLPAIFATEYRSEFVALWGLRFNAVLSGLLFYLYNEVSTRALRHLSGVGHSVANTAKRAV
VIVGSAVAFGEQMDRPKAVGCAIAIAGTFLYAIA
>Imantonia_rotunda_RCC918_c6648_g1_i1
DSVALLLFFLFWYVGNAFYNQYNTLALGAALTMTVSTMQLGVCSLYAVVLWLPEKMPFPK
TKTIPVGFCSAAAHSAGVFCLG-ADPLFGQIVKAGEPVLSALVNTVFYGKPPSKAKFVCL
FFIVAGVAFASLKALKFDERALMFGMIGNAFAAFKGSENKKLMGGVANQFALTEVLAFFI
SVPVMFATEGWGKFVELLDLQIGLAVSGMSFYLYNELATMTIKATGAVTSSVANTAKRVI
VMVYMAAVTGKALTEEQKIGAGVAIGFVLVYSVI
>Pavlova_gyrans_CCMP608_c4066_g1_i1
DSVALLLFFLFWYVGNAFYNQYNTQALTAVLTMTVSTMQLGVCTLYAMLLWIPEKMALPK
IATLPVGFCSAAAHSAGVFCLG-ADPLFGQIVKAGEPVMSAGVNTVFYGKAPSKAKFVCL
LFIVAGVAFASLKALKFDERALLFGMIGNTFAAFKGSENKKLMAGVANQFAVTEVLAFLI
SLPVMFATEGFGEFIKLMDLQIGLAVSGMSFYIYNELATMTIKATGAVTSSVANTAKRVV
VMVYMAAVTGKVLTEEQKIGAAVAIGFVLVYSVI
>Emiliania_huxleyi_CCMP1516_XP_005789342.1
DSVALLLFFVFWYVGNAFYNQYNTLAIGATLTMTVSTMQLGVCTLYAMLLWLAESMPFPA
VSTLPVAFCSAAAHSAGVFCLG-ADPLFGQIVKAGEPVLSALVNTVFYKKRPSMPKILCL
FFIVGGVAFASLKKLKFDETALLFGMIGNTFAAFKGAETKKLMGGVANQFAVTECLAFAI
SLPVMFATEGWGTFMHLLDLQVGLFVSGLSFYLYNELATMTIKATGAVTSSVANTAKRVI
VMVYMAAVTGKVLTEEQKIGAAVAIGFVLVYSVI
>Emiliania_huxleyi_CCMP1516_XP_005790586.1

-DVSLLLYFVFWYVGNAIYNMYNTMALKAVLTMTVSTLQLGICSIYAGMLWLPEKMLLPK
TATLPVGFCAAAAHSAGVFCLG-ADPLFGQIVKAGEPVMSAFVNTFFYGKPPSLAKVFCL
FFIVAGVGFASLKKLKFDERALIFGMIGNSFAAFKGSENKKLMGGVANQFALTELIGFFI
SVPVMVATEWFPEFFRLLELQVGLVLSGMSFYLYNELATMTIKKTGAVTASVANTAKRVI
VLIFMSAVTGKALSDEQKIGATIAIAFVMLYSVI
>Chrysochromulina_sp._KOO26145.1
FDISLMLYFLFWYVGNAVYNMYNTMALKAVLTMTVSTLQLGVCSLYAALLWLPEKMPLPQ
TDTLPVGFCAAAAHSAGVFCLG-ADPLFGQIVKAGEPVLSAFVNTVFYGKPPSFAKVVCL
FFIVAGVGFASLKNLKFDERALIFGMIGNCFAAFKGSENKKLMGGVANQFALTEVLGFLI
SLPVMLATEWLPKFCKLLDLQIGLVVSGMAFYLYNELATMTIKKTGAVTASVANTAKRVF
VLVFMSAVTGKKLTTEQKIGAAIAIAFVMLYSVI
>Phaeocystis_antarctica_CCMP1374_c16197_g1_i1
DSVALLLFFVFWYVGNIYYNEYNKMALDGVLTMTVSTMQLGVCSVYALLMWIPEAMKLPK
LKTLPVGFCSAVAHSAGVFCLG-ADPLFGQIVKAGEPVFSAIVNTLFYGKPPSMAKAFML
LPIVGGVAFASLKALKFDETALIFGMIGNIFAAFKGSENKKLMGGVANQFALTEVLAFLI
SVPVMFMVEGWPKFVELVNLQIGLAVSGMTFYWYNELATMTIKATGPVTSSVANTAKRVI

>Chrysochromulina_sp._KOO31410.1
DSVALFLFFVFWYVGNIYYNEYNKMALDGALTMTVSTMQLGVCTIYALLMWLPDRMKLPE
TAMIPVGFCSAAAHSSGVFCLG-ADPLFGQIVKAGEPVLSAFVNTVFYKKSLSLAKVSCL
FCIVGGVAFASLKGLKFDQTALVFGMLSNAFAAFKGSENKKLMGDVANQFAMTEVLAFLI
SVPVMIATEWFPEFMHLLKLQVGLFVSGLTFYLYNELATMTIKATGPVTASVANTAKRVI
VMVYMSAVTGKALTEEQKIGSGIAIGFVLIYSVI
>Florenciella_parvula_CCMP2471_c11160_g1_i2
PTYSLLLFFFFWYAGNVKYNEYNKAALDSVMTMTVSTMQLGVCAVYALLIWIPDKQAFPK
SKTLPVGFCSAAAHSSSVFALG-GDPLFGQIVKAGEPVISAIVNTIFYGKPPTMLKALCL
PIIVGGVAFASLKALKFDQTALLFGMMANGFAAFKGSENKKLMGGVGNQFAVTEVLAFIW
SLPVMFAVEGFSEFVNLFACMFNVTASGLTFYLYNELATMTVKAVGPVGSSVANTAKRVI
VMLYMAAVTGKALTEEQQIGAAVAIGGVMLYSVI
>Aureococcus anophagefferens_XP_009033297.1
------------YAGNTKYNEYNKGALDAVMTMTVSTMQLGVCAAYAIVLWVPDRQKLPG
TKTIPVGFCAAAAHSASVFALGGGDPLFGQIVKAGEPVLSAIVNTIFYGKPPSLPKWCCL
PIIVGGVAFASMKTLKFDMTALQFGLLANAFAAFKGSENKKLMGGVGNQYAVTEILAFLI
SLPVMFYTEGWPKFLELLELQFNLAMSGLAFYLYNELATMTIKTTGAVTASVANTAKRVI
VLIYMAAITGKALTDEQKIGAGVAIGGVLIYSVI
>Thalassiosira_pseudonana_CCMP1335_XP_002287049.1

DSVALALFFVFWYAGNMKYNEYNTAALNAVLTMTVATMQLGVCALYALIVWTPEKQDVPK
VKSLALGFCSAGAHAATVFALG-GDPLFGQIVKSAEPVLAALIGTVFYNKAPTFNKVMCL
PVIVGGVAFASLKSLKFDTTALVFGMLANSFAAFKGGENSKLMGGVGNQFAVTQILGFFI
LLPIMFYTEGFPDFVNMLNLQFNLIMSGLCFYIYNELATYTLKVTGAVTASVANTAKRVI
VMVYMAAVTGKALTDEQKMGSAVAISGVLLYSLI
>Pteridomonas_danica_c1085_g1_i1
DNIALLLFFVFWYIGNAKYNEYNTGALESVMTMSISTLQLGVCALYSIILWIPSRQLIPA
IMTIPVGVFLSAAHSSSVFAFS-GDPIFGQIVKSGEPVLSALVGFLFYKKAPTFTKLLCL
PVIVGGVAFASLKQLKFDQTALIFGMLANIFAAFKGNENSKLMGGVGNQFAVTTIIAFIV
SVPLMFYTEGWPTLKQHLELQFNLVASGLGFYIYNELATMTISKTSAVTSSVANTAKRVI
VMIYMAAVTGKVLTEEQKIGAGVAIFGVFGYSVI
>Pseudopedinella_elastica_c2777_g1_i1
DSVALILFFVFWYAGNMKYNEYNTGALNAAYTMTISTMQLGVCAVYAILMWLPAAQKLPK
LATVPVGFCSAAAHSASVFALG-GDPLFGQIVKAGEPVLAAAVGLGVYGKTISMTKTLCL
PIIVGGVAFASLKGLKFDQTALLFGMMANAFAAIKGQENSKLMAGVGNQFAVTEIIAFCV
SLPVMFATEGFMPFCKLFALQWNLVMSGLSFYIYNELATM--------------------

>Dictyocha_speculum_CCMP1381_c5203_g1_i1
SMLSLVLYFAFWYLGNYYYNIYNKTALNETYAMTVSTMQLAVCTVYALTLWLPSKQSPPD
LKMGVVAFCSAGAHSASVFALNAGSVTFGQIVKSGEPVFAAVVNTIFYGKPPSLLKTLCL
PIIVGGVGFACMKSLDFDIAALTAGCIANGMAAFKGSENKKLMGGVGNQFAVTEILAFFI
SLPIMFYMEGFGEFVKLFKLQFNLVMSGLTFYWYNELATMTIKKTGALTSSVANTAKRVI
VIVGVAVAMKKPLSYEEKVGAAVAVAGVLLYSVI
>Emiliania_huxleyi_CCMP1516_XP_005763079.1
DKLALVLLYVFWFVGNYYYNLYNKQASMKALTVTISVMQIVVCAAWAMGLWLPAPQPLPA
ISLLPLTFCYAFAHTAGVVALTAGSPAFGQIVKAAEPVFAAVINTLFYAKSPSLAKWCVL
PVIVGGVAISTLKSVPLDLYVLAFGSVNNVFAAFKGSENHRAMGGVGNQFALTNVLSLVF
LVPCMVLSEGWPKFVDLFAFRWNLCLSGITFYLYNELATMTIKATGAVTASVANTAKRAI
VIVGMAIALGNPLKLEEKVGASTAIAGVFIYSVI
>Pavlova_gyrans_CCMP608_c2016_g1_i1
NNLKLLTLFALWYLGNYYYNIYNKVALNEAFAYTVATYQLLVAAMWAVLSWIMQSQKLPQ
LSVSYLAMWSAFAHLGSVLCMNAGSVAFGQIVKAAEPVFAAGVNTVAYSKPPTIAKALML
PVIIVGVAIACLKKVEFEVVAVLAGSFANLSAAFKGAENARVMGSVGNQFALSQVFGFVI
LVPVALVMEGLFEFFTLCTFLYNATMSGITFYGYNELSTMTITHTSAVTASVANTAKRVI
VIVGVAIAMGKPLTKEEMMGSAVAITGVFLYSVA
>Phaeocystis_antarctica_CCMP1374_c16443_g1_i1

IDLQLFSYFAFWYLLNYFYSINNKLCLNAAFPLTISTMQLGVGVLYSIFAWAPEMRTVPS
VSMIPVSFCAAAAHSFSVFAQSAGAVSFAMIVKAAEPAFAAIVGTLFYGKTISSTKWAML
VPVIGGVILASVKELDFAWAALITASLANLFAAFKGNENKKLM-----------------

>Bolidomonas_pacifica_CCMP-1866_c3037_g1_i1
MDFPLLAYFLFWYVGNYYYNITNKLALKAAFPMTISSLQMGVGCLYGLFMWVPDARTRPK
IAMLPVAFCAMGSHSASVFALSAGAVSFAQIVKSAEPAFAAVLSQFVYGRKISKAKWACL
PIVIGGVILASVKELDFAWS--

>Pseudopedinella_elastica_c101_g1_i1
IDVQLLIYFALWYLGNYYYNITNKLALNAAFPMILSCLQLGVGVIYATFLWAPDARPTPK
IKMIPVGVCAAGAHAGSVFALGAGAVSFAQIVKSAEPVFAAVIGTLLYGAKVSTAKWLAL
IPVIGGVTLASVKELDF---

>Guillardia_theta_CCMP2712_XP_005820813.1
VDSKMFPYFGLWYFGNCYFIITSKLALNAAFPVAIATLQLGFGCLYAFFLWASGSKTVPN
IKMLPVAFYAALAHSLFVYSIGAGAVSLSLLVRAAEPVFADFLAAATDKKKMSNAKILSL
LPIIGGIYFACNQQSDFAWTAVIAACMSNFFSVYKDYNQNKLVKSVGNQFELTMLLSFFL
SIPMMISAEGWDAFGVLLIILLNIIASGLWLYGSNLVANRYIKDPPPVVNSLLHAGRYAF
VMVGGALALAESIGPAQLVTYAVGLGGVFLYSLM

>Chromulina_nebulosa_UTEXLB2642_TR9469-c1_g1_i1
EQTKLSFYLIVWYLGNVYYNIYNKKACIALLHWALSAVQLLVGALFVIPLWLTGARPQPK
LELSPVGLWASLAHAFSVLALGAGAVSFGQIVKSAEPVFAAATNAVLLKDIDHPVVYLAL
LPIIGGVSLASLKELSFSWTALIAASLANQAAAFKNVVSKGVMLGPQNTYAVINILALVF
TLPFVLGFD---

>Bolidomonas_pacifica_CCMP-1866_c16908_g1_i1
STLKLVLLVMAWYAGNTLYNIYNKKATNAIAHWFVACAQNGIGIIWSLFMWATGIRKMPN
LTCLPIGLCAAVAHGGSVLAMGVGAVSFAQIVKACEPVFAAVIGLVVPVDVKPAIAYLML
VIIVSGVGLACVKEGDINMTALMFASLANLAAGFKGKLGAGAVMDAANVYAVMNILSFLW
TVPVVIVEELLSVEWEKAELITNIALSAFFFYVYNEFAFGFTSLVGAVTSSVLNTAKRVI
IIVVSAVIFSEGLGRNKMMGSAIAITGTFLYSLT

>Bolidomonas_sp._RCC2347_c1876_g1_i1

STLKLILLVTCWYAGNTLYNIYNKKATNTIAHWFVAAAQLALGIVWSLFLWATGLRKTPN
LACVPIGLCAAAAHGGSVLAMGAGAVSFAQIVKACEPVFAAVIGLLVPIDIKPPLAYAML
LVIVGGVGLACVKEGTINMTALMFASVANIAASFKGKLGHAVTMDSANVYAVMNILSFAW
TVPMVLINELLQEEWEGAAVAGNIALSGFFFYIYNEFAFAFTSQVGAVTSSVLNTAKRVI
IIVASAIIFQEGMGRNKMIGSAIAITGTFLYSLA
>Phaeodactylum_tricornutum_CCAP_1055/1_XP_002178122.1
KRLLAFVLVVCWYAGNTFYNIYNKKAANMIAHWFLAAAQLVVGIVWSLVMWGTGLRKTPN
LACIPIGLCASLAHSGSVLASAVGAVSFAQIVKACEPVFAAVVGILIPADIKPPLAYIML
AVIVGGVGLACVKEGDINVEAFLFASMANLAAALKGKLGSSVTMDAANVYAVMNIISFIC
TVPFVVFTELLRQEWDHANLLFNIGVSGFCFYIYNEFAFAFTANVGAVTSSVLNTAKRVI
IIVASSIVFQEVMERNTIIGSAIAIGGTFAYSLA
>Thalassiosira_pseudonana_CCMP1335_XP_002287353.1
SNLKLVGLVVAWYAGNTLYNVYNKKATNMIAHWFVACAQLVVGIIWSCVMWGTGMRKVPN
LACVPIGLMACLSHAGSVLAMGVGAVSFAQIVKACEPVFAAVVGLLLPMDIKPILAYAML
VPIVGGVGIACIKEGDINWTAFMWASIANLAAALKGKLGGSVTMDSANVYAVMNIISFLF
TVPMVLVAEMLPEEWDKAAVITNIALSGFFFYIYNEFAFAFTSNVGAVTSSVLNTAKRVI
IIVVSSIVFVEPMERNTVIGSAIAIGGTFAYSMA
>Nitzschia_sp._NIES-3581_TPT1
SPAKLALLVVCWYAGNTAYNIYNKKATKMIAHWFVATAQLVVGIVWSMIMWATGLRKTPN
LDCIPIGLFACLAHCGSVLASAVGAVSFAQIVKACEPVFAAAVGLLIPMDVKPILAYAML
VPIVGGVGIACVKEGDINVEAFLFASMANAAAALKGKLGSSVTMDAANVYAVMNIISFLC
SVPFVVVQELLSGEWNKADLMYNIIVSGFMFYIYNEFAFAFTSHVGAVTSSVLNTAKRVI
IIVAASVVFQEAMERNTVIGSAIAITGTFAYSLA
>Fragilariopsis_cylindrus_CCMP1102_OEU13792.1
SAAKLAFLVVAWYAGNTFYNVFNKKATNMIAHWFVACAQLVVGIVWSVFMWGTGLRKMPN
LSCIPIGMCACLAHAGSVLASAVGAVSFAQIVKACEPAFAAVIGLLIPMDVKPLLAYAML
IPIVGGVGLACVKEGDINVQAFAYASMANVAAALKGKLGSGVTMDSANVYAVMNIISFCC
TVPFVVVNELLDVEWNKAPLLINILMSGFFFYIYNEFAFAFTAAVGAVTSSVLNTAKRVI
IIVVSTIVFQEPMERNTVLGSAIAIGGTFGYSLA
>Pseudo-nitzschia_multiseries_Psemu1|318604
SPAKLAFLVVAWYAGNTFYNVYNKKATNMIAHWFVACAQLVVGIIWSAVMWGTGMRKKPN
LSCIPIGMCACLAHAGSVLASAVGAVSFAQIVKACEPAFAAVVGLLIPMDIKPLLAYAML
IPIVGGVGLACVKEGDINVAAFMWASLANIAAALKGKLGSGVTMDSANVYAVMNIISFLC
TVPFVVVQELLDVEWNKAPLLTNIILSGFFFYIYNEFAFAFTAAVGAVTSSVLNTAKRVI
IIVVSTIVFQEPMERNTVLGSAIAIGGTFGYSLA
>Chroomonas_cf._mesostigmatica_CCMP1168_c473_g1_i2

ETVELAVLFGAWYWGNIYYNIYNKKALNQIAHWTVAFAQLVVGVIWCLPIWATGIRKFPN
LSLAPIGLFAAASHGGSVLALGAGAVSFAQIVKACEPVFAALVALAVPIETKPALAYLML
LVIVGGVGLACVKEGEINMYAFGWASFANLAAALKGKMGKDITMTAPNVYAVMNIISAAW
TLIVVAFTELIQDTWDHTDIITNIFLSGVCFYLYNEFAFAFTAMVGPVTSSVLNTLKRVI
IIVVTAIVFGEAMDKNAMIGSGVAILGTMLYSLA
>Hemiselmis_rufescens_PCC563_c11698_g1_i1
ESVTLLFYVAVWYVCNTFYNIYNKKALNQIAHWTVAFTQLVVGVVWCMVIYGLQIRKFPK
LALAPIGLFAAASHGGSVLALGAGSVSFAQIVKACEPVFAAIIALAIPQEIKPALAYMML
LVIVGGVGLACVKEGEINMYAFGWASFANLAAALKGKLGKDVTMDAANVYAVMNVLSAAW
TLIVVLATEAIQGTWDQTDIIINVVASGVFFYLYNEFAFAFTAKVGPVTSSVLNTLKRVI
IIVVTAIVFGEIMDRNAMIGSGVAIAGTMFYSLA
>Guillardia_theta_CCMP2712_XP_005832030.1
NTTKLVLLVAGWYIGNTLYNIYNKKACNNIAHWSVAFAQLVVGVIWCAMLWIPGIRKAPN
LSLAPIGLFAAAAHGGSVLAMGAGAVSFAQIVKACEPVFAALIGIVVPIETKPALAYMML
LVIVGGVGLACVKEGEINVFAFGWASFANLAAALKGKLGKDQTMDAANTYAVMNILSALW
TFIAVASTELIQDTWNHADIILNITLSGVFFYLYNELAFAFTAEVGAVTSSVLNTLKRVI
IIVVTAIIFGEAMDRNAMIGSAVAIAGTMFYSLA
>Hanusia_phi_CCMP325_c5081_g1_i1
NTTKLVLLVAGWYIGNTLYNIYNKKACNNIAHWSVAFAQLVVGVIWCALLWIPGIRKAPN
LSLAPIGLFAAAAHGGSVLAMGAGAVSFAQIVKACEPVFAALIGIVVPIETKPALAYMML
LVIVGGVGLACVKEGEINVFAFGWASFANFAAALKGKLGKDQTMDAANTYAVMNILSALW
TFIAVASTELIQDTWNHADIIMNITLSGVFFYLYNELAFAFTAEVGAVTSSVLNTLKRVI
IIVVTAIIFGEAMDRNAMIGSAVAIAGTMFYSLA
>Cryptomonas_curvata_CCAP979-52_c2779_g1_i1
ESVSLTLYVVAWYVGNTYYNIYNKKALNLIAHWSIAFLQLVVGVLFIVPLWITGIRKAPK
LSLAPIGLFAAGAHGGSVLALGGGSVTFAQIVKACEPVFAAVVALAVPIETKPFLAYMML
LVIVGGVGLACVKEGEINMFAFGWASFANLMAALKGKLGKDITMDSANVYAVMNIISAVW
TLIPVIYEESIQHEWDHADIIFNTIASGIAFYLYNEFAFAFTAKVGPVTSSVLNTLKRVI
IIVVTSVVLGEVMERNAMIGSAVAIAGTLFYSLA
>Rhodomonas_salina_CCMP1319_c2389_g1_i1
ETLTMVMYVIAWYAGNTYYNIYNKKALNLLAHWTVAFAQLVVGILWCIPLWLTGIRSAPK
LKLAPIGLFAACAHGGSVLAMGAGAVSFAQVVKACEPVFAALVGLVIAVEVKSAIEYLML
LVIVGGVGLACVKEGDINYFAFGWASFANLAAGLKGKLGKDVSMSAANSYAVMNILSAIW
TLFVVLGTEAFQDTWTHTDIIFNVVMSGVAFYLYNEFAFAFTAMVGAVTSSVLNTLKRVI
IIVATAAIFGEVMDKNAMIGSGIAITGVLLYSLA
>Chroomonas_cf._mesostigmatica_CCMP1168_c36225_g1_i1

-------------------NIYNKKALNLVLVWSVATAQLIVGALWVIPLWLVGLRKKPE
MEMAPIGLWAAGAHGGSVVALGAGAVSFGQILKACEPAFSAVNEVLLTGNVQAWQVYACL
IPIIGGVAFASLKELSFSWLAVISAMIANQSAALKGVMGKTVMMGAANQYGVVNILSVLW
CIPVVLGMEGWKASWDKADIMLNVFLSGFAFYLYNEVSFMALAKVSPVTHSVANTLKRVV
IIVVSCIVFNTPMSTEGMVGSGIAITGTLLYSLA
>Hemiselmis_rufescens_PCC563_c37650_g1_i1
ETTEIAVLFALWYWGNIYYNIYNKKALNLVLVWSVATAQLAIGALWVIPLWLLGIRKSPN
MAMAPIGLWSAGAHGGSVVALGAGAVSFGQILKACEPAFSAVTEVILTGNVQAWQVYATL
IPIIGGVAFASLKELSFSWLAVISAMIANQSAALKGVQGKAVMMGAANQYGCANILSVLF
CIPVVLGLEAWKASWDRAEILTNTFLSGFAFYLYNEVSFMALAKVSPITHSVANTLKRVV
IIVVSCVVFNTKMTTEGIVGSAIAILGTLLYSLA
>Rhodomonas_salina_CCMP1319_c8115_g1_i1
ETVELAILFGLWYWGNTAYNVYNKKALNLLLVWSVAAAQLMTGVLWVVPLWILGLRTAPK
MQMAPIGIFAAGAHGGSVVALGAGAVSFGQILKACEPAFSAVNEIIFLGEVQAWQVYMTL
IPIIGGVAFASLKELSFSWLAVISAMLANQSAALKAVFGKSVMMGPANQYGVVNIISVLA
TLPLVLGLEAMQKSWDTADIYQNVLASGFFFYLYNEVSFMALAKVSPITHSVANTLKRVV
IIVVSCIIFNTPMSTEGMVGSAIAILGTLLYSLA
>Guillardia_theta_CCMP2712_XP_005827710.1
ETVELAIYFALWYWGNTYYNIYNKKAMNLLLVWTVSSAQLFVGILWVIPLWILGIRTSPK
MQMAPIGLWAAGAHGGSVISLGAAAVSFAQILKACEPVFSAANEAILLGKVQAWPVYAAL
LPIIGGVALASVKELSFSWLSVISAMIANQCAALKGVQGKDIMMGPANQYGVVNMLAFLW
TLPIVFAVEGAMESWENADVLKNVVFSGLTFYLYNEVSFLCLGKVTPITHSVANTLKRVV
VLVVSCIVFNTPVSRESIIGSTIAILGTLLYSLA
>Hanusia_phi_CCMP325_c3154_g1_i1
ETVELTVYFALWYWGNTYYNIYNKKAMNLLLVWTVSSAQLFVGILWVIPLWILGIRTSPK
MQMAPIGLWAAGAHGGSVISLGAAAVSFAQILKACEPVFSAANEALLLGKVQAWPVYAAL
LPIIGGVALASVKELSFSWLSVISAMIANQCAALKGVQGKDIMMGPANQYGVVNMLAFLW
TLPIVFAVEGAMDSWNNADVLKNVVFSGLTFYLYNEVSFLCLGKVTPITHSVANTLKRVV
VLVVSCIVFNTPVSRESIIGSTIAILGTLLYSLA
>Cryptomonas_curvata_CCAP979-52_c1767_g1_i1
ESVELGVLFGLWYFGNVQYNLSNKKALNLLLVWTVSMIQLFVGVLWVLPLWALGIRTAPK
LEIVPVGIFSAGAHGGSVLGLGAGAVSFAQILKACEPIFSAVNEIIFLGDIQAWQVYATL
IPIVGGVAYASAGELSFSWLAVIACMIANQCAALKAVFGKGVMLGAANQYGIVNIASWLT
LLPIALIVEGAQQSYNKADILWNVALSGIMFYLYNEVSFMALAKVSAVTHSVANTLKRVV
IIVASCIFLGEKMTTESVVGSTIAITGTLLYSLA
>Bolidomonas_pacifica_CCMP-1866_c17397_g1_i1

ETLTVTSYFMLWYIFNIGYNIYNKQALNVLYPWTIATIQMASGIPYFLTLWLTGLRLAPK
LKLIPIALCHTGVHVGAVIALGAGAVSFAHIVKASEPVATCVLNGIILKEVLPLPVYLTL
IPIIGGVAIASMKELSFTFLALASAMLSNISSAARGVLSKKTMMTAQNLYAVLTGMSTLI
LIPLMLLIEGIPAFKSLASLAGLLAAGGLTYYAYNEVAFLALGKVNPVTHAVGNTIKRVV
IIVASVIAFKTPMSRGSIVGSSVAILGTLMYSLA
>Bolidomonas_sp._RCC2347_c12151_g1_i1
---------MLWYLFNIGYNIYNKQALNVLYPWTIATIQMAVGALWFVPLWVTGARAAPK
LTLFPIALCHTGVHVGAVIALGAGAVSFAHIVKASEPVVTCVLNAVLLKEVLPLPVYGTL
LPIIGGVAIASMKELSFTFLALGAAMLSNLSSAARGVLSKKTMMDAQNLYAVLTAMSTCI
LVPLMLAMEGGPAFRSLVSLATLLLLGGGSYYAYNEVAFLALGKVNPVTHAVGNTIKRVV
IIVASVVAFKTPMSRGSVVGSTIAIAGTLMYSLA
>Thalassiosira_pseudonana_CCMP1335_XP_002295566.1
DTLKTASYFALWYLFNIGYNIYNKQALNALFPWTIATIQMATGILYFAPLWALGLRKAPK
LTLFPIALCHTGVHVGAVVALGAGAVSFAHIVKASEPVVTCAANALLLGETLPLKVYATL
LPIIGGVGIASMKELSFTYLALAAAMLSNVSSSLRGVLSKKTMLDAQNLYAVLTAMSTLI
LIPMMLAAEGIPAFKAAVSLSTLLLLGGATYYLYNEVAFLALGRVNPVTHAVGNTIKRVV
IIVASVIAFKTPMSTGSIVGSSIAIFGTLLYSLA
>Phaeodactylum_tricornutum_CCAP_1055/1_XP_002177348.1
ETLQVGSYFALWYLFNIAYNIYNKQALNVLYPWTVATIQMAAGLAYFVPLWVLGIRKAPK
LTLLPIALCHTGVHVGAVIALGAGAVSFAHIVKASEPVVTCALNALLLGQILPLPVYATL
LPIIGGVAIASLKELSFTWLALGSAMLSNVSSAARGVLSKKTMLDAQNLYAVLTAMSTLI
LIPAMLAMEGFSAFSQVVSLAMLIGLSGASYYAYNEVAFLALGKVNPVTHAVGNTIKRVV
IIVASVIAFKTPMSTGSIVGSSIAIAGTLLYSLA
>Pseudo-nitzschia_multiseries_Psemu1|251424
ETLQIGTYFALWYLFNIAYNIYNKQALNILYPWTIATIQMATGIFYFAPMWILGLRKAPK
LTLFPIALCHTGVHVGAVIALGAGAVSFAHIVKASEPVVTCLMNYLFLGQVLPLPVYATL
LPIIGGVAIASMKELSFTFLALASAMLSNVSSSARGVLSKKTMLDAQNLYAVLTAMSTLV
LVPAMLAIEGFTGFKDIVSLAALLALGGASYYAYNEVAFLALGKVNPVTHAVGNTIKRVV
IIVASVVAFKTPMSTGSIIGSSIAIAGTLLYSLA
>Fragilariopsis_cylindrus_CCMP1102_OEU16951.1
ETLQVGTYFALWYLFNIAYNIYNKQALNVLYPWTIATLQMCTGCLYFGPMWLLGLRKAPK
LTLIPIALCHTGVHVGAVIALGAGAVSFAHIVKASEPVVTCLLNYLFLGQVLPLPVYLTL
LPIIGGVAIASMKELSFTVLALASAMLSNVSSSARGVLSKKTMMDAQNLYAVLTAMASCI
LIPLCLAIEGFTAAKSVVGLAMLLGLGGISYYAYNEVAFLALGKVNPVTHAVGNTIKRVV
IIVASVVAFKTPMSTGSIIGSSIAIAGTLMYSLA
>Nitzschia_sp._NIES-3581_TPT4a

ATLEVGSYFGLWYLFNIGYNIYNKQALNLLYPWTVATLQMATGIAYFVPLWLLGLRKAPK
LTLIPIALCHTGVHAGAVVALGAGAVSFAHIVKASEPVVTCALNFLLLGQVLPMPVYLTL
LPIIGGVAIASMKELSFTFLALASAMLSNVSSAARGVLSKKTMLDAQNLYAVLTAMSTIM
LIPMTYAIEGFKVIGELISITTLLALGGASYYAYNEVAFLALGKVNPVTHAVGNTIKRVV
IIVASVIAFKTPMSTGSIVGSSVAIAGTLLYSLA
>Dictyocha_speculum_CCMP1381_c3734_g1_i1
ETLKVGGYFALWYILNIGYNIYNKKALNIVLPWTIATLQLFAGIPYVFFLWTTGLRKKPI
LNLAPSAICHLGTHVGAVLSLGAGAVSFTHIVKASEPVVSALLSAVFLKEFLPIPVYLSL
LPVIGGVGLASLKELSFSWLAFSTAMLSNVASASRAILSKTVLLTAQNLYAVLTILAFVM
LLPMSLCVETIKAVVDAAELAMVSALSGVFYYLYNEVAFLALNAVAPVTHAVGNTIKRVV
IILASVIVFRNPMTNLGAAGSAIAIFGTLLYSLA
>Florenciella_parvula_CCMP2471_c17505_g1_i1
QTLKVGGYFGLWYALNIGYNIYNKKALNIVLPWTMALIQLFAGIPYVMLLWATGLRKAPV
LNLTPSALCHLGTHVGAVLSLGAGAVSFTHIVKASEPVVSALLTAVFLKEFLPIPVYLSL
LPVIGGVGLASLKELSFSWLAFGTAMLSNVASASRAILSKGLMLDAPNLYAVLTMIAFAF
LLPVSLLIETIKTAVDAASLTMISVLSGVYYYLYNEVAFLALNAVAPVTHAVGNTIKRVV
IILASVVVFGNQLTPLGAAGSAIAIFGTLLYSLA
>Pseudopedinella_elastica_c14400_g1_i1
HTLKLGTYFGLWYALNIGYNIYNKKALNVLLPWTMALFQLFAGIPYVMLLWLTGLRVAPK
LNLCPSAACHLGTHVGAVLSLGAGAVSFTHIVKASEPVVSAALSAIFLKQFMPIPVYLSL
LPVIGGVGLASLKELSFSWLAFSTAMLSNVASASRAILSKTVMLNATNLYAVLTIIAFFM
LLPFNFIVESVSAAWDAADMIKYIGLSGIFYYLYNEVAFLALSEVAPVTHAVGNTIKRVV
IILASVIVFQNPLSFLGAMGSAIAIAGTLLYSIV
>Pteridomonas_danica_c34826_g1_i1
RSIKIGVYFALWYSLNIGYNIFNKKALNSLLPWTLATFQLFAGVPYVLTLWATGARVAPK
LNLAPSALCHLGTHIGAVLSLGAGAVSFTHIVKASEPVVSAVLSALFLKQVLPLPVYLSL
LPVIGGVGLASLKELSFSWMAFGTAMLSNVASASRAILSKTVMLTPLNLYAVLTMISFTL
LLPMSLFVETISKAWSNAELVQMVSLSGLFYYLYNEVAFLALSEVAPITHAVGNTIKRVV
IIIASVLVFQNPLSSLGALGSFIAIAGTLLYSIA
>Imantonia_rotunda_RCC918_c4225_g1_i1
ETLTTASFFALWYLFNIGYNIYNKKALNALIPYTMAALQLLVGIPYVGLLWLTGLRKAPK
LTLIPVSLGHLGTHIGAVVSLGAGAVSFTHIIKASEPVVSAGLSALLLGAVYHPITYLTL
LPIVGGVALASLKELSFTWVGFIAAMGSNFASALRGILAKKTMMDEANLYAVLTILAFIF
MVPISLLLEPVASAISAAYLTKMSILAGAFYYLYNEVAFLALGRVNPVTHAVGNTIKRVV
IIIASVIAFKTPISTLGIIGSTIAIIGTLLYSLA
>Chrysochromulina_sp._KOO21229.1

ETVTTGIFFTLWYIFNIGYNIYNKKALNALLPYTMAALQLFVGIPYCALLWATGLRKAPK
LTLIPVSLGHLGTHIGAVVSLGAGAVSFTHIIKASEPVVSAILSFLLLGAVSSWQTYLTL
LPVVGGVALASLKELSFTWVGFIAAMGSNLSSALRGILAKKTMMDEANLYAVLTILAFIA
ILPISLAIESISACIAKAYLWTQSLLAGAFYYLYNEVAFLALGRVNPVTHAVGNTVKRVV
IIVASVIAFKTPISTQGIIGSTIAIVGTLLYSIS
>Emiliania_huxleyi_CCMP1516_XP_005787855.1
ETLKTASFFALWYLFNIGYNIYNKKALNALIPWTMALLQLFVGIPYCLFLWTTGLRKAPK
LTLIPVSLGHLGTHIGAVISLGAGAVSFTHIIKASEPVVSAALSFAILGAVSSWQTYLTL
LPIVGGVALASLKELSFTWVGFIAAMVSNLSSAMRGILAKKTMMSEANLYGVLTILATIA
LFPVALAIEPIRAAWTAAYLAKMSLLSGAFYYLYNEVAFLALGRVNPVTHAVGNTIKRVV
IIVASVIAFKTPISTLGVIGSSIAIAGTLLYSLA
>Aureococcus_anophagefferens_XP_009038840.1
ETLKTGSFFALWYLFNIGRGAFSRKALNAMLPWTLATVQLFAGIPYVALLWATGLRKAPK
LTLFPVAMGHLGTHIGAVISLGAGAVSFTHIIKASEPVVSAALSAVMLKAYYSPITYLTL
LPIVGGVGLASLKELSFTWLGFAAAMLSNVSSALRGILAKKTMMNETNLYAVLTIIAFAV
LLPVSLCVETVGSAIDAADLAVLSALSGAYYYLYNEVAFLALGRVNPVTHAVGNTIKRVV
IIIASVIAFNTPISTLGVVGSSIAITGTLLYSLA
>Dictyocha_speculum_CCMP1381_c41253_g1_i1
RTIKLTTLFGLWYILNIAYNIGNKLVLTALIPWTAATLELFFGLPLVMFLWGTGLRKSPK
LTLSSQAFFLSATHVLGVISFGAGAISFTHILKATEPIWAALISAVFFKEFLPLPVYLSL
VPIIGGVGLASLKELSFTWLSFYAGTLSAVTSAMKAILSKKVLLTPANMFGVLTILGFFM
ILPVSLAIEGVTAAWSAAHLLKLLSVSGFLYYMYNEIAFLALNEVAPVTHAVTNTVKRVV
IIIASVLFFKTPVTMLGAVGSGIAIAGATGYSFA
>Aureococcus_anophagefferens_XP_009036596.1
----LFALFTLWYALNTGYNIGNKMVLNALIPWTSATIELFFGLPYVGLLWASGLRKAPS
LTLCPSAFFLACTHVAGVISFGAGAISFTHILKATEPVWSALISAVVFREVLPLPVLATL
VPIIGGVGLASLKELSFTTVGFVAGTLSAVTSASKAIFSKKVLLTPANMFAVLTILGFLM
ILPASLAVEGVAAAWAAAELWGLLGASGFLYYLYNEVAFLALSEVGPLTHAVTNTVKRVV
IILASVVVFQTPITPLGCLGSGVAIAGALLYSLA
>Pseudopedinella_elastica_c5062_g1_i1
RFTKLTVLFGLWYILNIAYNIGNKLVLNALIPWTAATLELFFGLPLVIILWGSGLRKAPK
LTLASQAFFLMSTHVLGVISFGAGAISFTHILKATEPVWAALIGAVFFKDFLPWPVYLSL
VPIMGGVALASMKELTFTWLSFTTGLLSAVTSAMKAILSKKVLLTPSNMFSVLTIMGFFM
ILPLSLAIEGVTAAWSAAHLFHLLSFSGALYYLYNEVAFIALSEVAPVTHAVTNTVKRVV
IILASVLFFKTPVTFLGAVGSAIAIAGATGYSFA
>Imantonia_rotunda_RCC918_c32644_g1_i1

RTIRLGVLFFAWYFLNTAYNIGNKLVLTAFMPWTAATWELFFGFPYVFFLWTTGLRKAPK
LLLAPSGVLLAGTHVGGVISFGLGAISFTHVLKATEPVWSAFISAVVFREFLPLPVYLSL
VPIICGVGLASLKELTFSWASFIAGTGSAVTSAAKAILSKKVLLTPANMFAILSILGFCA
IAPVSLIIEPAAAAWSAAQLMRLLSVSGFLYYIYNEVAFLALAEVAPVTHAVTNTVKRVV
IILASIIVFKTTITPLGALGSAITVAGALLYALA
>Emiliania_huxleyi_CCMP1516_XP_005772513.1
RTIRLGVLFFLWYSLNTAYNIGNKLVLTAFMPWTAATWELFFGFPYVFLLWSTGLRKTPK
ILLAPSGILLAGTHVGGVISFGLGAISFTHVLKATEPVWSALISALFFRDFLPLPVYASL
VPIIAGVSLASLKELTFSWMSFIAGTGSAVTSAAKAILSKKVLLTPANMFAVLSILGFCA
ILPVSLALEGASAAWSAAYLLKLLACSGFLYYIYNEVAFLALAEVAPVTHAVTNTVKRVV
IILASILVFKTTITPLGAAGSAITVAGALLYALA
>Phaeocystis_antarctica_CCMP1374_c30414_g1_i1
QKLRLTALILGWYVLNTMYNIGNKLVLTAFMPWTAATWELFFGFPYVFILWTSGLRKTPK
LLLAPSGILLAGTHVGGVISFGLGAISFTHVLKATEPVWSALISAIVFRDFLPLPVYLSL
VPIIAGVSLASLKELSFSWMSFIAGTASAVTSAAKAILSKKVLLTPANMFAILSIIGFCA
IFPVTLLIEPAMAAWSAAQLIKLLSVSGFLYYIYNEVAFLALAEVAPVTHAVTNTVKRVV
IILASILVFKTSITPLGALGSAITISGALLYGLA
>Protoceratium_reticulatum_CCCM-535---CCMP-1889_c10262_g1_i1
EKNKVGIYIFLWYLFNIGYNIYNKKALITYYPWACALWQMAFGWLIFVPLWVFGVRKVPK
LTLSPSALGHLATHVGAVVAFFAGAVSFGHIVKASEPVVSSVLNFVFMGEVLAWPVYAAL
LPIIGGVALASASELSFNWLCFGAAMGSNLGSASRAVYSKKVMMDASNVYAVLTIMATFM
LIPISLAIEGMLRGFQAA-FLLHMIASGLFYYMYNEVAFLALGKLDPVSHAVSNTMKRVV
IIITAILVFGTPVTPLGVAGSGIAIVGTLLYSLA
>Protoceratium_reticulatum_CCCM-535---CCMP-1889_c5145_g1_i1

--------------------------------------VSSVLNFVFMGEVLAWPVYAAL
LPIIGGVALASASELSFNWLCFGAAMGSNLGSAARGVYSKKVMMDSANVYAVLTIMATFM
LIPISLAIEGMVKGFSAA-FLLHMVFSGLFYYLYNEVAFMALGKLDPVSHAVSNTMKRVV
IIIAAIIVFGTPVTGLGMLGSSIAVAGTLLYSLA
>Symbiodinium_microadriaticum_OLQ07543.1
DKFGVIIYIVLWYAFNIGYNIYNKKALISYYPWACALWQMSFGWLIFVPLWILRIRKTPK
LTLAPSALGHLATHVGAVIAFFAGAVSFGHIVKASEPVVSSFLNFAFLGEVMPWQVYASL
LPIIGGVGLASASELSFNWLCFGAAMGSNLGSASRAVYSKKVMMDSANVYAVLTIMATFM
LIPISLLIEGMLKGFKIA-FMWQMILSGFYYYMYNEVAFLALGKLDPVSHAVCNTMKRVV
IIITAIFVFRNPVTGLGVLGSSIAILGTLIYSLA
>Synchroma_pusillum_CCMP3072_c12090_g1_i1

QAAKVPVFITLWYLFNIGYNISNKKALNALLPWTIALIQLVGGLLYVFPLWLTGLRKAPK
LTLTPVASMHLGTHIGAVISLGAGAVSFTHIVKAGEPVVSALLSAVVLKKYFSLPTYLSL
IPVVAGVGLASAKELSFTWLGFAAAMLSNVSSACRGIYSKKTMLTAANMYAVLTIIATVI
LTPLALAIEGIGPVWEAAAVIKDVLMSAVYYYLYNEVAFLALEQVNPVTHAVANTIKRVV
IIIAAIIAFGTPVTFLGSLGSGIAIFGVLLYSLA
>Phaeocystis_antarctica_CCMP1374_c15856_g1_i1
KKVQLFSLIFGWFFLNVMYNISNKRVLNAFMPWMMSLSSLFVGVPFVMLLWATGIRKTPE
IVLAPIGAAHAIGHAGSVIALGAGAVSFAQTVKAAEPVFTCVMSYLVLGTVFKWQVYASL
FPIIAGVCIASLKELSFTWLGFIAAMVSNLSSALRGILAKKTMMTEANIYAVLTILATLF
ILPITFAMEPVMKTVNAAYMWTNSLLAGAFYYLYNEVAFLALGRVNPVTHAIGNTVKRVV
IIIASVIAFKTPISTLGIIGSAIAIAGTLLYSLA
>Fibrocapsa_japonica_CCMP1661_c3527_g1_i1
QTLKVGTYFGLWYLLNIGYNIYNKKALNAFAPWTVATAQMIIGMGIFFPLWILKLRKAPK
LTIMPVAVMHTLGHVTGVISLGAGAVSFTHIVKAGEPFFTATLSAVLLKQFFPWQVYTTL
LPVCVGVGLASLKELTFNWVSFGGAMGSNLACSLRGIFSKQMMMTPANLYSVLTIIASVL
LVPAALLFEGLKGLWDAATILLHIFLSGLYYYTYNEVAFLALSQVHPVTHAVGNTIKRVV
IILTSVIVFNTQMTPLGTLGSAMAIGGVLLYSLA
>Phaeomonas_parva_CCMP2877_c3284_g1_i1
NMLKVGGYFALWYILNIGYNIYNKKVLNAFLPWTMALMQLAAGLLVFVPLWATGLRKAPK
LTLLPLASMHTAAHATAVLSLGAGAVSFTHIVKAAEPAFSALFAALFLGQVFAAPVYAAL
VPVIGGVAVASMKELSFSWMSFGNAMGSNTFSALRGIFAKKNMMNAANLYAVLTCMGIGL
LFPIALLTEGWAASWAAGLLAKHMLFSGLFYYLYNEVAFLALSKVNPVTHAVGNTIKRVV
VILASVVVFGTQMTPTAVAGSTVAIAGTLGYALA
>Pinguiococcus_pyrenoidosus_CCMP2078_c14559_g1_i1
QTLKVGGAFALWYILNIGYNIYNKKVLNALLPYTMAAVQLAAGLLIFVPLWVSGIRKAPK
LNLFPLASLHTGAHLTAVLSLGAGAVSFTHIVKAAEPAFSALFAAVLMGQVFAAPVYLTL
IPVIAGVAVASMKELAFSWVSFGNAMGSNTFSALRGIYAKKNMMNSANLYAVLTCFGIAL
LTPIALILEGMGAAWTAALLLWHALLSGLYYYSYNEVAFLALSQVHPITHAVGNTIKRVV
VIIASVIVFGTTMTPTAIAGSSVAILGTLMYALA
>Vaucheria_litorea_CCMP2940_c15515_g1_i1

----------------AVLSLGAGAVSFTHIVKAAEPVFTAILSAILIGQTFALPVYASL
LPVIFGVGMASFKELNFSWMAFGNAMGSNTFSALRGIVGKKQMMNALNLYSVLTIAALIF
ILPAAIAIEGFSATWAAALLLTNIFLSGFFYYMYNEVAFLTLASVHPITHAVGNTLK---

>Ectocarpus_siliculosus_CBN78131.1

SMVKVTAYFGLWYLFNIGYNIYNKRVLNILMPWLMASAQLGIGLLYVFPLWLTKLRKAPK
LPLSQLAALHTVAHVTAVLSLGAGAVSFTHIVKAAEPVFTAGFSAALLGQTFAAPVYLSL
LPIIAGVSLASLKELSFSWVAFGNAMGSNTASALRGILGKKQMMSPANLYAVLTVLAFCF
LSPVALLVEGAKPAWDAAGLSSTILLSGLFYYLYNEVAFLALDSVNPVTHAVGNTIKRVV
IIVAACIAFRTPMTPLSIAGSTIAVAGTLLYSLV
>Fibrocapsa_japonica_CCMP1661_c4076_g1_i1
HRLKIGGYFGLWYALNIGYNIYNKKVLNVLLPWTMGLAQLAIGLLYFVPLWATGLRKAPK
LTLSPLAMCHLGTHMGAVLSLGAGAVSFTHIVKASEPLFTAGLSAVFLQQFMPWQVYATL
LPVVGGVAIASLTELSFSWLSFSTAMASNTFSALRGIFAKKSMMNSANLYAVLTCMSVAM
LTPLALLVEGMKGVWEAASLSTQILLSGLFYYLYNEVAFLALDNVAPVTHAVGNTIKRVV
VIVAAVVFFGTKMTAQGIFGSTMAVGGVLLYSLA
>Fibrocapsa_japonica_CCMP1661_c3975_g1_i1
SVFLILFYLVLWYGLNIGFNIYNKKALNVFAPWTVATAQLVVGWPIFIPLWILGIRKTPK
LVLSPNAAMHTLGHVSGVIALGAGAVSFTHIVKAMEPFFTAVLGAAIMGTIMPWQVYAAL
IPVCAGVGLASLKELSFTWLAFGGAMTSNVACALRGLISKSMMMDASNLFSVLTIYATFL
LTPFAAVLEGLFATWAKATLMMYIGLSGFFFYTYNEVAFKLLGLVDPITSAVANTIKRVV
IIIVSVVVFQTQMTTMGIAGSSLAIGGVLIYSLA
>Phaeomonas_parva_CCMP2877_c4750_g1_i1
HTLKIGVYFFLWYAFNVVYNLTNKQVLNALLPWTISAAQLGIGLLYVFPVWLLKLRKAPV
LTLMPIATCHTTGHLCTVISLSAGSVSFTHIVKAAEPFFSTVMSAVFLKSFFKLPVYLTL
IPIVVGVAMASLKELSFTWVSFLNAMASNTAFSMRAIFSKKQMMSAPNLYAVLTIMSFLG
LAPLAGVIEGIGPAWDSAELTKKILMSGLSYYLYNEVAFLALNAVHPITHAVGNTIKRVV
IIVASVIFFKTKMSMQSIIGSSIAIFGVLLYSLA
>Pinguiococcus_pyrenoidosus_CCMP2078_c5115_g1_i2
QTAKVGIYFFMWYAFNVVYNITNKQVLNALLPWIVSAAQLGLGFLYIFPVWLLRIRSAPK
LTLAPIAACHTTGHLCTVISLSAGSVSFTHIVKAAEPLFSTVMSAVFLKSFFRLPVYLTL
IPIVVGVAMASMKELSFTWVSFLNAMASNTAFSMRAIFSKKQMMSAPNLYAVLTIMSFCA
LAPLALLIESIVSSWQDADLTRKILTSGLSYYLYNEVAFLVLNNVHPITHAVGNTIKRVV
IIVASVFFFNTKMTQQSVVGSSVAIAGVLMYSLA
>Vaucheria_litorea_CCMP2940_c1486_g1_i1
YNLRIGSYFFLWYSLNVGYNYHNKKTLNMIAPLTLSALQLVVGVVYVGLLWLFRLRSAPG
LVLNPIGFCHAASHLSAVVGLGAGSVSFAHIVKAMEPFFTALFSAIFLKQFFPLPVYASL
IPVVAGVALASLKELNFSIVALGGAMGSNVAAASRAILAKKSMMGPANLYGVLTIIATIV
LVPIAAILEGIMALWDAALITRHALLSGLYFYLYNEVAFLALDAVHPVVHAVGNTLKRVF
VIASGVILFGNQITPLGLVGSALAIVGVLVFSLV
>Vaucheria_litorea_CCMP2940_c11004_g1_i1

-AVFPLAALHPPSHISAVIGLGAGAVGFFHIVKSLEPLFTAFLSAVLLKIYLPVPVALSL
LPVVGGVALASLKELTFSWVTFGGAMGSNLAAATRGILAKKQMLDPGNLYGLTTILAAII
LSPAALIVEGFRAVWSAANIIRGMVMSGLYFYLYNEVAFYCLGNIDATTHAVGNTIKRVV
LLGVSVIVFGHQLTPLGLAGSGTAIGGVLLYSLL
>Ectocarpus_siliculosus_CBN74454.1
STLKVGFYLFVWYSLTIGYNIYNKATLNRMIPWILSTVQLAVGAVYVSLIWALGVRKAPK
LAVLPLAALHTTSHIAAVVGLSAGAIGFVQIVKAGEPLFTALFSALFLGQIFALPVYAAL
LPVVGGVAIASLKELSFTWLAFGGAMTSNVAAASRGVLAKASMMDAGNLYGVMTILATIM
LAPFAWLVEGVQGLYDAATLAKGALLSGIFFYLYNEVAFYCLDAIHPVTHAVANTVKRVF
LIAVSILVFGHKLTPLGSIGSAVAIAGVLLYSLA
>Fibrocapsa_japonica_CCMP1661_c2489_g1_i1
--AVPS
ISISVLGALHATGQSTGVVALGAGAVSFTHIVKSAEPAFTAVFSAIMFKQFFSWQVYSTL
LPVCGGVALASAKEVSFNWISFGGAMASNLACSLRGILGKYSMLTPANLYSVLTTFSAIF
LLPLALIIEGIVPMWKTAQLILYCLLSGIFCYGYNEVSFLFLSTVHPVTHAVAGVVRRVV
VVVSALLFFRNPVTPLGAAGSVIALAGTFLYSLA
>Vaucheria_litorea_CCMP2940_c5132_g1_i1
---------LLWYTFNVFYNLYTKWTLNVFAPCTMAALQIVSGLPYVFILWALRIRKAPK
VTIFPVAMAHCFSHLGAVVSLCAGAVGFVQIIKAAEPLFTATLGHVFLGQVMPLPVYLTL
LPVVAGVGLASLKEISFSALAMIAAMCSNMCSGTRSVLGKKIMMDAGNLYAVMTMMAVFV
LFPLAGIVEGLAFLWERAALRKNILLSGLFFYMYNEVAFYCLNAIHPLTHGIGNTIKRIV
MIAVSVVAFKHKFTPLGMLGSMMAIGGVMLYSLV
>Nannochloropsis_gaditana_EWM30451.1
QTLKVGSFFFLWYLFNIGYNIYNKKALNVLLPWMVGLVQLSLGLLYVFPLWLFGIRKAPK
LNILPVAAMHAMAHITAVISLGAGAVSFTHIIKAAEPAFTSLFSALFLKQYFSPVVYASL
IPVMGGVAIASLTERKFSWLAFNSAMLANTASAGRGIFAKKTMMDPANLYAVLTILATLI
MAPVAFLVEGLKPAWEAAQLINYIVSSGLYFYLYNEVAFLALDSVHPVTHAVGNTIKRVV
IIGASILVFKNPVTLQGYIGSAVAIAGVLLYSLA
>Pavlova_gyrans_CCMP608_c20623_g1_i1
QTLKVGGLFGVWYIFNIVYNISNKQILNAFFPWTMAAVQLGVGLLYLFPLWMSPLRKAPK
LNLSPIASMHCIGHLMTVISLGAGAVSFTHIIKAAEPFFSTVMSAIFLKSFFPIPVYLTL
VPVVGGVAIASLTELSFSWLAFLTAMGSNTAFSLRAIFSKMAMMGAANLYGVLTIMSFLA
LTPIALLIETLMAGWNAAWLLKTSFISGLFYYLYNEVAFLCLDNVHPITHAVGNT-----

>Phaeocystis_antarctica_CCMP1374_c3670_g1_i1

ETLKTGSFFALWYLFNIGYNIYNKKALNALLPYSMATLQLAVGVPYVLLLWLTGLRKAPK
MTLIPVSIGHLGTHIGAVVSLGAGAVSFTHIVKASEPVVSAALSAVMLGAFYHPITYLTL
VPIVGGVALASLKELSFSYKSLICALISNVAFASRAVLSKLTMMDAANLYGILTMMAFVL
TLPVAFYMEGLPAKWAASWLVKNIIINGMYYYLYNEVAFLTLNQVAPITHSIANTVKRVA
IIVATCIVFQNPMSRIGVIGSSVAIAGTFGYSYA
>Vitrella_brassicaformis_CCMP3155_CEM22468.1
ETLKTGTYFALWYAFNILYNIYNKQALNVLNPWSVAWAQMFIGIPLFLPLWLTGIRKAPK
LTLIPQGLFHTGTHVGAVVALGAGAVSFAHIVKAAEPVFTAGLSAAVLGQYLSPITYSAL
IPVVAGVGLASAKELSFTWTSFGFAMLSNISSSLRGIFGKKTMLTAANQYSLLTIISCIA
LLPLSAFFEYLVPNWQAAALAKLVVLSGIFYYLYNEVAFLALSRVNPVTHAVGNTMKRVV
IILASIVVFRNPITPLGAIGSGVAILGTLLYSLA
>Imantonia_rotunda_RCC918_c21457_g1_i1
--LKIMTFFAIWYVLNVVYNDTNKTVLKVLLPWTVAALQLGLGLLYVGPLWMLGVKSSPR
LTVAPIAAIHGLGQAVTVMSLGAGSLAFVNVVKALEPLFNVAFGGIFMGDFLPWQVNVCL
LPVVAGVALASAADLSFTWDCFAYAMGSNLAFSLRGVLTKKNSMDAGNTFAVVTTLSFLA
LMPLALYFEGLQSRWQAAELLLRIVASGVSFYAYNEVSFYALDAVHPITHAVGNTIKRVI
LILFSVVRFGTPMTTQSIVGSTIAILGVLAYSVA
>Phaeocystis_antarctica_CCMP1374_c15303_g1_i1
SNLKIVCFFLLWYVLNVMYNESNMTVLKVLLPWSLAALQLGLGLLYVVPKWATGLGKVPR
LAIAPIAMIHGAGQCVTVLACGAGSLAFVNVVKSLEPLFNTVFAALLMGDVLPWQVNACL
LPVILGVGIASATDLSFTWECFGYAMGSNLAYSLRGVLSKRSMMDAGNMFAVLNTLAFLA
VLPVALALEGMASSWERAELVGRVVASGLSFYLYNEVA----------------------

>Pavlova_gyrans_CCMP608_c1379_g1_i2
STPMVLFYFGLWYLINVAYNYCNKKALTVLLPYLSAAFQIGAGLTYVFPLWWLKLRTPPN
LALVPVGMIHGAGQLVTVLSLGAGSISFVNVVKALEPFFNVMFAMVFMKDYVPLPVLLTL
VPVVVGVAIASSSDMEFTWVCFACAMGSNIFFSLRGVLSKVTMLGPANLFAVLTMIAFLT
ALPIALAMELARGKWEAASLAQTLAVSGLSFYMYNEVSFLALDAVDPVTHAVGNTIKRVI
LILLSVVAFGTKMTTLSATGSSIAIVGVFMYSVA
>Chroomonas_cf._mesostigmatica_CCMP1168_c34625_g1_i1
NIVLLGAYFFAWYALNVGYNITNKQVLNVFLYGTTAVAQLLVAWVWLIPQWVAGIRPVPK
PALKKVSVLHGLGHLVTVMSMGLGAVSFVHVVKAMEPVFAAVLSAIFAGSVMAFPVYASL
LPVCAGVAIASAGELSFTWGCFGAAMMSNLLFASRAVFSKMAMMDAANTFAVVTMLATVV
CVPIALLLEGIKAAWAAAKLATTLAMSGWYLYTYNEFAFKVLGMVTPVAQAVGNTVKRVV
ILIATSIAFATPMTPIGIGGSAVAMVGVLVYSLV
>Hemiselmis_rufescens_PCC563_c37853_g1_i1

--VWLIPQWLAGIRPKPS
PALQKVSVLHGLGHLVTVMSMGLGAVSFVHVVKAAEPVFAAILSAIFAGTVMAAPVYVSL
LPVCAGVAIASAGELSFTWGCFSAAMMSNLLFASRAVFSKMAMMDAANTFAVVTMLATVV
CVPIALILEGIPAAWAAAKLSTTLFLSGLYLYTYNEFAFKVLGLVSPVAQAVGNTV----

>Cryptomonas_curvata_CCAP979-52_c11905_g1_i1
NLLLLAVYFVAWYALNVGYNITNKQVLNVFLYATTAVIQLLVAWVWLIPQWMIGIRPVPK
PALQKVSLLHGFGHLVTVLSMGLGAVSFVHVVKAMEPVFAAVLSAIFTGNIMAAPVYVSL
LPVCAGVAIASAGELSFTWGCFGAAMMSNLLFASRAVFSKIAMMDSANTFAVVTMLATIV
CLPVALLLEGIQAAWLAAKLATTMALSGWYLYTYNEFAFKVLGLVTPVAQAVGNTVKRVV
ILIATSIAFATPMTPIGIAGSSIAMVGVLIYSLV
>Guillardia_theta_CCMP2712_XP_005830175.1
NLALLAVYFIAWYALNVGYNITNKQVLNVFCYATVAAAQLIVAWFWLLPQWAIGIRPVPK
PALQKVSLLHGFGHLVTVLSMGLGAVSFVHVVKAAEPVFAAVLSAIFAGSIMAFPVYLSL
LPVCAGVAIASAGELSFTWACFGAAMMSNLLFASRAVFSKMAMMDSANTFAVVTMLATLI
CVPVAAVLEGIMGAWNAAKLASTLALSGWYLYTYNEFAFKVLGLVSPVAQAVGNTVKRVV
ILIATAIAFGTPMTPIGITGSAIAMAGVLVYSLV
>Hanusia_phi_CCMP325_c16900_g1_i1
NLLLLGVYFIAWYALNVGYNITNKQVLNVFCYATVAAAQLIIAWIWLLPQWAIGIRPIPK
PALQKVSLLHGFGHLVTVLSMGLGAVSFVHVVKAAEPVFAAVLSAIFAGSVMAF------
-PVCAGVAIASAGELSFTWACFGAAMMSNLLFASRAVFSKMAMMDSANTFAVVTMLATLI
CVPVAAVLEGIMGAWNAAKLASTLAMSGWYLYTYNEFAFKVLGLVSPVAQAVGNTVKRVV
ILVATAIAFGTPMTPIGITGSAIAMAGVLVYSLV
>Chroomonas_cf._mesostigmatica_CCMP1168_c51832_g1_i1
NKLLVGFYFAAWYALNVGYNIYVKRTLNVCLPFTFAVIQLGAGIVWLAPQWLSGVRGIPK
PALQQVAIFHGLGQLATVVAMGLGSVSFVNVVKALEPIFTAIIGMAVTGVMLPWQVSASM
IPVCAGVALASVSELAFTWGCFGCAMASNVVYATRAVLSKISMMDAANTFAVVTLIAFFY
CLPIALIVEGIGAGLA-AKLAQMVGATGLLYYTYNEMAFLVLGSVAPITQSVGNTIKRVV
VIVAASIVFRTPMTSMGMIGSSVAIGGVLVYSVV
>Hemiselmis_rufescens_PCC563_c27506_g1_i1
NKLMVGFYFLAWYVLNVGYNIYVKKTLNVCLPFTFAVIQLGAGLIWLIPQWASGIRKVPK
PALKKVALFHGAGQLATVMSMSLGSVSFVNVVKALEPIFTAFIGVMVTGVPIPWQVAASM
VPVCAGVGLASVSELSFTWGCFGAAMLSNLVYGTRAVLSKIAMMDSANTFAIVTLIACLY
TIPIAALLEWIGAGLA-AKFVQMVTATGLLYYTYNEMAFLVLGSVAPITQSVGNTIKRVV
VIVAASLVFRTPMTGMGIAGSSIAILGVLIYSVV
>Guillardia_theta_CCMP2712_XP_005821957.1

GRLLLGFYFFAWYVLNVGYNIVVKKTLNICLPWTFAVIQLGAGILWLAPQWLSGIRAIPK
PALTKVAVFHGFGQLATVTAMGLGSVSFVNVVKALEPICTALIGLIVTGRNLPWQVWLSM
LPVVGGVGLASASELSFTWGCFLAAMFSNVVYATRGVLSKESMMTAENTYAVVTLIAFVL
MLPFALFLEGVASGLAMAKLAQMVVATGLLYYTYNEMAFLVLGSVAPVTQSVGNTVKRVV
VIVAAAIVFQTPMTPLGIIGSSTAILGVLLYSVI
>Compsopogon_caeruleus_SAG-36.94_c4183_g1_i1
RTLKVGLYIGLWYAFNIVYNISNKRTLNALLAWMVSWLQLVVGIIWGAGLWVLRLRKAPK
LALTPVAIAHTVGHVGTVASLGAVAVSFTHVVKSLEPFVNVLGSAIVLKSIFPTPVYLSL
LPIVAGVIMASVSEVSFTWMGFLSAMSSNFAFTARNIISKINMMNSTNLFTIIQIISVII
LAPFALWMD-MKGAWLGASLFWNLIISGLFFQLYQEVSYKALDSVHPVTHAVANTVKRVV
IIVTSIIVFRNPITRFNAMGSTIAILGVLLYSMT
>Madagascaria_erythrocladioides_CCMP3234_c12584_g1_i1
QTLKVGLYIGLWYAFNIVYNITNKRLLTMYLAWFVSWGQLVVGIVWAGLLWLLRIRKAPK
LSLAPVAAAHTIGHVATVASLGAVAVSFTHVVKSLEPFVNVLASAVILKSVFPIPVYLSL
LPVVAGVIMASVTEVSFTWMGFLSAMGSNFAFTGRNIFSKLNMMDAVNLFSVIQVISVVI
LAPFAFLAD-LKSTWLAAAIIYNFLMSGLFFQLYQEVAFKALDSVHPVTHAVANTVKRVV
IIVTSIIVFGNPVTRANATGSTIAILGVLLYSMT
>Rhodosorus_marinus_UTEX-LB-2760_c4304_g1_i1
-------FIVLWYVFNIVYNISNKTVLNVYYPWFVAWIQLVIGAAYALLVWATKLRKMPD
VSLIPVAIFHNVGHVATVVSLGAVAVSFTHVVKAMEPFVNVVASAIILKSVFPIPVYLSL
LPVVGGVIMASVTEVSFTWMGFLSAMTSNFAFVGRNIYSKLGMLNGANLFAVMSCIAVVL
LAPFPFIME-FITGWNAVKLAQTIGISGLFFHMYQEVAFLALDSVHPVTHSVANTVKRVV
IIVTSIIVFKNPVTQANALGSAIAILGVLLYSLI
>Chondrus_crispus_XP_005713953.1
STLKVGFYIFLWYAFNIVYNISNKKVLNWFLPWFVSWFQLLVGVLYVLPLWGLRLRKAPV
VTLLPISVGHVIGHVSTVVSLGAVAVSFTHVVKSMEPFVNVVGSGVFLQSFFPLPVYLSL
LPVVAGVVMASVSEVSFTWLGFLSAMTSNFAFTARNIFSKLSMMGPANLFAVLSVMSTLL
LAPVALIIDHLIAAWKKAKLIAGLLISGLFFYLYQEVAFKALDSVHPITHAVANTVKRVV
IIVTSVFVFQNPVTKANAMGSAIALLGVLLYSVM
>Gracilariopsis_chorda_PXF44201.1
NRFKVGLYILLWYAFNIVYNISNKRVLNWFVPWFVSWVQLLVGVLYVLPIWALRLRRAPT
VTMLPISLGHIVGHVSTVVSLGAVAVSFTHVVKSMEPFVNVVGSAFFLNSVFPLPVYLSL
LPVVTGVVMASVSEVSFTWLGFLSAMTSNFAFTARNLFSKLSMMDPANLFAVLSIMSTIL
LAPVALIIDHLMSAWNTAKLMLYIFISGLFFYLYQEVAFKALDSVHPITHAVANTVKRVV
IIVTSVFVFQNPITKANALGSSVALLGVLLYSVT
>Erythrolobus_australicus_CCMP3124_c13133_g1_i1

---MVGVYIALWYAFNIVYNISNKKALNAFLPWTVSWLQLAVGCVWVALLWALRIRKMPR
VALLPIAAAHAVGHVSTVYALGAVAVSFTHVIKSMEPFFNVVGSAVVLRSVFPLPVYASL
LPIVVGVILASVSELSFTWLGFGMAMMSNVAFTARNIFSKLNMMDALNLFAMLQLLSTAL
VLPFMLLLE-IRGMWNAAKLALTLGVSGLFFQLYQEVAFLALNSVHPVTHAVANTMKRVV
IIITSIFVFKNPVTRANIGGSAVAIAGVLIYSLV
>Timspurckia_oligopyrenoides_c2381_g1_i1
STLKVGLYIALWYAFNIVYNISNKKALNALLAWFVSWIQLVFGLAWIFPLWIFKVRKVPK
LAILPIAIAHTVGHVATVASLGSVAVSFTHVIKSMEPFFNVIGSAIVLRSVFPTPVYVSL
LPIVAGVIVASVSELSFTWLGFASAMLSNLAFTGRNIFSKLNMMDAVNLFAVIQIISSVI
LAPFALAVD-LKAAWTAAQLLSTLFVSGLFFQLYQEVAFLALNSVHPVTHAVANTMKRVV
IIITSIFVFQNPVTLANMSGSAIAIAGVLIYSLV
>Rhodella_maculata_CCMP736_c1125_g1_i1
---------------NIGYNIYNKQTLNAILPWLVSWVQLAVGAVWVLGLWAVRLRKVPT
VAMVPVAAAHTLGHVSTVLSLGAVAVSFTHVIKSMEPFFNVVASAVILKSVFPIPVYISL
LPVVVGVIIASVSEVSFTWGGFISAMVSNFAFTARNIFSKMSMLGPVNVFGILSIISTLL
LAPLVLIFE-IKSTWLAAQLLTLLLASGLFFQTYQEVAFLALGSLHPVSHSVANTIKRVV
IIITSIIVFSNPVTPAAMIGSSVAILGVLLYSLT
>Porphyra_umbilicalis_OSX80082.1
QTLKVSILIGMWYLLNIGYNITNKRVLNWFFPWFVSWAQLAFGVVYCVALWAVKLRKKPV
VALSLVALCHTIGHVSTVCSLGAVAVSFSHVIKSAEPLFQVAASAIFLKSVFALPVYLSL
LPVIGGVVLASVSELSFTWLGFLSAMTSNVAFAARNIFSKLSMMGPVNLFGVLTVLSTLI
LAPFALFFE-LKAGWLAATLAGYILASGFFFHTYQEVAFLALSNVHPITHAVANTTKRVI
IMGVSILVFKNPLTQNGLIGSSIALLGVLLYSFA
>Galdieria_sulphuraria_XP_005707012.1
RQLKVASYFFLWYAFNIVYNISNKKLLNAYFPWTVAWVQLAVGVFYVVPLWLLHLRKAPH
IRLLPVAAAHTIGHISTVVSLGAVAISFTHVVKALEPFVNVLASAVILRSVFPIPVYLSL
LPVVGGVIIASVTELSFTWTGFMAAMLSNFAFTSRNIFSKISMMSPANLFAVLTILSTFI
LLPVALILE-LYQGWILAQLITGLLTSGLFFYLYNEVAFYALDSVHPITHSVGNTMKRVV
IIITSLLVFKNPITPANAIGSAIAISGVLLYSLT
>Cyanidioschyzon_merolae_strain_10D_XP_005536532.1
RRLKIGSYFLLWYLFNIVYNISNKTVLNAMGGWIVAWLQLALGIPYILLVWTLGIRKAPT
IKLLPVAAAHTLGHLCTVLSFGAVAISFTHVVKALEPFVNVVGSAIFLRSVFPLPVYASL
IPVVAGVIMASVSEATFNWMGFLTAMGSNFAFTARNIFSKINMMTPMNLYAVLTILSTFL
LLPFALIAE-FPAAWRAAKLLVWVGVSGLFFYLYNEIAFMALDSVHPITHAVGNTVKRVV
IIIASVIVFKNPIDWRGWLGSAIAIGGVLLYSLV
>Bolidomonas_pacifica_CCMP-1866_c2786_g1_i1

PSIKAPAYFLLWYGLNIGYNIYNKKVMNAYLPFTMATIQLGAGLLWILPAWILGFRAKPI
LSLIPIALFHTIGHTMTVVSLGAGAVSFTHIVKAAEPFFSTTIQAIM-GNVQPMSVNLCL
IPIVGGVALASMKELSFSWTSFGGAMGSNLSFAIRGIFSKKAMMGPANLFAVLTIMSFAF
MLPVCIIVEGMSTSLPSATFQTEVLVAGLFYYLYNEVAYLALGVVDSTTHAVGNTIKRVV
VLVASTIYFKNPMSGQSMVGCGVAIGGVLLYSVV
>Bolidomonas_sp._RCC2347_c6512_g1_i1
TLLKTTAYLGLWYFLNVLYNLSNKSVATSFLPLTLASTQLFAGLLYILPCWALGLRPVPT
LRVAVVAVFHTVGHCLTVLSLSAGSVSFTHIIKSAEPIFSTLLLSLL-GTHSPLPVNLML
LPIVFGVSLASMTELSFTWPSFLGAMGSNLSFAVRAIYSKRLMLTPTNLFAVLTIISALL
TAPVVLVLEGIMPALEAAAFLAALFTSGLTYYTYNETAYLALSLFSTTTHAVANTVKRVV
ILVASTLYFKTEMSAQSVAGCGIAVGGVLGYSIV
>Fibrocapsa_japonica_CCMP1661_c4568_g1_i1
ETAKLGIFFLMWYAFNVAYNIYNKKVLIYFFPWMASLISLGVGLVYLLPVWILGIRAAPK
LKLSPVAALHCIGHVMTVIALGAGSVGFVHIVKAAEPLFSTVLNYLINGAMQPFMVNLCL
LPVIGGVCMASLTELSFKWSVFLGALGSNTCFASRAIFSKKVMLSAANLYGVLTILSFIG
LLPFALIVEGLIAGYNKAQFYKEQMICGLAYYLYNEVAYMVLGQVGAVTHAVANTVKRVA
ILVASVIIFGTTMTQQGIIGSSIAIGGVLLYSLT
>Synchroma_pusillum_CCMP3072_c5196_g1_i1
SAVMLTFYIVLWYALNVAYNIYNKKVLNVFLPITFGTLQLGIGLLYVLPVWFLGLRKAPD
LTITPAAAFHTVGHIATVVALGAGSVSFTHIIKASEPIFSTILSALILKEFQPLVVVATL
LPVVGGVAYAAMSDLSFTWTSVGYAMLSNLSFATRSIVSKKTRMPMSDFYAIMTIISFLA
MVPFAVIIEGVAAAFAEAELLKQMLFCGLSYYLYNEVAYLALGQLTSVTHAVANTVKRVV
IMLAEVVLFSKPMSPRKATGAAVAIGGGLLYSLT
>Chromera_velia_CCMP2878_c20814_g1_i1
DNGITAALILAWYALNALYNVDNKKALNIALPYSISIIQLFVGWLWFLPLWGTGLRQAPK
LRIVPQGICHLLVHLGAVVAMGMGAVSFVQIVKAMEPAFMAALSAAVLGQYFSVWTYLAL
VPVIGGICLASASELSFTWIAFWGANVSNMGSALRAIYAKMVMLTPANMYSMLTIVSTIA
CIPCALIMEFFGPAISAAEILYPMVMSGIWYYSYNEVAYVALGRVHQVTHAVANTIKRVV
IIGVTIIVFQNPVDTKGYIGSAIAILGTLLYSLS
>Vitrella_brassicaformis_CCMP3155_CEM04483.1
ETLITLGLVALWYFLNTWYNIHNKKAVNMLLPWTISTIQLWIGWLWFWPLWLTGGRKMPI
IKIAPQGVFHLGVHAGAAVSMAAGAVSFTHIVKAGEPAITALLAGVMLKEVFHPLTYLAL
VPVVGGVAVAAMKELSFTWLSFTGAMVSNLGSSLRSIFAKRVQLDSANLYSLLTIVSTLL
STVVALMIEGISGEWEKASIAWYTLLSGVWYYSYNEVAFITLGRLNAVTHAVANTLKRVV
IILASVIVFRNPISLVGGIGSAIAVGGTLLYSLA
>Toxoplasma_gondii_ME49_XP_002365260.1

FYAQLGVMLLFWYALNVMYNLDNKLALIMLLPWTVSTFQLFFGWLFFGFAWATGLRPVPR
IRIAPQGLCHFFVHIGAVISMGCGAVSFTHIVKASEPVLTALLSGLALHQVFSWQTYLSL
VPIVAGVIMASVTELSFTWKAFGCALVSALGSSARAVFAKLAMLSSANMYALLTIVASLV
SLPPAIFAEGVAAVWEACQIIAKLCFSGLWYYMYNEVAYLCLEKINQVTHAVANTLKRVV
IIVASVLFFQTPVTALGATGSFVAIAGTLIYSLS
>Vitrella_brassicaformis_CCMP3155_CEL94695.1
STAYTALLICGWYFFNTIANVENKRTLNMVLPCTVSAIQMLVSWVWFVTLWGTGIRKLPR
LAIVPQALMHIGNHVTGQLSFAAGAVSFTHVVKANEPVFTALLSMVLLKQTFSIWTYLFL
LPIIFGVALAALKELSFTWIGLWGAIFSALFSALRSVYAKRVMMTPGNIFSLLTIVSTFL
SLPIALVVDYPEGAWAEGTVIWSAARSGLFFYLYNETAFIALSRIHALTHAVANTLKRVV
VIVTTVIVFRNPITPLGAIGSVIAIFGTLVWSIA
>Pavlova_gyrans_CCMP608_c22617_g1_i1
RTARIGACFAVWFALNIMYNISNKVCQNAFMPWTMATVSLAVGVPYVLLLWATGLRKAPK
VKLVPIGLFHALGHASAVIALGAGAVSFTHIIKAAEPVFTCVLSYLVLGQTFSLPVYLSL
IPIVGGVALASLKELSFTWKALIGAMMSNIAFASRAIYSKRQMLGAANLYGLLTIIALVA
TVPVALWFEGFQAEWAKVWMIQQMLMNGVYFYSYNEVAFYTLSQVAPVTHAVGNTLKRVA
IIATSVIVFKNPLSQLSIIGSTIAILGALLYSLA
>Chrysochromulina_sp._KOO31396.1
KTVRLALCMFVWFFLNVMYNITNKKCQNAFMPWTMTVVSLFVGIPYVLLMWASGLRKAPK
LTLLPIGAAHALGHAGAVIALGAGAVSFAQTVKAAEPVFTCVLSYLVLGTVYKWPVYASL
LPICGGVILASLKEMSFTWKALYGALTSNIAFASRAVLSKATMMGAANLYGVLTIIAFLL
SLPFAIYYEGFAAAWAKSWLIRQMTLDGFYYYAYNEVAFITLNQVSPITHSIANTFKRVA
IILATVLVFGNKLTPLGAAGSAIAVAGTFLYSLA
>Emiliania_huxleyi_CCMP1516_XP_005758490.1
DKAKLGFLFFSWYFLNVMYNITNKRCQNAFMPWTMTVVSLFVGIPWVLFLWGTGIRKPPK
ITLLPIGAAHALGHAGAVIALGAGAVSFAQTVKAAEPLFTCALSFFVLGTVFKWQVYASL
LPIIVGVSLASLKELSFTMKALYGALTSNVAFASRAVLSKKTMMNAPNLYGVLTIIAFIL
SLPFAVYYEGFASQWAAS-----------------KVAFIALDQVSPITHSIANTIKRVC
IILATVLVFGNKLTPIGAAGSAIAVAGTFLYSVA
>Bolidomonas_pacifica_CCMP-1866_c17021_g1_i1
SKVKVPSLFLLWYMLNVYYNIVNKKVLNVVAPITISAVQLLIGSVYWAATVLLKLKPVPK
LNIGAAAFSHSCGQTLTVVSLGAGAVSFTHIVKALEPFFSAMMSIFFTKKVMHPNVYLSL
IPVVGGVGLACLKELSYSHVAFFAALGSNLFFAIRAVKSKQVMLTPSNLFGIQTCIATVV
GLIFALGMEG-LKAVDIVLLLRAIFLSGLFHYLNNEVMYMTLGQVDAVTLAVGNTMKRVF
IIVASVIVFGNQVSTESAIGSAVGIGGVLVYSLT
>Dictyocha_speculum_CCMP1381_c14095_g1_i1

```
STMKVLFYFAVWYALNVVYNIKNKQVLNRLLPWLVSVAQLAVGALYALLVWASRLRAFPD
RALLPIGLFHGAGQALTVLSLGAGAVSFTHIVKAMEPFFSAIISAIVSKKVLKPQVYATL
IPVVGGVSIAVAKDVSFSAVSFGCAMSSNLAFACRAVFSKSAMLTAANLFGVVTILAFLM
MLPLALLAEGFRARWGDAELVTQILLSGFFHYTNNEVMYMALSNVHPITLAVGNTLKRVV
IIVAALIVFRNPMSTTTAVGSAVGIGGVLLYTLT
>Florenciella_parvula_CCMP2471_c34785_g1_i1
------------------------------------------------------------
----------------------AGSVSFSQIVKAMEPFFSAIMSVVATGKVLHPVVYLTL
LPVVGGVSIAVAKELDYSHLAFGTAMGSNLAFGCRGVYSKLAMMNAANLFGVLTILGCLF
TIPFAVASEGFSANW------KAVVASGIFHYLNNEVMYLALSNVHPITLAVGNTLKRVV
VIVASLIVFRNPITPVAAIGSAVGIAGVLLYSLT
>Phaeodactylum_tricornutum_CCAP_1055/1__XP_002180816.1
HTLKVGFYFALWYALNIVYNILNKKLLNVLSPVTVGSIQFGVGCFYVLLVWALKLRPAPT
LAVQKVGFWHCTGQLASMVSLGAGPVSFTHIVKALEPFFSAVVSALAFGTWMKPQVYATL
LPVVGGVGYACLKERSFSWLAFYMAMGSNLAFALRAVLSKVAMISSTNVFAMVTLAAFVW
SIPMALVTEGFGTLWNKADLCKALFVSGMFHYLNNEVMYLALGNVHPVTLAVGNTMKRVI
IMVASVMVFQNEITPQAAVGSAIGIAGVLLYSLT
>Nitzschia_sp._NIES-3581_TPT2
ARLVVGFYFFAWYALNVVYNIVNKKVLNVLAPLTVATIQLGVGAVYSALVWMLRLRAYPK
LAVGFVGLYHCLGQLATVMSLGAGPVSFTHIVKALEPFFSAVLSGLYFQKWMRGGVYATL
IPVVGGVSYACLKELNFSWVALSAAMASNIAFALRAIMSKLAMLTPPNMFGVVTWAAFFI
SLPLCVFGE-FLTLLGDAQFIQSLLLSGLFHYLNNEVMYLALGKVHPVTLAVGNTMKRVF
ILVASVMVFQNPISQQAAIGSTVGIAGVLLYSLT
>Fragilariopsis_cylindrus_CCMP1102_OEU14710.1
QRLEIGGYFAAWYALNVVYNIINKKVLNVLAPLIVGSIQFGIGALYCALVWIFKFRPFPK
LAVASVGAYHMLGQLSTMISLQAGPVSFSHIVKAVEPFFSAIVSGFYFNSWMKPQVYATL
IPVVGGVGYACLKELNFSWLAFGAAMASNLFFALRAVLSKSALLTAPNMFGLVTVTAFLL
SIPVALIREGFSALWLAAQFVRAIFISGLFHYLNNEVMYMALGSVHPVTLAVGNTMKRVF
ILVASVLVFRNPISMQAGIGSAVGISGVLLYSLT
>Pseudo-nitzschia_multiseries_Psemu1|258158
QRLEIGGYFAAWYALNVVYNIVNKKVLNVLAPLVVGSIQFGIGALYCAVVWLLRFRPCPT
LAVTAVGAYHMLGQLATMIALGAGPVSFTHIVKAVEPFFSAMVSGFYFNKWMPPQVYATL
IPVVGGVGYACLKELNFSWLAFSAALSSNLFFALRAVVSKSALLTAPNMFGLVTMAAFAI
SIPVGLFMEGFPSLWAAAQLVRAIIVSGLFHYLNNEVMYMALGSVHPVTLAVGNTMKRVF
ILVASVLVFRNPVTVQAGIGSAVGISGVLLYSLT
>Thalassiosira_pseudonana_CCMP1335_XP_002288027.1
```

VRLRVGSYFALWYILNIVYNILNKKYLNVIAPLTVGSLQFLVGSLYSILLWGTKLRPRPV
LEVNKVGFYHMMGQELSMMSLGAGPVSFTHIVKALEPFFSAVVSAVVFGKWMHPMVYATL
IPVVGGVAYACLKERSFSWLAFWTAMGSNLAFALRAVVSKSALLTSVNLFGIVTCYAFIQ
SIPLFLLGEGFLDLWKKADLVRGLAVSGLFHYLNNEVMYLALSNVHPVTLAVGNTMKRVF
IVVASVLVFRNPITVQAAIGSAIGIGGVLLYSLT
>Pseudopedinella_elastica_c11099_g1_i1

---ARAGSLYGVVTWLAFLL
MLPLALVAEGAREAWRAAQLAQQVLLSGLFHYTNNEVMYLVLNNVHPITLAVGNTLKRVA
IILAALLVFRNPITPAAAIGSAVGVAGVLLYSLT
>Vaucheria_litorea_CCMP2940_c2123_g1_i1
-------------------------VLNRLLPYLVGTFQLGIGGLWVALQWLSKIRSFPK
LRALWVGFFHGGGQLTTVLSLGAGAVSFTHIVKSAEPLFSALVSALCFGQIFKPQVYLTL
IPIVGGVAVACAKEINFNVTSFVAAMVSNAFFGLRSNFSKSLMMDPANLFGIVTILGFLI
CLPLALILEHVFSVWKDAILVGNLILSGLTHYMNNEVMYLALGSVHPVTLAVGNTIKRIF
LILVGMIVFNDRITLLGGVGSVVAIVGVQLYSIA
>Nannochloropsis_gaditana_EWM29609.1
GTVLTLSYFALWFALNVYYNIVNKRVLNALLPFTVATAQLGIGAVYVSLSWLLRVRASPQ
IDLLSIAFYHGSGQLFTVLSLGAAAVSFTHIVKALEPFFSALVAAVCFKQVFKPQVYLSL
LPVVSGVAVACANDVSFGWLAFLTAMASNLAFALRANFSKGALMSPANVYGLVTILAFFL
LLPLTCLWEGWRPSWRVATLVHQLLLSGLFHYLNNEVMYLALDNVHPITLAVGNTAKRVF
IIVASLLVFRNPITLTGAVGSGVGIAGVLIYSLT
>Ectocarpus_siliculosus_CBN76065.1
DRARVLGYFGLWYALNVWYNIVNKKVLNALLPSSIAVLQLGIGSLWVGTQWLVRARTPPK
LRLAPVAFFHGGGQLATVLSLGAGAVSFTHVVKAMEPFFSALVAAVWFRQIFRWQVYASL
LPVVAGVSLACAKEINFSWVSFLAAMASNLLFACRANFSKALMTSSANLYGLVTIVSFVV
FAPFAALTGWWGPAWESAALVLSVLLSGISHYLNNEVMYLALGSVHPTTLAVGNTMKRVF
IVVASLIVFKTPISRLGMVGSAIAVGGVLVYSLA
>Synchroma_pusillum_CCMP3072_c1722_g1_i1
HRAKVLSYFLLWFALNVVYNISNKSVLNRLLPWTVATVQLLVGSLYVALLWLLRLRPVPS
LLAIRVALAHGLGQLATVISLGAGAVSFSHTVKAGEPIFSALFSALLFRQIFPAPVYLSL
VPVVGGVALACAKELSFTWLCFAAAMASNVFFALRAIFSKAAMISAQNLYGVVTIGALLV
LAPFALVMEGAPGAWAAATLAWDLVKSGFWHYINNEVMYLALSNVHPVTLAVGNTFKRIF
IIASSVLFFNNPVNLLTMMGSSVAIMGVFLYSLV
>Bolidomonas_sp._RCC2347_c21418_g1_i1

EPATTVSLFLGWYALNVYYNIVNKRVLNVLFPMTISTFQLLTGSVYYFTVLL--FRGKPE
LQVYAAAAGHAIGQTATVVSLGAGAVSFTHIVKSLEPFFSCIVTYFSTKKIMDFRVYLSL
IPVVGGVGLACLDKLSYNHLAFFAAMLSNLAFAIRAVKSKQALLDAPNLFGIVTTIAAAM
CIPVFLAFEGCFKGIDSLNLLYKVLLSGLFHYLNNEVMYLTLNRVTPVTLAVGNTVKRVF
IIAASVIVLGNQVGTKSIVGSAVAISGVLLYSFA
>Aureococcus_anophagefferens_XP_009035574.1
KRLEVGAVFALWYALNVYYNVLNKKVLKVVLPWLVATAQLAVGGLYSLGVWAAGLRAGPD
LAALPIAAAHGAGQAATVVSLGAGAVSSTHVIKALEPLFSAAVNAGVNGEVLPLGVYASL
LPVIGGVGGAVATDLSFNPLSFAAAMASNLCFAFRAVCSKNAMLGAPSLFGVVTLGALLL
VAPVALALEL--PGLSAAGLAASLACSGLFHYLNNEVMYLALARVHPVTLAVGNTLKRVV
VILAALVVFQEPMNLATAVGTAVAIAGVLLYSVL
>Phaeocystis_antarctica_CCMP1374_c5013_g1_i1
---------------------------------------IVVGALFAVCLWLTGLRTPPT
GDLLPIGAFHAVGHIAGIVGTSQGSVSFAQVVKSAGPIYACVLSSLVLKQAVSLRVWASL
LPILGGVALATTSELTLAWAALFGAVISDVALALRNIYCKVSMLSAANTFALTTCLAALF
CVPLAAAPP---------------------------------GEVAP-------------

>Emiliania_huxleyi_CCMP1516_XP_005762442.1
QRLKVGGYFALWFLLSVGYSITNKRVTNALCPWSVATATVVVGSLFVNLLWLTGLRRRPR
LALIPIGTFHAIGHIAGTVGTAAGSVSFAQVVKAAGPVYACVLSA----AAVSLRVWLSL
APIIAGVGLATLKELSFAWAALLGAVASDLALALRNVLSKQSMLAPADMFGLLTCISAAV
SVPAALLVEGLPALWASAGLAGQVAATGLYFYGYSEVAMKALNNVHPVTHAIGNTMRRVV
IMLVCMVAFRTPMTPLGACGSALAIGGSYLYATV
>Emiliania_huxleyi_CCMP1516_XP_005757540.1
RTLKIGVYFALWFALSTGYNIANKVRLNAILPWCHSAASLGVGSAFVSFLWATGLRKRPS
LLL-PISFLHALGHIGAVVSAGAGAVSFTQIVKAAEPVCTALLSWGILGATISAPAALAL
VPIVAGVALASVSELSFTWLSFSGAMLSNLAFATRNILSRASMMTPENLFGVLTVMSFLW
ALPCAILIEGALAKWSAA--LQYTVSTGLYFYLYNEVAMLALNNVNPVTHAVANTLKRVV
ILLACVVFFKTPMTPLCIAGSTVAIVGSYLYSMA
>Dictyocha_speculum_CCMP1381_c41321_g1_i1
DFLQTTFYIVLWYGLNVGYNIYNKDTCNAFYPWTVGCLSLGCGLLYILPVWVLGFRKIPN
MKIGAIAVLHTAGHFGAVISMSAGAVSFTHIVKAAEPVFTTILSGIINGAWSPAVVNLTL
VPVVAGVALASMKELSFTWLAFIGAMVSNLSFSLRSIFMKRALLDSANIYAVLTIFSFIL
SIPLALYFEGLMEELPGLEIVKLNVITGLFYYLYNESASLALGKLNSVAHAVCNTVKRVV
IMIAVNAYFSKPMSDQSKLGSGIAIGGTLLYALV
>Florenciella_parvula_CCMP2471_c12478_g1_i1

--LDTVFYVALWYGLNIGYNIYNKDTSNQFFPWIIGCISLGAGLLYMLPVWLLGVRKIPK
LKIATIAALHTIGHFGAVLSMSFGAATFTHVVKAAEPVFSTILNGLINKSWAPMQVNLTL
IPVVAGVAWSCMTSIEMNVNAFIGAMVSNLAFSLRSIYMKNALLDSANVYAVYTIFAFIL
SIPLAYYFEGLASEWPTIEIVKLNIITGLFFYLYNESASLALGNLDGVAHAVCNTVKRVV
IMIAMVSGIDPAMSTQKMTGAAVAIGGTLLYAVV
>Pseudopedinella_elastica_c8734_g1_i1
EGMMTLVYIGLWYALNIGYNIYNKDLCNGFLAYTVGTTSLGAGLIYVLPNWYLGIRPVPK
MNIGIISVLHCVGHFGAVISMSMGAVSFTHIVKAAEPVFSTVLAGLILNKWAAWQVNLSL
VPVILGVVIASVKINDFNMGAFVAANISNLAFALRSMYMKKMDLDSANIYAVYTIFAFIL
AIPLALYMEGVVALSGSMKLMEMHLITGLFFYLYNEASSLALGNLDSVQHAVCNTVKRVV
IMVAM-SFFDTPLTLQKWTGAGIAIGGTLVYTLV
>Aureococcus_anophagefferens_XP_009038003.1
----------LWYAFNVGYNVYNKMLSKALFPMLIALTSLGVGLLYFVPLWILGLRKAPK
LACTVLSMLHTVGHVGAVVAMSAGAVSFTHIIKALEPMFSVFFGYVLTGKIDSLKVNIWL
VPIIAGVGWAAVGTKDINPVAFGGAMTSNLAFSLRGLLSKRVKLTSSNLYAVLTLISFFL
FLPFALVLEGLAAAWPPP---ELVLWTGFFYYMYNEMAYLVLGEVSATAQAVANTVKRVV
ILLATVAFLGESMDQNKAAGAAVAIGATMLYSIA
>Chromera_velia_CCMP2878_c18_g1_i1
DKLTLAGLIIGWYGLNVLYNIDNKKALNMLLPYTISAVQMWVGIPIFLLAWALGINKVPS
LEIAKSGAFHAGVHLSAVVALGAGAISFVHIVKAGEPVTTAIINYLVSGAAVPAPVAACL
IPIIAGVGLASLKELSFTWLAFGGAMLSNICSSSRGAFAKQFQLDASNQFALLTAFSAVF
LLPFCLVEKNWTSVFNAAEILKHVIGSGVWYYLYNEVAFRSLKKLDPVSHAVANTVKRVA
LILVSVVVFGSQFTTLGAAGSAIAVAGTFLYAYA
>Vitrella_brassicaformis_CCMP3155_CEM07668.1
ETAKTFFFLFLWYAFNVLYNVDNKRALNLYLPLTVSALQMLVGIPIFMLPWLTGIRPKPK
IQLAIQGLFHASVHISAVIALGAGAISFVHIVKAAEPVTTSVLSAVVLQNYLSPLTYLSL
VPIIFGVSLASLKELSFTWRAFIGAMVSNVGSSLRGIYSKKAMLTPSNMYAILTIFATMF
LVPLALFDMKIVPVWNNAQIIRHVVGSGFWYYLYNEVAFLSLSRLNTVSHAVANTFKRVA
LILASVIVFGTKFTPLGALGSAIAVGGTLLYGVS
>Alexandrium_catenella_OF101_c6891_g2_i2
GALAMAAGFFFWYAANVSFNIVNKQALGLFHAWAVSVIQLATVVACSAIGWVTGLVESP-
LRLLPAAICHAVGNGLTSVAFSCGSVSFTHVVKTSEPVWMAMGNFLVTGAVLQRKQVLAL
VPVMLGVGLASAGELSFTWVGFLAALGSTIAFAGRGIFSKRLMMSALNVYAMDSMMALLF
TLPVALIAEGLAPVLAGQ-LATLLAFTGLTYYAYNAIAFKLLAKLDVVSHAVGNLGKRIF
VILFSVLAFSTPFSTRAAIGSTLAILGSGIYSYV
>Protoceratium_reticulatum_CCCM-535---CCMP-1889_c8552_g1_i1

GAGPMAMGFFLWYAANVAFNIVNKQALGLFHAWSVSVVQLATVVACCSVGWATGAIPSP-
LRLLPAALCHALGNGLTSVAFSWGSVSFTHVVKTSEPVWMALGNLLIMGARLPAPQLLAL
VPIMLGVALATAGELSFTWLGFLAALGSTICFAGRGIFSKRLMMSPLNVYALDSLLALAF
TLPVALVADGLG-LAAGQ-LAGLLAATGLAYFAYNAIAFQLLGKLDVVSHAVGNLGKRIF
VIGFSVLAFSTPLTLRAVVGAAVAILGSGVYSYV
>Micromonas_commoda_XP_002505063.1
------MYFGLWYFLNVQFNIINKQIYNYFFPWFVSAIHLAVGLLIMTFFWTTRLVKFEK
PAVTLPSFLHAFGHCLTNVSFAAVAVSFTHTIKTLEPVFSAIGSYLVTGTVYAWPVYMAL
VPIMGGVALASATELSFTWLGFSTAMASNVAFSARAIFSKKLMMSPLNLYNFVTIVSLLF
CIPFVIAFEGIAKAVELKEFVLALLKVGAFYHLYNQVAYQALGKVEPVTHAVGNVGKRIF
VIGFTILAFGNKISTQTAIGSAIAVVGAGLYGWL
>Ostreococcus_tauri_XP_022841169.1
PQAETVFYFAAWYFLNVQFNIINKTIYNYFFPWFVSCVHLFVGLFIMAFFWGSKLVEYEQ
PALSLPAFLHAFGHCLTNVSFATVAVSFTHTVKTLEPVFTAIGSYLVAGTVYPLPVYASL
LPIMGGVAIASATELSFTWLGFLTAMSSNVAFSARAIFSKKLMMSPLNLYNWVTIVALMF
CLPFAIYFEGISDAIALKEFLMALASVGFYYHMYNQVAYQALGKVAPVTHAVGNVGKRIF
VIGFSILAFGNKISTQTAVGSLIAILGAGIYGVV
>Oryza_sativa_XP_015619081.1
PALITGFFFFMWYFLNVIFNILNKKIYNYFYPYFVSVIHLLVGVVYCLVSWTVGLPKRAP
ILLFPVALCHALGHVTSNVSFATVAVSFAHTIKALEPFFNAAATQFVLGQQVPLPLWLSL
APVVLGVSMASLTELSFNWTGFINAMISNISFTYRSIYSKKAMMDSTNVYAYISIIALIV
CIPPAVIIEGFNDAIAKVKFVSDLFFVGLFYHLYNQVATNTLERVAPLTHAVGNVLKRVF
VIGFSIIVFGNRITTQTGIGTCIAIAGVAIYSYI
>Oryza_sativa_XP_015637713.1
PALVTGFFFFMWYFLNVIFNILNKKIFDYFYPYFVSVSHLLVGVLYCLVGWSFGLPKRAP
ILLFPVAVCHAIGHVTSTVSFAAVAVSFAHTIKALEPFFNAAASQFILGQQVPLTLWLSL
APVVIGVSMASLTELSFNWTGFVNAMISNISFTLRSVYSKKAMMDSTNLYAYISIIALLV
CIPPAIIIEGFKDAIAKVKLVSNLLVVGLFYHLYNQVATNTLERVTPLTHAVGNVLKRVF
VIGFSIIAFGNKITTQTGIGTCIAIAGVALYSYI
>Arabidopsis_thaliana_NP_001078720.1
PWLVTGFFFFMWYFLNVIFNILNKKIYNYFYPYFVSVIHLFVGVVYCLISWSVGLPKRAP
IVLIPVAVCHALGHVTSNVSFAAVAVSFTHTIKALEPFFNAAASQFIMGQSIPITLWLSL
APVVLGVAMASLTELSFNWLGFISAMISNISFTYRSIFSKKAMMDSTNVYAYISIIALFV
CIPPAIIVEGFADAIAKVKFISDLFWVGMFYHLYNQLATNTLERVAPLTHAVGNVLKRVF
VIGFSIVIFGNKISTQTGIGTGIAIAGVAMYSII
>Coccomyxa_subellipsoidea_C-169_XP_005646002.1

PSVVTLSFVTMWYGLNVAFNLLNKTIFNYFFPYTVSAVHVVVGLAYCSLTYLLGAKKASA
IQIFGPAAMHAVGHIAANLSFAAVAISLTHTVKTLEPAFNVLLSKLFLGVGTPLPVVSTL
IPIMMGVALASASDLTFNWTGFISAMVSNLTFGFRAVWSKKAMLDSTAIYAYTTLISVLI
CVPAALIFEGLQAASAKADFYFSLFLVGLLYHLYNQFAFNTLSRVSPVSHGVCNVVKRVV
IIGTSVIFFGTTLTMKTKLGTGIALLGTYLYTEA
>Compsopogon_caeruleus_SAG-36.94_c2639_g1_i1
RTLKVGFYFGLWYFFNVVFNVLNKSTLNVWYPWTLSLVQLGGGSLYCALLWILGLRQKPN
VAMILPVLGHLGGHVLTCVSFSMVAISFSHVVKSAEPAFGAAAAALALREFYPWTVYASL
IPIISGVALAAVTELTFTWAGFITALLSNVAFAARNVFSKITMLGSRNMYGLISIVAFLL
ELPMALIADGIPKLIPATTLLLYILSSAIMYHLYNESSYMALGQVSPVTFSVGNTVKRVI
IIVASILVFKTKFLPLNAFGMIIALLGTFLYSWT
>Madagascaria_erythrocladioides_CCMP3234_c5060_g1_i1
SRLRIGFYFGLWYLFNVIFNVLNKSTLNLWFPWTLSLVQLGVGSSYCALLWILNLRKRPN
VAMVLPVLGHLAGHVLTCVSFSHVAISFAHIVKSAEPAFGAVGAALATGEVYPLGVYASL
IPVIVGVALSAVSELTFTWWGFVTAMLSNVAFAARNIFTKITMLTAQNMYGLTSIMAFLF
ELPMAMIADGIPSLAATRTVLTYLVASACFYHLYNETSYLALAEVSPITFSVANTFKRVI
IIVSAVFVFKTKILPLNAVGMAIAIVGAFLYSLS
>Erythrolobus_australicus_CCMP3124_c10419_g1_i1
STTTVGFFFALWYLFNVVFNIVNKKTLNMWYPWTLSTVQLGVGALYCSLAWLFGLRVKPN
VAMVLPAFGHTLGHVMSCLSFSLVAISFTHIVKSAEPVFGAAMAGLVLKETYPFTVYLTL
IPICLGVALSSASELTFTMAGFATAMASNFAFALRNVFSKITMMTPANNYGLITIMSFLM
ELPLCLYFDGIPSLAATKTVLKYLLASSLLYHLYNEVSYSALDNVSPITFSVGNTIKRVI
IILTSIIVFRTKILPLNAIGSVTAILGTFLYSSA
>Rhodosorus_marinus_UTEX-LB-2760_c5821_g1_i1
STMKVGFYFGLWYFLNVIFNIINKQTLNMWYPWTLSLVQLGVGATYCSIQWLIGTRKRPN
VALTLPAAAHTLGHIMSCLSFSSVAISFTHIVKSAEPVFGAVCAAAFLGEAYPFYVYLTL
IPIIFGVALSSATELTFTWMGFITAMISNFAFAMRNVFSKITMLTSENIYGLISIMAFLM
ELPFAVYFDGIPAMVA--TLFRYFMSSCLLYHLYNEVSYLALGNVSPVTFSVGNTIKRVI
IIGASILFFKTKIMPLNAVGSVIAIVGTFLYSMS
>Rhodella_maculata_CCMP736_c18737_g1_i1
---MLWVLGLRRRPN
VAMFLPSLGHAVGNVASCVSFSAVAISFSHIVKSAEPAVGAVFSALLLGEVYHPMVYLSL
LPIIGGVALCSATELTFNWFGFATAMSSNFAFAGRNVFSKMTMFTPQNSYGLISIIAFLM
QLPFCLLFDGIPSLLPTSTIFMYLMASSLLYHLYNETSYLCLNNVSPVSFSIGNTIKRIC
IIFASIIFFKTTILPLNALGCAIAIFGTGMYAYT
>Porphyra_umbilicalis_OSX69157.1

QLLKVGSLFLLFFSINVCFNITNKRLLNLWVPWTLSTVQLGTGALYCSLLWVLGLRKKPN
VALMLPSLGHTIGHVGSCISFSYMAISFAHIVKSAEPAFGAVASAVFLGEFFPFSVYASL
IPIIGGVAMAAVSELTFQWPGFLLAMMANVGFAARNVFSKLTMLPAENLYGLISIISFLM
ELPFCLMADGIPSLGVNPAVPGLFFASSMLYHLYNEVSYLCLYNVSPVTFSVGNTLKRVF
IIVASIIAFKTKVLPMNALGMVIAIAGTALYSWT
>Galdieria_sulphuraria_XP_005704181.1
HTLKVGFYFFLWYFFNFIFNIANKRTLNMWYPWVLSTIQLGVGALYCTFLWVLGLRTKPN
VALIWPSLGHTLGHAATCMSFSLVAISFTHVVKSAEPVFGAVGSALVLGEFFHPLTYLTL
VPIVSGVALSAATELTFTWTGFITAMISNVAFVTRNITSKFTMLIAQNTYALITIISFFM
ELPFALLMEGFPPLVSAIKLFGSIMFCSLFYHLYNEVSYLCLDNVSPVSFSIGNTIKRVI
IIFGSILVFRTPVTRLNFIGSTIAIIGTMLYSLA
>Timspurckia_oligopyrenoides_c3008_g1_i1
QTLKVGFFFGLWYAFNVVFNIVNKKTLNIWYPWTLSTVQLGVGSLYCTLAWLTGLRVKPN
VAMLLPAFGHTIGHVMSCLSFSLVAISFTHIVKSAEPVFGAVMAGLVLGETYTLPVYLTL
IPICVGVALSSASELTFTMAGFLTAMASNLAFALRNVFSKITMMTPANSYGLITIISFLM
ELPFCLLADGIPSL-------------------------------ISP---------K---
---------------------------GIPE----
>Cyanidioschyzon_merolae_strain_10D__XP_005537646.1
KRLKVGFWFFMWYLYNVVFNIVNKKTLNMWYPWVLSTIQLGVGALYVSVLWLLGLRRRPQ
VSLILPSLFHTIGHATSCLSFSSVAISFTHTVKSAEPVVGALGSALFLHEYYSPMVYFAM
IPIIVGVALSSISELTFTMAGFLNAMASNFAFVARNVTSKVSLLTAFNTYGLITIISFFL
ELPMALLFE-LPKV----TVFGYIAVASLLYHLYNEASYGVLEDVSPLTFSIGNVVKRLA
IILSSVIAFGTIMRPLNWLGVALAVGGTLIYSYA
>Gracilariopsis_chorda_PXF50002.1
DTLQVGFLFFLWYVFNIIFNLMNKTVLNAWKPWILSTVQLGVGSIMVLTQWALRLQKRPN
LALFLPTISHLVGHVSTCISFSYVAVSFSHIVKACEPAFGALGSALVLGEVYSPGVYATL
LPIISGVALSAVTQFQFSWPGFLFAMLSNLAFASRNIFSKLTMLSPQNIYGIMSVMAFMI
EVPIALSVEG-LSALPSSHIARVLAGSGVFYTLYNTVSFMALGKTGVVTHAVGNILKRAS
VIVVSIFFFRTPVKLFNAVGMGIALAGTFLYSIV
>Arabidopsis_thaliana_NP_197265.1
KTLQLGIVFGLWYFQNIVFNIFNKKALNVFYPWLLASFQLFAGSIWMLVLWSFKLYPCPK
IALLGPALFHTIGHISACVSFSKVAVSFTHVIKSAEPVFSVIFSS-LLGDSYPLAVWLSI
LPIVMGCSLAAVTEVSFNLGGLSGAMISNVGFVLRNIYSKRSLIDGLNLYGCISILSLLY
LFPVAIFVEGWVPGYHKAPSTFYVLLSGVFYHLYNQSSYQALDEISPLTFSVGNTMKRVV
VIISTVLVFRNPVRPLNALGSAIAIFGTFLYSQA
>Arabidopsis_thaliana_AAM63660.1

KKLKIGIYFATWWALNVVFNIYNKKVLNAYYPWLTSTLSLAAGSLMMLISWAVGIVETPK
TTLFPVAVAHTIGHVAATVSMSKVAVSFTHIIKSGEPAFSVLVSRFILGETFPTSVYLSL
IPIIGGCALSALTELNFNMIGFMGAMISNLAFVFRNIFSKKGMVSGMNYYACLSMLSLLI
LTPFAIAVEGWVDGWQTAQFVWWVVAQSVFYHLYNQVSYMSLDQISPLTFSVGNTMKRNS
VIVSSIIIFRTPVQPVNALGAAIAILGTFLYSQA
>Arabidopsis_thaliana_OAP12842.1
QKLKIGIYFATWWALNVVFNIYNKKVLNAFYPWLTSTLSLACGSLMMLVSWAIRIADAPK
TTLFPVAVAHTIGHVAATVSMSKVAVSFTHIIKSGEPAFSVLVSRFFMGETFPLPVYLSL
LPIIGGCALAAITELNFNITGFMGAMISNLAFVFRNIFSKKGMVSGMNYYACLSMMSLVI
LTPFAIAVEGWAAGWQNANFVWWVVAQSVFYHLYNQVSYMSLDQISPLTFSIGNTMKRIS
VIVASIIIFHTPIQPVNALGAAIAIFGTFLYSQA
>Oryza_sativa_EEC83014.1
QKLKISIYFATWWALNVIFNIYNKKVLNAFYPWLTSTLSLACGSAMMLVSWATRLVEAPK
TVLFPVAVAHTIGHVAATVSMSKVAVSFTHIIKSAEPAFSVLVSRFLLGETFPVPVYLSL
LPIIGGCALAAVTELNFNMVGFMGAMISNLAFVFRNIFSKRGMVSGMNYYACLSIMSLVI
LTPFAIAMEGWAAGWQKANVVWWVAAQSVFYHLYNQVSYMSLDEISPLTFSIGNTMKRIS
VIVSSIIIFHTPVRPVNALGAAIAILGTFLYSQA
>Physcomitrella_patens_XP_024374417.1
RRVKIGIYFATWWALNVVFNIYNKKVLNVFFPWLTSTLSLAAGSAIMLISWALRIVPAPD
VGLAPAALAHTIGHVAATVSMSKVAVSFTHIIKSAEPAFSVIIQRLLLGEDFPLPVYLSL
LPIVGGCGLAAATELNFNMTGFVGAMVSNIAFVFRNIFSKKGMVGGMNYYACLSMMSLVF
LTPFAIAVEGWTAGWDAAKIFWWVVAQSVFYHLYNQVSYMSLNEISPLTFSIGNTMKRVT
VIVSSIIIFHTQVQPMNAVGAAIAIFGTFLYSQV
>Physcomitrella_patens_XP_024358298.1
HRLKIGTYFAVWWGLNVVFNIYNKKVLNAYMPWLTSTLSLAAGSAIMLISWALKIVDPPE
VSLAPVALAHTIGHVAATVSMSKVAVSFTHIIKSSEPAFSVIIQKLVFGENFPLPVYLSL
LPIIGGCGLAAATELNFNMTGFAGAMISNIAFVFRNIFSKKGMVGGMNYYACLSMMSLVF
LTPFAFAVEGWTTGWQAAQILWWVVAQSVFYHLYNQVSYMSLNEISPLTFSIGNTMKRVT
VIVSSIIIFHTKVLPINALGAAIAIFGTFLYSQ-
>Physcomitrella_patens_XP_024364494.1
SKLNIGMKFAVWWSLNVVFNIYNKKVLNVYFPWLTSTLSLAAGSGIMLISWALKILKAPE
VSLAPVALAHTIGHVAATISMSKVAVSFTHIIKSSEPAFSVIIQRIVFGDKFPYQVYLSL
LPIIGGCALAAATELNFNMTGFTGAMISNIFFVFRNIFSKKGMMGGMNYYACLSMMSLVF
LTPFAIAVEGWTAGWQAAQVFWWVVAQSVFYHLYNQVSYMSLDKISPLTFSVGNTMKRVT
VIVSSIIMFNTKVSPINAVGAAIAVFGTFLYSQV
>Oryza_sativa_XP_015646103.1

RRAKIGVYFATWWALNVIFNIYNKKVLNAFYPWLTSTLSLAAGSAIMLASWATRIAEAPA
TALSPVAIAHTIGHVAATVSMAKVAVSFTHIIKSGEPAFSVLVSRFFLGEHFPAPVYFSL
LPIIGGCALAAITELNFNMIGFMGAMISNLAFVFRNIFSKKGMVSGMNYYACLSMLSLVI
LLPFAFAMEGWAAGWQKANFVWWVAAQSVFYHLYNQVSYMSLDEISPLTFSIGNTMKRIS
VIVASIIIFHTPVQPINALGAAIAILGTFIYSQA
>Physcomitrella_patens_XP_024371666.1
KRFPIELYFAVWWSLNAVFNIYNKKVLNAFFPWLTSALSLAMGSVFMLSLWGLRLVEPPD
VGLAPVAILHTIGFVAATVSLSKIAVSSHHIIKSLEPACSVIISKLFMGEDFPLSVYFSI
VPIIGGCGLAAASEVDFSMIGFLGAMLSNIAFVFRNIASKRGMVGGMNYYACLSMMSFVL
LLPFAFVVEGWAAGWTTAQFPLWVVLQCLLYHLHNQVSYMSLDQISPLSFSIGNTMKRVT
VIATSILIFRNPVSPINAIGAAIAILGTFFYSQA
>Chlamydomonas_reinhardtii_XP_001701487.1
SNWKLPVYIVLWYAFNIIFNIVNKSTLNTFCPWFIGTWQLVASGLFMAFLWITRLHPVPK
VALMPVALFHTVGHIAAVVSFSQMAVSFTHIVKSAEPVFSVALSGPLLGVGYPWYVWASL
LPIVAGCSLSAMKEVSFAWSGFNNAMISNMGMVLRNIYSKKSLIDGINLFGLISLASLIY
CVPASLYFEGWKGMWEASQL---LLWGGFFYHLYNQLSYMVLQGISPVTFSVGNTMKRVA
VVVSSVMFFKNPVSGLNWIGSFIAILGTYLYSLA
>Helicosporidium_sp._ATCC_50920_KDD73011.1
QTAKMATYVFGWYAFNIVFNILNKSALNVFAPWFIATLQLVASALFMVGLWATGLHPVPR
VALLPVALFHTIGHVSACVSFSQMAVSFAHVVKSAEPVLSVALSQALLGEFNPWYVWLSL
LPIIAGCSLAAMKEVSFAWNGFNNAMISNLGMVLRNVYSKKSLLDGINLFALLSILSLIY
LIPAALFMEGWKDMYAVAQLVKLLALSGVFYHLYNQASYMVLAGISPVTFSVGNTMKRVA
VVASSILFFRNPVSALNWIGSLVAMLGTGLYSAA
>Ostreococcus_tauri_XP_003078079.1
VKTKTAVYFFLWYFFNIVFNVYNKSTLNVFYPWLISTLQLAATSLWMLTVWATKIQPKPE
VAVAPVAFFHTVGHVSACVSFSKMAVSFTHVIKAAEPVFSVILSGPLLGQTFAPAVWASL
IPIVAGCSMAAMKEVSFNITGFQGAMISNVAMVLRNITSKKSLIDGINLYGILGIIGLFY
LAPAAYMIEGWSAGYAAAKLWQMLFLSGIFYHLYNQVSYQALTNITPVTFSVGNALKRVA
VIVASVIYFRNPVSPLNAAGSALALVGAYLYTKA
>Micromonas commoda_XP_002506268.1
QTLKVSLYIFGWYFLNAIFAIMNKKTLAVFYPWILSWIQIAVGAVFMLIMWKLRIFKPPE
GALIPTSFYHMVAHVSACASYKFGSVSFMQVVKAGEPAIAVLLLSMFFGRKYSWRVWLTL
IPIVGGVAVGSTTEINFSMAAFLCAMTSNVTSALRAATSKDLQLKGINLYGGIAIVSGIM
LLPLSLLVEGMGAAFAAAGFMAYLIIGSMFYHLYNQTAYQALGELTPLSHSVANTVKRVV
IILASVAVFKNPITPLGQVSAAIAILGTFIYS--
>Micromonas commoda_XP_002503997.1

--LRVSSFIFFWYFLNAIFAIINKRTLSVFYPWLLSWVQIAVGAAFMLVMWRLRVFKPPS
TALWPTSCLHLVAHVTACASYSLGSVSFMQVVKAGEPACSVILLTLFFGRKYSKLVWLTL
IPIVGGVAVGSTTELNFSMASFVCAMISNVASALRSVTSKDLQLRGINLYGAMSVVGAVV
LLPISLIVEGLPAAFASAPFLAYLFVGSMLFHLYNQTSYQALGELSPLDISVANAVKRVV
IILASVAVFRNPITPLGAWAGAVAILGTFLYSLA
>Thalassiosira_pseudonana_CCMP1335_XP_002289533.1
-TLQVPLYFILWYVLNVLYNITNKWALQDILPITIGCLQFAIGSVYACTLWMLGSRPVPE
VRMSHIAIHHTLGQLCTVLTLAANSISFAHVIKAMEPFFSAIASRFFLGQRMDIRVYLAL
VPVVGGVMMACAGSNEFSWVSFGFGMGSNAFFAMRAVSSKTDEMSPSNLFAAVTCMSFIF
SVPIGIILEGLIDLFKFIKTIMYVLSSGLFHYLNNEVMYLVLSNVHPITLAVGNTMKRVF
IIVAGVLVFSTPVTTSTAIGSTVGIGGVFVYSLM
>Plasmodium_falciparum_3D7_XP_001351641.1
EKLKLALLFLTWYTLNVLYNVDNKKALNMVLPWFISSMQLYVGWIFIFIYWISGMKKIPK
INILIQSVCHIFVHFGAVMAMSATSVSFTHVVKACEPVFTAIFSILLLKQYLKINKYIAL
LIIVGGVVCASMKELHFTWIAFWCATLSNFGSSIRSIYAKKMMLNASNIYAFITIISALI
SLPLVLAFEGETYNFLVNDVIFKIILSGMWYYFNNEVAFMCLERVNQITHALANSIKRVV
IIVSSIIIFKTQITLLGAIGSAVAIFGAFLYSIF
>Chroomonas_cf._mesostigmatica_CCMP1168_c30839_g1_i1
NMGKLAVLLVLWYALNVQYNLYNKKILNAFYPYTVSLTQLGAGLFYVLPIWTLGIRTFPS
VKFGLLAGFHGCGHFATVISLGAGSVAFANVVKAAEPFCAVLMGVIIQGSIPPVAEMGAL
LPIIAGVMIASMAEPEFSMVAFTMAMASNFLFAARGTLGKILMISGADVFAINTIFAFII
MAPVAIYFEGIQAAWNKYYFAAYLLVCGLYYYTYNEMAFMVLDLLDPVGQAVGNTVKRVV
IIIAGTIVFNKPLTQQGIIGSAVAIGGVLLYSLV
>Hemiselmis_rufescens_PCC563_c3273_g1_i1
NLVMLAVYMVLWYTLNVQYNLYNKKILNAFYPYMVSLSQLASGIFYILPIWLLGLRSFPS
FKMGLLGFFHGSGHFATVISLGAGSVAFANVVKAAEPFCSVLMGVLMTASFPPAMELLSL
TPIIAGVMIASMAEPEFSQTAFIFAMSSNLLFAARGTLAKLIMIGGADVFALNTLFALVI
MAPLAVYMEGIQDAWAKYYFAAYMLVCGLYYYTYNEMAFMVLDLLDPVGQAVGNTVKRVV
IIIAGTIVFNKPLTQQGIIGSSVAIGGVLVYSLV
>Guillardia_theta_CCMP2712_XP_005829341.1
SIPLLLFYLTAWYVLNVQYNLYNKKILNAYFPYTTALIQLGSGLLYIIPKYALGFAKWPS
FNISLLSFFHGGGHYATVMSLGAGSVAFANVVKAGEPLCSVLMGFLFNGAIPALMELIAL
LPIIAGVMIASMAEPEFSMFAFGCAMLSNFLFAARGTYAKICMMSGADLFAMNTIFAFVL
MAPITFVMEGAITGFEQLYFIAYQLVCGLYYYFYNEMAFMVLDLLDPVGQAVGNTVKRVV
IIVAGTIVFNKPLTTNGIIGSSVAIGGVLLYSLV
>Hanusia_phi_CCMP325_c10464_g1_i1

--ALMELISL
LPIIAGVMIASMAEPEFSMFAFGCAMLSNFLFAARGTYAKICMLSGADLFAVNTIFAFVL
MAPITFVMEGAISGFEKLYFIAYQLVCGLYYYFYNEMAFMVLDLLDPVGQAVGNTVKRVV
IIIAGTIVFNKPLTTNGIIGSSVAIGGVLLYSLV
>Rhodomonas_salina_CCMP1319_c23779_g1_i1
NPIKLIFYLVAWYALNVQYNLYNKKILNVFYPASCSLVQLGAGMFYVFPQWILGIRKFPK
VTLIVVSCCHGAGHFATVISLGAGSVAFANVVKAAEPLFSVLMGVIFAGSFPSMGEGFSL
LPIIAGVMIASMAEPEFSQTAFVFAMLSNVLFAARGTFAKKMMVSGADGFAISTFLSFVV
MAPVAFYMEAVVKAWEALYFAAYNLVCGLYYYTYNEMAFMVLDILDPVGQAVGNTVKRVV
IIIAGTIVFNKPLTQNGIIGSSVAIGGGLLYSIM
>Cryptomonas_curvata_CCAP979-52_c11280_g1_i1
STVKLVVIIVLWYVLNVQYNLYNKKILDKFYPYTTSLIQLGSGIFYVLPIWTLGIRKFPG
FKVATLSAFHGGGHYATVLSLGAGSVAFANVVKAGEPLCSVIMGIIIMSVFPSTSQVLTL
IPIVAGVMIASMAEPEFSTMAFVCAMLSNVLFATRGVLSKPLMISGSDLFAINTFIAFII
MAPVTFALEGIVKAWNEFYFCAYQLVCGLYYYTYNEMAFMVTDLLDPTSQSVANTVKRVV
IIIAGSVVFNKPLTQNGIIGSAVAMLGVLLYSLA
>Synchroma_pusillum_CCMP3072_c6905_g1_i1

------SLCSAVGHAATGVSLNAGAVSFTHIVKAMEPFFNTLMGVLLVGAVVPPTVVATL
VPIVGGVVLASVKELSFSWGGLMGGLASCIALSLRAIVAKAAFLSPANLYGHVNRAAFVV
TALAAVVVEGLRGGAGLATCLVNVLLSGAVFNLYQEVSFMSLRRLHPVTHAVVNTVKRVV
LVMVGVVVLQEQLSPRASVGAAVSLTGVLLYSLA
>Pavlova_gyrans_CCMP608_c16999_g1_i1
KLLKLGTLFGAWYLFNIWYSLIAKMVLQWWSPWLFTILQLGIGSLWVILQWLVTLRPVPA
IAMLPIGACLAAGHLASTMAMFYGTVAFANVVKTAEPLFTCLFSALVLHQFFSLPTYLTL
VPIMGGVAYASAKELSFSWISLGTAMASNVAFALRAILAKISMLSPANLFAVVNMIAFVL
LLPLALIIEGAAANWKGAELIKLIVATGVTYYLYNELAFMCLGTVHPVTHAVANTIKRVA
VIAVSIIYFKNPVTKEGIAGSAIAVVGVLAYSLM
>Imantonia_rotunda_RCC918_c10865_g1_i1
EQAKMPLAFTGWYLMSIVYSLLNKEVLTVWFPCVFSAVQLLVGALWICLLWTAPIRTPPS
MKVAVVASWLALGHVLSTVAPAYGTVAFTNVVKTLEPLFTCFFSYVFLQQVFPLPVYLSL
LPVIAGVAVASANEIGFSTISLVTGLASNVCFALRAITAKRMMLNAQNLYGVLTLLALGA
ILPGALLVEGLVAGTLATKFAKMLLAAGLSHYVYNECAFLALSSIHPVSHAVANTIKRVA
VIVLSVIYFRNPLTVTGASGSAVAVVGVFLYSLA
>Chrysochromulina_sp._KOO24092.1

ESMKVPLAFIGWYVLSIVYSLLNKEVLNVWFPVVFSAVQLLVGALWIGALWMAPVREPPR
LQVSIVALWLALGHVLSTVSPAYGTVAFTNVVKTLEPLFTCFFSAIFLKQVFSVPVYLSL
VPVIFGVALASANEVSFSMISLISGLLSNVCFALRAISAKRVMLDAQNLYGVLTIVALVA
IVPLALLAEGIVAGTLATRFLRMLLYSGLSHYTYNECAFLALDSIHPVTHAVANTIKRVA
VIVLSVLYFRNPLTLTGALGSAIAIVGVMLYSIA
>Phaeocystis_antarctica_CCMP1374_c31373_g1_i1
DTLFVPLAFAGWFLFNIQYSMLNKKVLMVWFPFTFAALQLLVGSLWICGLWLASIRTRPS
VQLLPISTCLALGHALSTVAPAYGTVAFTNVVKTLEPLFTCALSAVFLGEIFSLPVYLSL
VPVIAGVLLASTNEVSFSMISLVSGLLSNLFFALRAISAKGVMLSPTNMYGLLTLQSLAL
LLPLALLIEGLAAGTAATPFVRMLLATGVTHYVYNECAFIALSAVHPVTHAVANTVKRVA
VIVITVLYFRNPMTYLGTAGTTMAILGVLLYSLA
>Emiliania_huxleyi_CCMP1516_XP_005793996.1
RQLKIPLAFTCWYLFSIVYSIANKQVLSAWFPCTSAAAQLAVGALSVLLLWTLRLRSPPS
LKLVPVASCLAAGHLLSTVAPAYGTVAFTNVVKTLEPLFTCAFSALLLGQTFPLPVYLSL
LPVVFGVCLASASEVSFSAVSLTSSLLSNVAFALRAIAAKSVMIG------------SCL
PLPSCPRLD-VSRTCLGRTFARQLLLLGTSHYFYNECAFLALSSVHPVTHAVANTVKRVA
VILISLVVFRNPLTPAGALGSAVAIGGVFLYSLA
>Pavlova_gyrans_CCMP608_c15472_g1_i1
---------------------------------------LAVGAVWSLLQWAVSLRPGPQ
IKLLPIAVCVAAGHLVTTIAYTHGSVAFTNIVKTAEPAFAVALSSAALGARFSSWTVASL
VPIVAGVAIASANEISFSWVTLLTALCSNAAYALRAVLAKLYLMSPATLFGVVNLLACCV
LFPFAMLVEGLEVSWLRAQLRGLVALAGVTYFLFNELAFMTLAVVSPVTHAVANTLKRVA
VVATSVAYFRVPVTAQGAAGSALAVIGAHLYSVS
>Chroomonas_cf._mesostigmatica_CCMP1168_c55904_g1_i1
WALKTTSYFGLWYFFNIFYNVANKKALNALLPWLQSLVCVAVGMPYIALVWLFKVRDVPK
LELIPIISLHSAGNVGGNVAFGAGALGFAHVLKSMEPAFTAVFSGMLTGKWQHPAVYATL
IPVMGGVAYASASELNFNMLQFVAAMVSNVGFSLRAVIGKQIMLDGPNTFSVLQIGSSLV
TLPFVAAIEGMHPNWKAAYLWQNLIMSGMAFQLYYEAAFLALDAVSPITHSIGNNIKRIV
IVITSVIIFGQKMSAKSMVGSSIALGGVFIYSMV
>Hemiselmis_rufescens_PCC563_c12472_g1_i1
WALKTTSYFGLWYFFNIFYNVANKKALNALLPWLQSLVCVAVGIPYILATWQFGIREAPR
LELLPIVSLHAAGNVGGNVAFGSGALGFAHVLKSMEPAFTAVFSGLLTGKWQHPFVYATL
IPVMGGVAYASASELNFNMLQFVAAMVSNVGFSLRAVIGKQIMLDGPNTFGVLQIGSTLV
TIPFVLAIEGMHPNWKNAYLWNNLVMSGMAFQLYYEAAFLALDAVSPITHSIGNNIKRIV
IVITSVIIFGQKMSTKGMIGSGIALGGVFIYSLV
>Cryptomonas_curvata_CCAP979-52_c8793_g1_i1

WALKTTTYFGLWYFFNIFYNVANKKSLNALLPWMQSFACVAVGVPYILVLWLLKVQATPV
LDLIPIVSLHSAGNVGGNVAFGAGALGFAHVLKSMEPAFTAVFAGLLTGKWQHPWVYATL
VPVMGGVAYASASELNFNMLQFVSAMVSNIGFSLRAVISKDIMMDGPNTFRVLQIGSSLV
TIPFVLAVEGTHPNWKAAYLYSQLFLSGMAFQLYYEAAFLALDAVSPITHSIGNNIKRIV
IVVTSVIIFGQKMSTQSMIGSSIAMAGVFIYSLV
>Rhodomonas_salina_CCMP1319_c13914_g1_i1
WAVKTTTYFGLWYFFNIFYNVANKKALNALLPWLQSLACVAVGVPYILFMWATGARSKPV
LQFFPTVVLHAAGNVGGNVAFGAGALGFAHVLKSMEPAFTALFGGLITGKWQHPVVYSTL
VPVMGGVAYASASELSFNWLQFAAAMVSNVGFSLRAVLSKGVMTDGPNVFSLLQIGSTFV
TIPFVVAMEGLHPNWKAAYLVTQLVLSGLAFQLYYEAAFLALDAVSPITHSIGNNIKRIV
IVITSVIIFGQKMNTQSMVGSSIAIGGVFLYSLA
>Guillardia_theta_ABD51933.1
WSIKAGSYFGLWYFFNIFYNVANKKALNALLPWLQSLACVGVGIPYIALIWALGVRDTPK
ISIIQQSSLHAAGNVGGNVAFGAGALGFAHVLKSCEPAFTAIFSGLINGKWQHPFVYATL
IPIMGGVAYASASEVNFNMLQFVSAMVSNVAFSLRAVLGKKTMLDGPNTFSVLQIGATLL
TIPFVVAVEGTHPSWKAAYLWKQLILSGLMFQLYYESAFLALDAVSPVTHSIGNNIKRVV
IVITSVIIFGQKMSTQSMIGSSIAIAGVFLYAQV
>Hanusia_phi_CCMP325_c3416_g1_i1
WSIKAGSYFGLWYFFNIFYNVANKKALNALLPWLQSLACVGVGIPYIALIWALGVRDTPK
ISIIQQSSLHAAGNVGGNVAFGAGALGFAHVLKSCEPAFTAIFSGLINGKWQHPFVYATL
IPIMGGVAYASASEVNFNMLQFVSAMVSNVAFSLRAVLGKKTMLDGPNTFSVLQIGATLL
TIPFVVAVEGTHPSWKAAYLWKQLILSGLMFQLYYESAFLALDAVSPVTHSIGNNIKRVV
IVITSVIIFGQKMSTQSMIGSSIAIAGVFLYAQV
>Lotharella_globosa_LEX01_c23166_g1_i1
ALSCLLLLFALWYFFNVFFNIYNKQVLKAMVPFSMTLMQFFIGSLIVAMVWMSKAHPFYK
PKIVPLALLHTLGNLFTNISLNAVAVSFTHTIKASEPFFAVVMSWIFLASPPTLLVVGSL
VPVVGGVTLASFTEATFNWIGLSTALASNLTFQTRNVVSKKLMLDNINLFSYMTLMSFAM
LLPVSLWMEGWLPSVMAAQMVAKAAFAGFFFHSYQQVSYLILQRVHPVTHAVGNCVKRVA
VIVISILFFANPVGLLNALGIGVAMCGVLVYSVV
>Lotharella_globosa_LEX01_c8979_g1_i1
AYISLLMLFALWYFFNIYFNIYNKLVLKSYIPLSLTWMQFAIGVGIVLLMWLVRAHPFYR
AFIVPLALLHVLGNLFTNLSLNAVAVSFTHTIKASEPLFAAFLSWLFL-TPPTCLMIGAL
VPVMVGVGLASFTEATFNWTGFLTAMSSNLSFQTRNVVSKKLMLDNINLFGYITLMSCVI
MTPVMLLFEGFTPAWAEAEFALKSVYAGFFFHTYQQVSYLILDRVTPVTHAVGNCVKRIA
VIVVSIIIFRNPVGLLNAVGITVAMLGVLMYSVA
>Amorphochlora_amoebiformis_CCMP2058_c20422_g1_i1

---LGDWA--------
---HPLV-------LLTLLPI---------------------------------------
---VIGVSLASFTEASFNWMGLGAAMGSNVFFQSRNVISKVVMVDSVALFSMITIISFLI
TLPLALTVE-QAFTALAADIIDKALKAAFCHAYQQVSYAILSRVTPVTHSVGNCVKRVV
VIASSILFFRNPVSPLNLAGAGIALSGVAAYSTV
>Bigelowiella_natans_CCMP2755_Bigna1|37764|e_gw1.21.27.1
-MAVLGVLFLGWYTFNIWFNLFNKQVLSEFVPVSCTGLQFAVGSLLALSAWGLRLQPLPQ
FKLIPIAAVHAMGNILTNVSLGKVAVSFTHTIKALEPFFSVILSGIFLGQW--VVVVVVV
VVIAAGVSLASFTEPSFNWIGLFAALGSNLFFQSRNVVSKLVMIDAINFFSIMTIMSFLI
TIPISGWSLAMK----RADVVTKALYAAFFFHAYQQLSYAILNRISPVTHSVGNCVKRVV
VIASSIIFFQNPVSPLNLAGAGIAISGVYM----
>Arabidopsis_thaliana_OAO90026.1
KVLELGLLFAMWYLFNIYFNIYNKQVLKALAPMTVTLVQFAVGSVLITIMWVLNLYKRPK
IAILPLAVVHTLGNLFTNMSLGKVSVSFTHTIKAMEPFFSVLLSAMFLGEKPTPWVLGAI
VPIVGGVALASISEVSFNWAGFSSAMASNLTNQSRNVLSKKVMLDNITLFSIITLMSLVL
MAPVTFFTEGFTPSYIQSQIYTKSLIAALCFHAYQQVSYMILARVSPVTHSVGNCVKRVV
VIVSSVIFFKTPVSPVNAFGTGIALAGVFLYSRV
>Oryza_sativa_XP_015651257.1
KTLQLGALFGLWYLFNIYFNIYNKQVLKVFYPINITNVQFAVGTVIALFMWITGILKRPK
IAILPLAMVHTMGNLFTNMSLGKVAVSFTHTIKAMEPFFSVLLSALFLGEMPTPFVVLSL
VPIVGGVALASLTEASFNWAGFWSAMASNVTFQSRNVLSKKLMLDNITLFSIITVMSFFL
LAPVTLLTEGVTPTVLQSQIYTRSLIAAFCFHAYQQVSYMILARVSPVTHSVGNCVKRVV
VIVTSVLFFRTPVSPINSLGTGVALAGVFLYSQL
>Physcomitrella_patens_XP_024357874.1
ETLQLGSLFGLWYMFNICFNIYNKQVLKVFYPITITSLQFAVGAVIALLTWFSGLHKRPQ
ILILPLACVHTLGNLFTNMSLGKVAVSFTHTIKAMEPFFSVLLSALFLGDMPNPMVVATL
VPIVGGVALASLTEASFNWAGFLSAMASNVTFQSRNVLSKKFMLDNINLFSIITVMSFFL
LLPVTFFVEGFTPSALAAVVVTRALIAGLCFHAYQQVSYMILAKVTPVTHSVGNCVKRVV
VIVTSVLFFRTPVSPVNGLGTGLALCGVFAYSRV
>Coccomyxa_subellipsoidea_C-169_XP_005646560.1
KTLLLGGLFGGWYLFNIYFNLYNKQVLKVFYPFTCTALQFAVGSLLAVSMWTLNLHEKPK
VSVLPLAVVHTLGNLLTNVSLGQVAVSFTHTIKAMEPFFSVLLSALFLGESPSIPIVLSL
LPVVGGVALASATEATFNWAGFLAAMGSNITFQSRNVFSKKFMLDNINLFSLITILSFLL
LAPIALIRDGLTPSAMQSLVLQRAVFAGFCFHAYQQVSYMILQRVSPVTHSIGNCLKRVI
VIVASVLFFQNPMGRQNMIGTAIALAGVFAYSQV
>Oryza_sativa_XP_015640165.1

RTVQLGAMILVWYLLNIYFNIFNKLVLKSVFPYTITTFQFASGSFFITLMWLLNLHPKPR
LKILPLALVHTMGNVFTNMSLGKVAVSFTHTIKAMEPFFSVLLSVLFLGETPSFLVLGSL
VPIVGGVVLASMTEVSFNWIGFWSAMASNLTNQSRNVFSKKLLLDDINLFSIMTVMSFLL
SAPLMLSVEGFSPSYLQSELCMKAALAGTCFHFYQQVSYSLLARVSPVTHSVANCVKRVV
VIVSSVLFFRTPISPINALGTGVALAGVFLYSRF
>Oryza_sativa_XP_015621423.1
ETAQLGAMIVAWYLLNIYFNIYNKQVLQPLFPYTITAFQLAFGSFVIFLMWALKLHPAPR
IKIAPLAAGHMLGTVFTNMSLSKVAVSFTHTIKASEPFFTVLLSAFFLGETPSLLVLGSL
VPIVGGVALASLTELSFNWIGFWSAMASNLLYQSRNVLSKKLLLDDINLFSILTILSFLL
SLPLMLFSEGFSPGYLRSELCVRAALAGFCFHGYQKLSYLILARVSPVTHSVANCVKRVV
VIVASVLFFRTPISPVNALGTGVALGGVFLYSRL
>Chlamydomonas_reinhardtii_XP_001694243.1
QTLVLGSMFAGWYAANIAFNIYNKQLLKAFFPLTITEAQFLVGSCVTLVAWGSGLQRAPK
INVLPLAVVHTLGNLLTNMSLGAVAVSFTHTIKAMEPIFSVALSALFLGDQPSPLVLATL
LPIIGGVAMASMTEATFNWFGFLSAMGSNLTFQSRNVLSKKLMLDNMALFSVITLLSAAL
LLPATLLFEGLSPVGLAEGVLAHAAMAGLCFHLYQQVSYMILSRVSPVTHSIGNCVKRVV
VIAASVLFFRNPVSLQNALGTALALAGVFLYGTV
>Ostreococcus_tauri_XP_003077968.1
QVLMLLMLFGCWYGFNIVFNIYNKQILKTFYPVTVTLIELGVGSALIAAMWASGAKKPPQ
VPIAPLAVIHAVGNLLTNVSLGKVAVSFTHTIKASEPFFSVLLSALFLGDVPSLAVMAAL
LPVVGGVALASMTEVSFCWAGFLAALGSNITFQSRNVLSKKMMIDNINLFSVITMLSCLV
ALPVAIGVEGFTPAAIAAELSKSLLVAGFCFQMYQQISYMILSRVSPVTHSVGNCMKRVT
VIVVTLIYFKNPVSPLNMAGTAMALTGVFLYSRA
>Chlamydomonas_reinhardtii_XP_001696294.1
GMMVLGLMFVAWYGTNIFFNIYNKQLFKVFFPLTTTNIQFFIGSCLSMVFWVTGIVKLPK
ISIYPLAIINVLGNVLTNVSLGHVAVSFTHTVKAMEPFFSVIFSAIFLGDVPPVPVLLTL
VPIVGGVVIASLTEATFNWTGFLSAIFSNMTFQSRNVLSKKLMVDNMNLFQIITIMSFLM
LLPVSTMVEGLTPESLANQMFMRLLSAGICFHSYQQLSYMILSRVAPVTHSIGNCVKRVV
VIVASLIAFQNPISMQNAIGTGIALFGVFLYSQA
>Helicosporidium_sp._ATCC_50920_KDD72777.1
STLLLGALFGAWYLFNIYFNIYNKQVLKAYYPLTVTAFQFLVGSALSLGMWGLGLHAPPV
VTLSPLALVHTLGNALTNVSLGAVAVSFTHTIKALEPLFSVVLSSLFLGDAPNAAVILSL
VPIVGGVALASTSELSFNWTGFLSAMGSNLTFQSRNVLSKKFMLDNVNLFSVMTLMAFCM
LAPLALFLEALTPAAVRH---------------------LVGGRAAAALDPIAT------

>Erythrolobus_australicus_CCMP3124_c2436_g1_i1

GKALLAVYIGCWYAANILFNIFNKRLLKIVLYTTVTLFQLAMGGLVGCALWATRLHKYQK
VAIYPLALAHLAGNLFTNLSLRQMAVSFTHTIKASEPFFSVVIGKVFVGTAFHPAVYASL
VPIVAGVTLASVSEVSFNWVGFLTAMASNVSFQSRNVLSKKFMLDNLNLFAWISILSCAT
LAPFALVLDAYGGVAAAAPLLSKLCVCGFLHFLYNQFSYVVLQRVNPVTHAVGNTMKRVA
VIASSILVFRNPVTKLNLLGTAIAIAGVAIYSQV
>Madagascaria_erythrocladioides_CCMP3234_c38259_g1_i1
-------------------------MLKRFLFTTVTLFQFAMGAVAIALLWLLRLCEFKR
PAIYPLAIAHLLGNMFTNLSLRQMAVSFTHTIKASEPFFSVGISKIFIGTAYHPLVYLSL
VPIVLGVTVASVTEVSFNWVGFTTAMCSNVSFQSRNVLSKKFMMDNLNLFGWISILSFLT
LLPFNLILEALVQTLSAA-ILTALVACGFLHFLYNQFSYVVLAMVNPVT-----------

>Madagascaria_erythrocladioides_CCMP3234_c12584_g1_i1

-------------------LGFATALSSNVAFQSRNVLSKKCMMDNLNLFGYISILSFLT
LLPFTLFLEGYASVFGGAGLLATLCLCGFLHFLYNQFSYVVLQRVNPVTHSVGNTMKRVA
VIVSSIIVFKNEVTTLNKIGTAIAIFGVGLYSQV
>Galdieria_sulphuraria_XP_005703604.1
KLIALTFYIGCWYAANILFNIYNKRVLKVFLFATVTLVQFLMGSLVGLALWISGLHRFQK
AKIYPLALSHLIGNVLTNVSLRQVAVSFTHTIKAAEPFFSVALSKLFIGTAYTIWVYLSL
IPIVGGVTLASISEVSFNWIGFLTAMASNVAFQSRNVLSKKFMFDNLNLFAYISILSFVT
MLPFTLLLEAWREMASVAVLLLRIAIAGFLHFLYNQFSYVVLKRVNPVTHSVGNTMKRVA
VIVSSVIVFKNQVTLLNKIGTAIAIAGVAIYSQV
>Porphyra umbilicalis_OSX75555.1
RTVAIGLSIALWWASNVKFNIANKRLLKVFLFLTLTTVQFGMGAVVAILAWSLRLRKWQT
PALYPLALVHVLGNVFTNLSLRAMAVSLTHTLKASEPFFSVVISKLFIGTVYTGAVYASL
VPIVFGVTMASVSDITFSWFGFFTAMGANVAFQSRNVLSKRFMMGNFNTFAWISILSFLT
LAPFSLLFDAWSSTLTAAAVALALANVGFLHWLYNQASYLVLVQVNPVTHAVGNTMKRAA
VIISSVLVFGNTLTTTNKIGTAIALIGVGLYSQV
>Rhodella_maculata_CCMP736_c12961_g1_i1
SKVVLAGLILCWYASNIMFNIVNKKLLVAFLFPTVTLIQFMMGAVAVSTLWLLRVVKFQR
AAILPLAVAHVLGNIFTNIAIRQMAVSFTHTVKAAEPFFSVIIAKTFLGATFTPYVYASL
VPIVIGVTLASANEVSFSWPGFITAIAANLAFQSRNVLSKKFMLDQVNLFGYISMLATLI
MLPITLVVDSWGPAITAASLLLTLMFCGLYHYGYNQFSFLVLRRVNTVTHAVGNTMKRVV
VIISSILVFQNPVSDLNKLGTALAILGVGIYSQV
>Cyanidioschyzon_merolae_strain_10D_XP_005537432.1

RRLALGAYIACWYAANIGFNIVNKTLMKSFLFVSVTAVQMLAGATISLFLWGTRMHRFQR
AKIYPLALAHLFGNLFTNFSLRQMAVSFTHVIKASEPFFSVVLAKIFLGTTFSWPIYASL
VPIVFGVVLASVSEVSFNWPGFLTAVASNVSFQSRNVLSKKFMFDDVNLFGWISCLAAIT
AIPLAIVVDYYAGVWSAASLLGMLALCGLLHYLYNQFSYVVLQRVSPVTHSIGNTVKRVA
VIVSSVLFFRNPVSRQNIIGTVIALAGVAIYSQV
>Ostreococcus_tauri_OUS48046.1
PTFTLVASFGGWYYFSIAFNIYQKALLKAVMPWTVTALELLIGSALVAATWGVRLKRAPE
CAVGVLGTVHFLGNALTNVSLGKVAVSFTHTVKALEPVFSVGLSAAFLGAIPSLALCASL
IPIIAGVMIASATEVSFNMAGFLSAMGSNLTFQSRNVLSKMFMLDYYNLLGVLTIASTVI
AIPVALATEFMTLANVTATVGFNLVMAALCFQLYQQLSFSVLERVNPVTHSVGNSLKRVI
VIAASVLIFRNPVSATNIGGTALAIFGVILYGQV
>Compsopogon_caeruleus_SAG-36.94_c4181_g1_i1
SKLVLIALFLGWYASNTIFNIFNKQVLREFFPVTCSTIQCFIAGCTMALLWLLRLKDTPP
IATVPLAVLHACGFTLTNMSLGKVSVAFTHTVKATEPFFSVALSPSILGEVPTWGKLLSL
FPIVAGVALASATEASFDWNGFLSAMGSNLAFQSRNVLSKRFMLDNVNLFAVMTIVAFTM
MVPFAFFIDGIVTLLGAGKLLRYLILGGITRCMDVLSSYMILNRVSPVSHSVGNCVKRAV
VIGASVIFFKTAMSTLNMFGTAIALFGVLVYSLV
>Madagascaria_erythrocladioides_CCMP3234_c37704_g1_i1

----------------------------------FKRF----------------------
-------------------------------------MGKRNTLDNVNLFAVMTIVAFFM
MVPVVALVDGFTPAVLRAKLIRLLVLGGLARCIDVLSSYMILNRVSPVTHSVGNCVKRAV
VIVVSVIIFKTKMTVWNIVGTTTALTGVLIYSLV
>Gracilariopsis_chorda_PXF47301.1
ATIVLMLMFASWYWANTAFNVYNKQVLKVFYPLTCTVVQFAVAALVMAMTWITRMKKPPK
LAALPLACLHAAGFLLTNMSLGKVSVAFTHTVKSTEPFFSVALTPSILGDVPTWGILVSL
FPIVAGVGLASAADVSFNWIGFLSAVGSNLALQSRNVLSKRLMLDNINLFSTMSILAFFV
LIPICLLWEGLGAASFQARLYVMLIMGGICRCLDVLISYVILKRVSAVTHSVGNCVKRAV
VIVASVFVFKTKMTMLSIIGTTMALTGVLIYSLI
>Chondrus_crispus_XP_005714035.1
ATLLLMALFVLWYWSNTVFNVYNKQVLKVFYPLTCTVVQFAVAGGVMAAFWLFRLKKPPK
LFATPLAVLHAAGFLLTNMSLGKVSVAFTHTVKSTEPFFSFALTPSILGDIPTWGILVSL
FPIVAGVALASANEVSFNWIGLFAALGSNLALQWRNVLSKKLMLDNVNLFSTMSILAFFV
LLPICFFWEGAGAAVSQARLYRMLTVAGACRCLDVLSSYMILRKVSPVTHSVGNCVKRAV
VIATSIIIFKTKVNALNIAGTLLALFGVFMYSII
>Erythrolobus_australicus_CCMP3124_c4974_g1_i1

SAAVLAMLFGMWYYTNTVFNVYNKRVLNVYFPYLCTTVQFLVASILMATLWLTGLKKAPA
LAVLPLSVFHAAGFLLTNVSLGAVSVAFTHTVKATEPFFSVALSPSILGEVPTWGILGSL
FPIVAGVVLASATEASFTWTGFLSAMGSNLMLQLRNVLSKRVLLDNINLFAFMSMLAFAL
MVPVSLLVEGFRAPMEAADLVRMLMMGGLCRC-----------------------------
----
>Timspurckia_oligopyrenoides_c3441_g1_i1
ELLILCALFLTWYYTNTVFNLYNKQVLKTFYPITCTALQFLVASLLMMLLWVFKLQKPPQ
LAVAPLALLHALGFLLTNMSLGSVSVAFTHTVKATEPFFSVALSPSILGVVPTWGVVGSL
FPIVAGVALASATDVSFTWLGFLSAMGSNLALQSRNVLSKKVLLSNVNLFAVMSMFAFVM
MAPVALAVEGLSGSAVAAELWKLLAMGGLCRCGDVLASYMILNRVSPVTHSVGNCVKRAV
VISMSVIVFQTPMSVLNIAGTVLALLGVLMYSLI
>Rhodosorus_marinus_UTEX-LB-2760_c6890_g1_i1
RILLLGGLFVSWYASNTAFNVFNKQVLKVYYPLTCTMLQFLVGSIMMASLWVFRLRRVPV
VTLGVLSALHASGFYLTNASLGSVSIALTHTVKSTEPFFSVALSPSLLGEVPTWGVLATL
VPIVFGVGIASATEASFTWFGFVTAMGSNLCLQGRNLISKRFMIDNVNLFSLISIGAFLI
LLPISSIAEGAVSAIRALTLLGKLFYGGFFRCVDVLTSYMILKQVSPVTHSVGNCVKRAI
VITSSVILFRTAISPVNIFGTSMALMGVLVYSLV
>Rhodella_maculata_CCMP736_c19039_g1_i1
KYCVLGLLFFIWYSSNTIFNVYNKVALQAFYPLTVSVVQFGVSALFCCALWLLKIQTPPR
LLALPIAALHSAGFLLTNTSLSSVSVAFTHTVKATEPFFSAALSPIILRTVPTWGPMLAL
FPIVGGVALASFTELSFTWVGFLSAVGSNVAFQSRNVLSKKIFLDNVNLFALMSIIGMAI
LAPFSMA---VFTPFVATELARLLFLGGICRCADVLVSYMILQRVSPVSHSVGNCMKRAI
VICSSVVIFQTSMPFLNVVGTVIALVGVLVYSLV
>Ectocarpus_siliculosus_CBN74592.1
---------------------MRPTRLTALLPFTLAAIQLLVGVPYVWMLWLTGVRKAPE
LGTTPVAMAHTMAHLAAVVSIGAGAVGFVQ-------------------------VYTTL
LPVVGGVAMASAGEISFSALAFGAAMTSNASAASRSVLGKIFMMCAGNLYAVMTMLGCLV
LTPAALWVEGVASVWNAASLVKNVLLSGVFFYLYNEVSFYALNIIHPVTHALGNTLKRVV
MIIVSVLVLNHRFTPLGLAGCTTAIGGVMAYSLT
>Chromulina_nebulosa_UTEXLB2642_TR15436-c0_g1_i1
DILNLIVLFVLWYGFNAGYNVYNSYVKNDLFPFFVATIQLAVGLLYAVPLWVLGIRASPR
LKLFPIVALNAAGHLAAVIAMGGGS--FTHVIKASEPVVSVILGLFINGVVPKPLTALSL
LPITYGVAYASTLGNELTTKAAMLAMASNVAFALRSIFRKNL-LTPANEHAITTILSTIL
LTFVFIGYEPIASRFEAITFYKNIIFCGMSFYLYNEMQNMVLGSLGAVPTAVGNTLKRVV
IFVALYFTEGETFPLPKVIGCAIAIVGCLLFAVF
>Chromulina_nebulosa_UTEXLB2642_TR1248-c0_g1_i1

----------------AVNAMGGGS--FTHVIKASEPVVSVIFGILINKAIPKPLTALSL
LPITYGVAYASTLGNELTTKAAKMAMGSNVAFALRSILRKNL-LDPANEHAVATFLSFLL
LIPFALYFEGIRNAYNTITFLINTGLCGISFYLYNEMQNVVLGSLGPVPTAVGNTLKRVV
IFVALYFTSG------------------------
>Florenciella_parvula_CCMP2471_c2817_g1_i1
RKVRIVSFLTLWYAFNAGFNVTNKQLLNQFFPWVVSWFQLAIGLLYVLPAWKTGIKKPPK
VSFLPISLLHAGGHMSQVASMGLGSVFFTHVIKASEPVIGTFVVLAFTGKIAPWYVNLCL
APVVGGVAYAAMKPGLLWSAPSLLAFASTVAFAIAKLLAKNQMLDAANNYALLTCCSSVL
FLPSLLV-EGALAAFNAMQFILDLLRCGAFYYAYNEMGFRVLDMLSQVSAALANSAKRVV
ILFAAVFFLGESVTQRKLIGSTIAITGVTMYSVA
>Pseudopedinella_elastica_c46278_g1_i1
KWCQLSFFLVLWYAFNVGFNVSNKLVLNQFYPWVVSWVQLATGLGFVLPMWFSGLRSKPR
VKFLPVALLHSSGHAAQVAAMGLGSVFFTHVIKASEPFIGTLVVLLFTGKIAPWYVNLCL
IPVVGGVAFAAMKPGYLWAAPSLLALASTVAFAIAKLLAKNLMLYAANNYAVLTCCSSVL
FLPSMIA-EGALAAFQAFAFSKDLLVCGGLYYAYNEMGFRVLDLLGPVSQAVANSAKRVV
IMLAAVLFLGESVTQRKLIGASVAIAGVTLYSLA
>Aureococcus_anophagefferens_XP_009032193.1
----LLSFIGLWYAFNAFFNVQNKLILNQFYPWVVSWFQLASGLLFVLPMWFTKLRAPPK
VKFLPIAALHCGGHGLQVSSMGAGSVFFTHVIKATEPVIGTLVLLAFTGKIAPWWVNACL
TPIVGGVAYAAFKPGDLVGYASLAALGSTVAFSIAKLLAKSLMLTAPNNYAFLTICSTLL
LLPSALG-EGALAAFQQMAFARQLVACGFLYYGYNEMGFRVLDLLSPVSAAVANSLKRVA
ILLAAVLFLGEQVSTRKIIGSSVAMGGVLLYSLA
>Dictyocha_speculum_CCMP1381_c38541_g1_i1
RAPELSFLLLLWYAFNAGFNVSNKRLLNQFHPWILSWVQLATGILFVVPAWLSGARTAPI
VKFLPIALLHAGGHCSQVASMGMGTVFFTHVIKAAEPVIGTLVVLLFTGKLAPWYVNLML
TPIVGGVAYASIKPGSLVSTSALLALVSTFAFAIAKLLAKNLMLNPANNYAVLTCCSSVL
LLPSFIA-EGALADLAALGFILEAIGCGMLYYMYNEMGFRVLNLLTPVSAAVCNSAKRVV
VLFAAVVFLGEAVSKKKLIGSTVAIGGVTLYSVA
>Phaeocystis_antarctica_CCMP1374_c16515_g1_i1
STTSTIVAVLAWYAFNGVFNVENKRLLNELFPWILSWVQLATGILICVPAWLLGLRRAPV
VRFAPIAVLHASGHALQVAGIGAGSVYFGTVIKATEPLIGTLIALVVEGKVAPWYVNLTF
LPIVGGVAYAAAKPGELMSFAALAAFSSTVFFAIAKLLAKRIMLDAVNTYSLLTCCSTLL
LLPSFYA-EGALAALQSARLSARLLLCGFCYFAYNECGFRVLDAFGPVSQAVANSAKRIV
ILFFAVYFLGETASAQKLVGAGVAIGGVTCYSLA
>Emiliania_huxleyi_CCMP1516_XP_005774470.1

AALDTAIPLLAWYAFNGAFNVENKRVLNELYPWIISWVQLAAGIGIAVPAWLLGLRRPPV
MRFLPIAVLHAAGHALQVAGIGAGSVYFGTIIKATEPLIGTLIAYAVDGKAAPWYVNLTF
VPIVGGIAYAAAKPGDLASFAAVAALSSTVFFALAKLLVKRLMLDAANTYSVLTCCSALL
LAPS-------------------LLLCGLYYFAYNECGFRVLDKLSPVSQAVANAAKRLV
ILFFAVVFLGEEASGRKLVGAGVAIAGVTSYSFA
>Ectocarpus_siliculosus_CBJ33275.1
GVGSLAVLFVMWYGFNAYYNISNKMVT-------------VIGLVYLIPMWASGMQKVPK
LKLLPISILHAGGHLAAVLSMSAGAVSFTHIIKASEPVASTVIGPFFGVEVQPMTVNMFL
LPIVGGVAYAAMKPGQLTNLASGYAMASNIFFAIRGILSKQVMMSASNTYGVLTIMSVIL
VLPMLFF-EGSKDAFDDVTLLKTLLGCGISYYLYNEMGFRVLNRLDPVSSAVGNTVKRVV
IMGAAVLFLGEEMNANKLIGACIAVAGTLAYSLA
>Alexandrium_catenella_OF101_c5597_g1_i1
EKFKLPVFVGLWYFFNVQYNIQNKKLLSCFATWAVSWIQLSAGIPIAMLMWGSGILTVPK
VKLAPVGAAFAAGQALTVASLGAVAVSFTHVVKALEPAVNAIASAFLLGQVFHPLVYVSL
APIFIGVGLASSAELSFTMFGFATAMMSNFAFVFRNVLATKFGTRKTNQLAVLTAVATLV
LLPVALVLPGIPSSWAKTSLLYLMLSSGFHFFMYQMSSFWVLSCVQPITHSVLNTLKRVV
IIIVSILVFRNPVTAQSVAGTGIAIAGVLIYSLT
>Protoceratium_reticulatum_CCCM-535---CCMP-1889_c6950_g1_i1
--WLVGFVQVPK
VKLSPVGAAFAAGQVATVASLGAVAVSFTHVVKALEPAVNAIASALILGQVFHPMVYASL
APVFIGVALASSSELSFTMFGFLTAMASNFAFVSRNVLATKFGTRKTNQLAVLTAVATLV
LLPLALVLPGAPQAWSAATLAYLMLASGFHFFMYQLSSFWVLSCVQPITHSVLNTLKRVV
IIIVSIFVFRNPVTAQSALGTATAIGGVLLYSLT
>Amphidinium_massartii_CS-259_c54681_g1_i1
SAVKLPVYVCLWYFFNVQYNIQNKLLLKAFASMAVSWIQMAAGIPIAGLMWMTGLLKKPK
VKLAPVGAAFAGGQVATVASLGAVAVSFTHVVKALEPAVNAVASAFLLGQVFHPLVYCSL
LPVFAGVALASASELSFTAFGFATAMASNFFFVTRNVLATKFGTRKTNQLAVLTAVATAV
LLPLALLAPGMPAAWAKATLLWLMASSGFHFFMYQMSSFWVLSCVPPITHSVLNTLKRVV
IIVMSIIVFRTPVTAQGLAGTGIAIGGVLLYSLT
>Symbiodinium_microadriaticum_OLQ09465.1
DKIKLPVYAGLWYFFNVQYNIENKKLLNVFANWAVSWVQLAAGIPIALFMWSTGLVKAPK
MKLAPVGAAFAAGQVATVASLGAVAVSFTHVVKALEPAVNAIASALVLGQVFHPMVYASL
LPVFAGVALASSKELSFTMFGFLTAMASNFFFVTRNVLATKFGQRKTNQLAVLTFVATMV
LLPVCLFLPGIPSAWNAG---
-----------PSENEASE---------------
>Pavlova_gyrans_CCMP608_c6036_g1_i1

-----------------------------QVVKAGGPVWTVALSALLLREKVSARVALSL
VPIMAGVGLATLKELSFAWAALLGAVVSDVAFALRNVLSKKSMVSPANLFGILTISATVV
LVPLAAVAEWMGPAWHAAQLLAELVAAGAFFYAYSEVAMQALANVSPVTHAIANTLRRVV
IMLVSALVFSTRMTTLGMVGSGLAIAGSYAYSAA
>Plasmodium_falciparum 3D7_XP_001351856.1
EVGKTVSLLGMWYVCNIFYNIENKKALNILMPITIAITQIYVGLPIFLIPWLLKLRNQPE
LSIMKQSIYHGYAHLLSVIAMGAGAISFVHIVKASAPLFAAFFSYFFMNNKMSIYTYSSL
VPIVFGVSLASIKELSFTYKALYSTLSANVLSTMRAIEAKIMMLTPENIFALLTLSSAIF
LTP-ALYID--SHKWKDAVLGRHVLMSGVWFYLYNQLSFISLNRLNHITHAVASTVKRVF
LILTSYFIFGTKFSFLGGLGSSIAVGGTFVYSLV
>Fibrocapsa_japonica_CCMP1661_c4567_g1_i1
ELVNILLYLSGWYFLSLIFNIYTKEALEIVAPWTISAAQLVIGSFYISILWLGRVRKFHP
VKVVPIAFYNSASLVFCVLGFSSGTVSFVHVIKATEPLFAAILSWWIMNEALPWKVYLSL
LPVVIGVAMVSAKEVHFSWSSLAGALLSSLCGALRTIAAKNAMMDPCETLAWTTILGGLM
LLPLVVIFEGWQEGAKLTNFLGTVIASALGLYTYEECAFLFLNATSPVTQTIGNAMRRVF
LVGISIFWFATPVTTFSVIGSMVAIAGVGLYSVL
>Pseudopedinella_elastica_c2415_g1_i1
QTAKVGFLFSLWYGLNVIYNVVNKKVLNRLLPWLVAVAQLGVGALYASAAWASGLREAPE
QALAPIGVAHGVGQVATVLSLGAGAVSFTHIVKALEPFFSAIVSAITGEGWLRPQVYATL
LPVVGGVSVAVLKELSFSWLAFLTALGSNVAFAYRAVLSKRAMLSAASLYGVVT------

>Ectocarpus_siliculosus_CBJ49109.1
QTVIVGIYFFLWYALNIGYNITNKKALNAILPWSISVLQLVVGSIFVLPLWMLKLRDAPG
LGLSPIATCHMLSHVCAVIGLGAGAVSFVHIVKAAEPLFTALFSAVFLGQIFSPLVYLTL
VPVVAGVALASLKELDFKWAALGGAMGSNLAASTRAILSKRSMMSPANLYAVLTIMASAM
LLPLSAMVEGIKELWESTEIIYNTVASGVFFYLYSHWGGGGLGEQGPLPQGGGTPKKQKT
PIF---FFLGEGGKPPPPHSPPLSRGGVGFLPLG
>Phaeocystis_antarctica_CCMP1374_c20219_g1_i1
TVSKIAINVFLWWTLNVVFSLCNKQCLNSWHPWALACSHLAIGTLCMLPLYIVPTRQVPR
LTLLPVAALLSVGHVTSTLAPAYGTVAFSNIVKTAEPLFTCACSMVLYRRLYSTPVYLAL
LMVVSGVALVSCRDVNFSSFSLMAGMISNAAFALYSIYAKRAMLTPRTAYALLTMGSLTM
LTPLALLME-LASASTAVRLAALLGFTGLVQYVSNEIAFCTL--------S---------

>Symbiodinium_microadriaticum_OLP87735.1

RTLKLGALFVLWYALNIGYNIGNKLVLTALTPWSSATWELFFGLPYVGFLWLTGLRKRPE
LLLIPPAFFLACTHVFGVISFGAGAISFTHVCKATEPVWTALISALVFREFLPVPVYLSL
IPIIFGVSLASAHELSFTWISFIAATGSAVTSASKAILGKKVLLTPGNMFAVLTILGCFM
ILPASMLIEPFSAAWAHARLWIMLSVSGFLYYTYNEVAFLAL------------------

>Vaucheria_litorea_CCMP2940_c15550_g1_i1
QTLKVGSYFGLWYLLNIGYNIYNKTSLNMLLPYFIATVQMASGLIYVIPLWLTGLRKAPK
INLSILGFLHMMTHITAVLSLGAGAGSFTHIVK---------------------------

>Vaucheria_litorea_CCMP2940_c15801_g1_i1
PRVKVLGYFALWFLLNAYYNIANKKTLNMVLPWIVSSAQLIIGSIYICFVWLLKIRPAPK
VAVLPLAALHTTSHISAVI---

>Fibrocapsa_japonica_CCMP1661_c1177_g1_i1
RRLKVGSAFLSIYSIDLVYYIFHKRALTVFFPWTIVSAQFAVGYLVFLPIWAFGLRKRPK
LALLPVSFAIVAHHIVSTLMVHFGTVSFSHIVKASEPFFAAAYAALVNSQVFAWPVYAAL
LPVVGGVGVACTQEPSFSMNALLCGMATNTFSTLRVALSKDLMLDSGNLNAVTVLMAAVL
SLPLAFLVEGVVAGWGAS-VARVQAAGGI-------------GAAAGVA-SLGQGLSGLG
YWQASW-----------MIARDIMIAGFL-----
